# Supplementary material for: Global reporting and underreporting of occupational diseases: A systematic review
Source: PLoS One. 2026 Mar 26;21(3):e0345318. doi: 10.1371/journal.pone.0345318 (PMC13020801; doi:10.1371/journal.pone.0345318)
Supplement: S1 File — (DOCX) [file pone.0345318.s001.docx]

**Search strategies for electronic databases**

| **Database / date** | **Strategies** |
| --- | --- |
| PubMed  May 2^nd^, 2023  (n = 1,856)  Oct 28^th^, 2024  (n = 1,897) | 1. underreport*[Title/Abstract] OR under report*[Title/Abstract] OR unreport*[Title/Abstract] OR misreport*[Title/Abstract] OR "capture-recapture"[Title/Abstract] OR underestimate*[Title/Abstract] OR under estimat*[Title/Abstract] OR undercount*[Title/Abstract] OR under count* [Title/Abstract] OR underrecord*[Title/Abstract] OR under record*[Title/Abstract] OR mandatory reporting[MeSH Terms] OR disease notification[MeSH Terms] OR population surveillance[MeSH Terms] OR public health surveillance[MeSH Terms] OR sentinel surveillance [MeSH Terms] OR insurance claim reporting[MeSH Terms] OR public reporting of healthcare data[MeSH Terms] 2. "occupational diseases/epidemiology"[MeSH Terms] OR "occupational disease"[Title/Abstract:~2] OR "occupational illness"[Title/Abstract:~2] OR "work disease"[Title/Abstract:~2] OR "work illness"[Title/Abstract:~2] 3. #1 and #2 |
| EMBASE  May 2^nd^, 2023  (n = 1,399)  Oct 28^th^, 2024  (n = 1,464) | 1. (underreport* or under-report* or misreport* or unreport* or "capture-recapture" or underestimate* or under-estimat* or undercount* or under-count* or underrecord* or under-record* ).ti,ab. OR mandatory reporting/ OR disease notification/ OR population surveillance/ OR public health surveillance/ OR sentinel surveillance/ OR workman compensation/ OR “public reporting (health care)”/ 2. occupational disease/ep OR ((occupational or work) adj3 (disease* or illness*)).ti,ab. 3. *#*1 AND #2 |
| CINAHL  May 2^nd^, 2023  (n = 760)  Oct 28^th^, 2024  (n = 804) | 1. (TI underreport* OR under-report* OR misreport* OR unreport* OR “capture recapture” OR underestimate* OR under-estimat* OR undercount* OR under-count* OR underrecord* OR under-record*) OR (AB underreport* OR under-report* OR misreport* OR unreport* OR “capture recapture” OR underestimate* OR under-estimat* OR undercount* OR under-count* OR underrecord* OR under-record*) OR (MH “mandatory reporting” OR “public reporting of healthcare data” OR “population surveillance” OR “disease surveillance” OR “worker’s compensation”)   Note: The subject headings of following terms: “disease notification”, “public health surveillance”, “sentinel surveillance”, “workman compensation” were not found in the CINAHL subject headings.   1. TI ( occupational disease* OR occupational illness* OR work-related disease* OR work-related illness* ) OR AB ( occupational disease* OR occupational illness* OR work-related disease* OR work-related illness* ) OR MH (“Occupational Diseases”) 2. *#*1 AND #2 |
| SCOPUS  May 2^nd^, 2023  (n = 649)  Oct 28^th^, 2024  (n = 653) | 1. ( TITLE ( underreport* OR under-report* OR unreport* OR misreport* OR "capture recapture" OR underestimate* OR under-estimat* ORundercount* OR under-count* OR underrecord* OR under-record* OR "mandatory reporting" OR "disease notification" OR "disease surveillance" OR "population surveillance" OR "sentinel surveillance" OR "public reporting of healthcare data" OR "worker’s compensation" ) OR ABS (underreport* OR under-report* OR unreport* OR misreport* OR "capture recapture" OR underestimate* OR under-estimat* OR undercount* ORunder-count* OR underrecord* OR under-record* OR "mandatory reporting" OR "disease notification" OR "disease surveillance" OR "population surveillance" OR "sentinel surveillance" OR "public reporting of healthcare data" OR "worker’s compensation" ) ) 2. ( TITLE ( ( occupational W/2 disease* ) OR ( occupational W/2 illness* ) OR ( work W/2 disease* ) OR ( work W/2 illness* ) ) OR ABS ( ( occupationalW/2 disease* ) OR ( occupational W/2 illness* ) OR ( work W/2 disease* ) OR ( work W/2 illness* ) ) ) 3. #1 AND #2 |
| Web of Science  May 2^nd^, 2023  (n = 577)  Oct 28^th^, 2024  (n = 580) | 1. **TI = ((underreport*) OR (under report*) OR (unreport*) OR (misreport*) OR (capture recapture) OR (underestimate*) OR (under-estimat*) OR (undercount*) OR (under-count*) OR (underrecord*) OR (under-record*) OR (“mandatory reporting”) OR (“disease notification”) OR (“disease surveillance”) OR (“population surveillance”) OR (“sentinel surveillance”) OR (“public reporting of healthcare data”) OR (“worker’s compensation”)) OR AB = ((underreport*) OR (under report*) OR (unreport*) OR (misreport*) OR (capture recapture) OR (underestimate*) OR (under-estimat*) OR (undercount*) OR (under-count*) OR (underrecord*) OR (under-record*) OR (“mandatory reporting”) OR (“disease notification”) OR (“disease surveillance”) OR (“population surveillance”) OR (“sentinel surveillance”) OR (“public reporting of healthcare data”) OR (“worker’s compensation”))** 2. **TI = ((occupational NEAR/2 disease*) OR (occupational NEAR/2 illness*) OR (work NEAR/2 disease*) OR (work NEAR/2 illness*) OR AB = ((occupational NEAR/2 disease*) OR (occupational NEAR/2 illness*) OR (work NEAR/2 disease*) OR (work NEAR/2 illness*)** 3. #1 AND #2 |
| Dimensions  May 2^nd^, 2023  (n = 7,199)  Oct 28^th^, 2024  (n = 7,441) | ((underreport*) OR (under-report*) OR (unreport*) OR (misreport*) OR (capture recapture) OR (underestimate*) OR (under-estimat*) OR (undercount*) OR (under-count*) OR (underrecord*) OR (under-record*) OR (mandatory reporting) OR (disease notification) OR (disease surveillance) OR (population surveillance) OR (sentinel surveillance) OR (worker’s compensation))  AND  ((“occupational disease”~2) OR (“occupational illness”~2) OR (“work-related disease”~2) OR (“work-related illness”~2)) |
| WHO IRIS  May 2^nd^, 2023  (n = 20)  Oct 28^th^, 2024  (n = 20) | Title (occupational diseases AND report)  Database was last updated in 2018. |
| Google Advanced Search  May 2^nd^, 2023  (n = 19)  Oct 28^th^, 2024  (n = 20) | underreport* AND (occupational disease*) site:.org filetype:pdf  underreport* AND (occupational disease*) site:.gov filetype:pdf |
