# Supplementary material for: Global reporting and underreporting of occupational diseases: A systematic review
Source: PLoS One. 2026 Mar 26;21(3):e0345318. doi: 10.1371/journal.pone.0345318 (PMC13020801; doi:10.1371/journal.pone.0345318)
Supplement: S2 Fig — (DOCX) [file pone.0345318.s002.docx]

**Identification**

Studies from other sources (n =20)

Google advanced search (n = 20)

Studies screened (n = 8,877)

Studies assessed for eligibility

(n = 425)

Studies removed (n = 7,485)

Duplicates identified manually (n = 64)

Duplicates identified by Covidence (n = 7,421)

Studies excluded (n = 8,452)

Studies excluded (n = 295)

Ineligible study type (n = 78)

Ineligible study outcome (n = 42)

Ineligible study population (n = 12)

Ineligible setting (n = 69)

Ineligible indication (n = 8)

Article not in English (n = 21)

Full text unavailable (n = 65)

**Included**

Studies included in qualitative synthesis (n = 127)

**Screening**

Studies from databases/registers (n = 16,342)

Dimensions (n = 10,495)

PubMed (n = 1,950)

Embase (n = 1,551)

CINAHL (n = 832)

Web of Science (n = 690)

World Health Organization (WHO) (n = 500)

Scopus (n = 324)

Studies analysed for both objectives (n =1)

**Fig 1. PRISMA flow diagram of study selection**
