# Supplementary material for: Global reporting and underreporting of occupational diseases: A systematic review
Source: PLoS One. 2026 Mar 26;21(3):e0345318. doi: 10.1371/journal.pone.0345318 (PMC13020801; doi:10.1371/journal.pone.0345318)
Supplement: S3 Table — (DOCX) [file pone.0345318.s003.docx]

**Table 1. Summary characteristics of all studies**

| Characteristics | n of articles |
| --- | --- |
| Year published   - 2019 – 2024 - 2013 – 2018 - 2007 – 2012 - 2001 – 2006 - < 2001 | 30  30  32  20  15 |
| Region   - Europe - Asia - North & South America, Canada - Australia - Africa | 98  16  9  2  2 |
| Industrial sector   - All sectors - Agriculture - Mining and quarrying - Manufacturing - Construction - Healthcare - Service - Others | 92  7  3  6  3  10  2  5 |
| Type of diseases   - General / non-specific occupational diseases - Skin diseases - Cancer - Respiratory diseases - Occupational asthma - Musculoskeletal disorders - Infectious diseases - Asbestos-related diseases - Mental illnesses - Hearing damage/loss - Poisoning - Chronic condition - Assessed more than one disease | 36  20  17  13  10  9  8  7  3  2  1  1  7 |
| Data sources*   - Physician reports - Occupational health registries - Workers’ compensation claims - Others (cancer registries, labour surveys)   **exclude studies focusing on factors contributing to underreporting* | 43  40  23  17 |
