# Supplementary material for: Global reporting and underreporting of occupational diseases: A systematic review
Source: PLoS One. 2026 Mar 26;21(3):e0345318. doi: 10.1371/journal.pone.0345318 (PMC13020801; doi:10.1371/journal.pone.0345318)
Supplement: S4 Table — (DOCX) [file pone.0345318.s004.docx]

**Table 2. Characteristics of each included study**

| No | Author | Year | Category of study design | Time period of data collection | Type of illness | Country | Industrial sector |
| --- | --- | --- | --- | --- | --- | --- | --- |
| 1 | Murphy PL(1) | 1999 | Quantitative descriptive | 1987-1995 | Occupational low back pain | United States | All |
| 2 | Kraut A(2) | 1994 | Quantitative descriptive | 1989-1991 | All | Canada | All |
| 3 | Nordman H(3) | 1999 | Quantitative descriptive | 1990-1995 | Occupational asthma | Finland, Sweden, UK, Canada, US | Food, manufacturing, electronic, wood working industry |
| 4 | Pelclova D(4) | 2007 | Quantitative descriptive | 1991-2005 | Mesothelioma | Czech Republic | Asbestos exposed industries |
| 5 | Ross DJ(5) | 1998 | Quantitative descriptive | 1997 | work related and occupational respiratory diseases | UK | Latex-related industries |
| 6 | McDonald JC(6) | 2006 | Quantitative descriptive | 1996-2001 | work related skin diseases | UK | All |
| 7 | Chen H(7) | 2013 | Quantitative descriptive | 2001-2010 | Coal miners' occupational disease | China | Coal mining industries |
| 8 | Toren K(8) | 1996 | Quantitative descriptive | 1990-1992 | Occupational asthma | Sweden | All |
| 9 | McDonald JC(9) | 2005 | Quantitative descriptive | 1992-2001 | Acute work related respiratory diseases | UK | All |
| 10 | Sirajuddin H(10) | 2001 | Quantitative descriptive | 1997-1998 | Lung diseases, occupational dermatosis, poisoning | Malaysia | All |
| 11 | Moldovan HR(11) | 2017 | Quantitative descriptive | 2013-2014 | Occupational skin diseases | 22 Eastern European countries | All |
| 12 | Baur X(12) | 2005 | Quantitative descriptive | 2003 | Non-malignant occupational airway diseases | Germany | All |
| 13 | Ding Q(13) | 2013 | Qualitative | 2000-2010 | Pneumoconiasis, poisonings | China | All |
| 14 | Machovcova A(14) | 2013 | Quantitative descriptive | 1997-2009 | Occupational skin diseases | Czech Republic | Healthcare industries |
| 15 | Turner S(15) | 2005 | Quantitative descriptive | 2000-2003 | Work-related infectious diseases | UK | All |
| 16 | Walsh L(16) | 2005 | Quantitative descriptive | 2002-2003 | Occupational diseases | UK | Health and social work sectors |
| 17 | Pal TM(17) | 2009 | Quantitative descriptive | 2001-2005 | Occupational skin diseases | Netherlands | All |
| 18 | Samant Y(18) | 2020 | Quantitative descriptive | 2007-2016 | Occupational diseases | Norway | Agriculture |
| 19 | Soo Oh S(19) | 2010 | Quantitative descriptive | 1992-2006 | Occupational asthma | South Korea | All |
| 20 | van der Molen HF(20) | 2012 | Quantitative descriptive | 2009-2013 | Occupational diseases | Netherlands | Economic sector |
| 21 | Burnett CA(21) | 1998 | Quantitative descriptive | 1993 | Occupational dermatitis | United States | Private industries |
| 22 | Aalto-Korte K(22) | 2020 | Quantitative descriptive | 2005-2016 | Occupational skin diseases | Finland | All |
| 23 | Spiewak R(23) | 2003 | Quantitative descriptive | 1991-1999 | Occupational dermatoses | Poland | Agriculture |
| 24 | Karjalainen A(24) | 1997 | Quantitative descriptive | 1960-1995 | Mesothelioma | Finland | All |
| 25 | Ahn Y(25) | 2008 | Quantitative descriptive | 1998-2004 | Occupational infectious diseases | South Korea | Healthcare industries |
| 26 | Cherry NM(26) | 2006 | Quantitative descriptive | 1996-2001 | Work-related stress and mental ill-health | UK | All |
| 27 | Meyer JD(27) | 2002 | Quantitative descriptive | 1997-2000 | Work-related hearing loss | UK | All |
| 28 | Suuronen K(28) | 2007 | Quantitative descriptive | 1992-2001 | Occupational dermatitis and allergic respiratory diseases | Finland | Machine-related industries |
| 29 | Leigh J(29) | 2002 | Quantitative descriptive | 1945-2000 | Mesothelioma | Australia | All |
| 30 | Vainauskas S(30) | 2010 | Quantitative descriptive | 1999-2008 | Occupational Diseases | Lithuania | All |
| 31 | Turner S(31) | 2007 | Quantitative descriptive | 2002-2005 | Occupational skin diseases | UK | All |
| 32 | Szeszenia-Dąbrowska N(32) | 2013 | Quantitative descriptive | 1998-2011 | Occupational diseases | Poland | All |
| 33 | Szeszenia-Dąbrowska N(33) | 2006 | Quantitative descriptive | 2005 | Occupational diseases | Poland | All |
| 34 | Gobba F(34) | 2019 | Quantitative descriptive | 2012-2017 | non-melanoma skin cancers (NMSC) and actinic keratoses (AK) | Italy | Agriculture, Industry and Service sectors |
| 35 | Skov T(35) | 1990 | Quantitative descriptive | 1983-1987 | Occupational cancer | Denmark | All |
| 36 | Paris C(36) | 2012 | Quantitative descriptive | 2001-2009 | Work-related asthma | France | All |
| 37 | Barber CM(37) | 2019 | Quantitative descriptive | 1996-2015 | Occupational hypersensitivity pneumonitis (OHP) | UK | All |
| 38 | Hnizdo E(38) | 2001 | Quantitative descriptive | 1996-1998 | Occupational respiratory diseases | South Africa | Non-mining sector |
| 39 | Schmitt J(39) | 2014 | Quantitative descriptive | 2005-2011 | Occupational skin cancer due to UV radiation | Germany | All |
| 40 | Danø H(40) | 1996 | Quantitative descriptive | 1983-1990 | Occupational cancers (pleural mesothelioma, sinonasal adenocarcinoma, adenocarcinoma of the lung) | Denmark | All |
| 41 | Ameille J(41) | 2003 | Quantitative descriptive | 1996-1999 | Occupational asthma | France | All |
| 42 | Scarselli A(42) | 2010 | Quantitative descriptive | 1995-2008 | Occupational cancers | Italy | All |
| 43 | Nowak-Pasternak J(43) | 2022 | Quantitative descriptive | 2000-2019 | Silicoses | Poland | All |
| 44 | Carøe TK(44) | 2013 | Quantitative descriptive | 2000-2009 | Occupational skin cancer | Denmark | All |
| 45 | van der Molen HF(45) | 2020 | Quantitative descriptive | 2004-2017 | Occupational diseases | Italy | Agriculture |
| 46 | Urban M(46) | 2022 | Quantitative descriptive | 1991-2020 | Asbestoses | Czech Republic | All |
| 47 | Li X(47) | 2022 | Quantitative descriptive | 2006-2020 | Occupational cancer | China | All |
| 48 | Cherry NM(48) | 2001 | Quantitative descriptive | 1997-2000 | Work-related musculoskeletal diseases | UK | All |
| 49 | Świątkowska B(49) | 2017 | Quantitative descriptive | 1970-2015 | Asbestos-related diseases (asbestosis, lung cancer, mesothelioma) | Poland | All |
| 50 | Kee D(50) | 2023 | Quantitative descriptive | 1996-2020 | Work-related musculoskeletal disorders | South Korea | All |
| 51 | Leigh J(51) | 1991 | Quantitative descriptive | 1982-1988 | Malignant mesothelioma | Australia | All |
| 52 | van Kampen V(52) | 2008 | Quantitative descriptive | 1970-2005 | Occupational respiratory diseases | Germany | All |
| 53 | Fenclova Z(53) | 2009 | Quantitative descriptive | 1992-2005 | Occupational hypersensitivity pneumonitis | Czech Republic | All |
| 54 | Jung S(54) | 2012 | Quantitative descriptive | 2001-2010 | Malignant mesothelioma | South Korea | All |
| 55 | Szeszenia-Dąbrowski N(55) | 2016 | Quantitative descriptive | 2000-2014 | Occupational disease | Poland | Agriculture |
| 56 | Cherry N(56) | 2000 | Quantitative descriptive | 1993-1999 | Occupational skin disease | UK | All |
| 57 | Vandenplas O(57) | 2011 | Quantitative descriptive | 1993-2002 | Occupational asthma | Belgium | All |
| 58 | Fagan KM(58) | 2016 | Qualitative | 2011 | Occupational injuries and illnesses | US | Poultry industry |
| 59 | Kwon S(59) | 2015 | Quantitative descriptive | 2004-2009 | Work-related asthma | South Korea | All |
| 60 | Parhar A(60) | 2011 | Quantitative non-RCT | N/A | Occupational asthma | Canada | All |
| 61 | Meyer JD(61) | 2001 | Quantitative descriptive | 1999 | Work-related respiratory diseases | UK | All |
| 62 | Cherry NM(62) | 2000 | Quantitative descriptive | 1996-1999 | Work-related diseases | UK | All |
| 63 | Kim KH(63) | 2010 | Quantitative descriptive | 1996-2009 | Work-related musculoskeletal disorders | South Korea | All |
| 64 | Money A(64) | 2011 | Quantitative descriptive | 1998-2006 | Work-related audiological disease | UK | All |
| 65 | Karjalainen A(65) | 2000 | Quantitative descriptive | 1989-1995 | Occupational asthma | Finland | All |
| 66 | Dulon M(66) | 2011 | Quantitative descriptive | 1998-2007 | Occupational airway diseases | German | Hair craft and health service |
| 67 | Aalto-Korte K(22) | 2019 | Quantitative descriptive | 2005-2016 | Occupational skin diseases | Finland | All |
| 68 | Alfonso JH(67) | 2015 | Quantitative descriptive | 2000-2013 | Work-related skin diseases | Norway | All |
| 69 | Oksa P(68) | 2019 | Quantitative descriptive | 1975-2013 | Occupational diseases | Finland | All |
| 70 | Carder M(69) | 2009 | Quantitative descriptive | 2002-2005 | Work-related mental ill health | UK | All |
| 71 | Meyer JD(70) | 2000 | Quantitative descriptive | 1996-1999 | Occupational contact dermatitis | UK | All |
| 72 | Latza U(71) | 2005 | Quantitative descriptive | 2003 | Occupational obstructive airway diseases | German | Industrial sector |
| 73 | Hussey L(72) | 2013 | Quantitative descriptive | 2006-2009 | Work-related ill-health | Great Britain | All |
| 74 | Zhou AY(73) | 2017 | Quantitative descriptive | 2001-2014 | Work-related ill-health and work-related mental ill health | Great Britain | Healthcare |
| 75 | Arnaud S(74) | 2010 | Quantitative non-RCT | November 2006 - February 2007 | Occupational diseases | France | N/A |
| 76 | Shum KW(75) | 2003 | Quantitative descriptive | 1993-1999 | Occupational contact dermatitis | UK | All |
| 77 | Halioua B(76) | 2012 | Quantitative descriptive | 2004-2007 | Occupational contact dermatitis | France | All |
| 78 | Carder M(77) | 2013 | Quantitative descriptive | 1996-2009 | Work-related mental ill-health and musculoskeletal disorders | UK | All |
| 79 | Shin S(78) | 2022 | Quantitative descriptive | 2001-2020 | Occupational infectious disease | South Korea | All |
| 80 | Cheng Y(79) | 2022 | Qualitative | July 2014 to March 2017 | Asbestos-related diseases | Taiwan | Shipbreaking, ship repairing, asbestos spraying in construction, and manufacturing and processing asbestos-containing products. |
| 81 | Hussey L(80) | 2008 | Quantitative descriptive | 2006-2007 | Work-related ill health | UK | All |
| 82 | Shih P(81) | 2023 | Quantitative descriptive | 2008-2021 | Occupational diseases | Taiwan | All |
| 83 | Malsam R(82) | 2021 | Quantitative descriptive | 2006-2019 | Occupational infectious diseases | German | Healthcare industries |
| 84 | Downs JW(83) | 2021 | Quantitative descriptive | 2008-2018 | Occupational poisonings | US | All |
| 85 | Carder M(84) | 2017 | Quantitative descriptive | 1996-2014 | Work-related, long-latency respiratory diseases | Great Britain | All |
| 86 | Cha E-W(85) | 2022 | Quantitative descriptive | 2020 | Work-related musculoskeletal disorders | South Korea | All |
| 87 | Carder M(86) | 2014 | Quantitative descriptive | 2005 & 2008 | Occupational disease | Great Britain | All |
| 88 | Carder M(87) | 2019 | Quantitative descriptive | 1989-2017 | Occupational and work-related respiratory disease attributed to cleaning products | UK | All |
| 89 | Hussey L(88) | 2010 | Quantitative descriptive | 2006-2007 | Work-related ill health | UK | All |
| 90 | Kourouklis GN(89) | 2009 | Quantitative descriptive | 2003-2007 | Occupational diseases | Greece | All |
| 91 | Barber CM(37) | 2018 | Quantitative descriptive | 1996-2017 | Silicosis | UK | All |
| 92 | Chen Y(90) | 2005 | Quantitative descriptive | 2004 | Occupational diseases | UK | Healthcare industries |
| 93 | Miedema HS(91) | 2013 | Quantitative descriptive | 2004-2011 | Low back pain related occupational diseases | Netherlands | All |
| 94 | Chen Y(92) | 2005 | Quantitative descriptive | 2002-2003 | Work-related musculoskeletal disorders | Great Britain | All |
| 95 | Stocks SJ(93) | 2015 | Quantitative descriptive | 2000-2012 | occupational asthma, contact dermatitis, noise-induced hearing loss, carpal tunnel syndrome and upper limb musculoskeletal disorders | 10 European countries: Belgium, the Czech Republic, Finland, France, Italy, the Netherlands, Norway, Spain, Switzerland and the UK | All |
| 96 | Moreno-Torres L(94) | 2018 | Quantitative descriptive | 2000-2015 | Occupational illnesses | Mexico | All |
| 97 | Alaguney ME(95) | 2020 | Quantitative descriptive | 2015 | Occupational diseases | Turkey | All |
| 98 | Scarselli A(96) | 2009 | Quantitative descriptive | 1994-2006 | Occupational cancer | Italy | Industrial sector |
| 99 | Grignoux J(97) | 2019 | Quantitative descriptive | 2001-2016 | Work-related laryngeal cancer | France | All |
| 100 | Garnett J(98) | 2020 | Quantitative descriptive | 2012-2019 | Occupational tuberculosis | South Africa | Healthcare industries |
| 101 | Kuijer P(99) | 2014 | Quantitative descriptive | 2005-2012 | Non-specific low back pain | Netherlands | All |
| 102 | Stocks SJ(100) | 2011 | Quantitative descriptive | 2002-2008 | Work-related ill health | UK | Construction industry |
| 103 | Carøe TK(44) | 2013 | Quantitative descriptive | 2010 | Occupational contact dermatitis | Denmark | All |
| 104 | Kim E(101) | 2021 | Quantitative descriptive | 1995-2017 | Malignant mesothelioma | South Korea | All |
| 105 | Money A(102) | 2015 | Quantitative descriptive | 2005-2012 | Work-related ill health | Republic of Ireland, Northern Ireland, Great Britain | All |
| 106 | Luckhaupt S(103) | 2010 | Quantitative descriptive | 1988 | Work-related chronic conditions | US | All |
| 107 | Chen Y(104) | 2005 | Quantitative descriptive | 2002-2003 | Work-related ill health | Scotland | All |
| 108 | Turner S(105) | 2015 | Quantitative descriptive | 1996-2012 | Work-related skin neoplasia | UK | All |
| 109 | Karttunen J(106) | 2013 | Quantitative descriptive | 1982-2008 | Occupational diseases | Finland | Agriculture |
| 110 | Plombom G(107) | 2015 | Quantitative descriptive | 2007-2012 | Occupational dermatitis | Brazil | All |
| 111 | Morken T(108) | 2007 | Quantitative descriptive | 1992-2003 | Work-related musculoskeletal disorders | Norway | Offshore petroleum industry |
| 112 | Stocks SJ(109) | 2010 | Quantitative descriptive | 2002-2008 | Work-related ill health | UK | Agriculture |
| 113 | Bensefa-Colas L(110) | 2015 | Quantitative descriptive | 2001-2010 | Occupational contact urticaria | France | All |
| 114 | Kersten JF(111) | 2020 | Quantitative descriptive | 2002-2017 | Occupational tuberculosis | German | Healthcare industries |
| 115 | Binazzi A(112) | 2021 | Quantitative descriptive | 1993-2018 | Malignant mesothelioma | Italy | Construction industry |
| 116 | Zhou AY(113) | 2017 | Quantitative descriptive | 2006-2009 | Work-related mental ill-health | Great Britain | Healthcare industries |
| 117 | Bensefa-Colas L(114) | 2014 | Quantitative descriptive | 2001-2010 | Occupational allergic contact dermatitis | France | All |
| 118 | Lysdal SH(115) | 2011 | Quantitative descriptive | 2009 | Hand eczema | Denmark | Service industry |
| 119 | McNamee R(116) | 2007 | Quantitative descriptive | 1996-2005 | Work-related skin and respiratory diseases | UK | All |
| 120 | Nienhaus A(117) | 2012 | Quantitative descriptive | 2005-2009 | Infectious diseases | German | Healthcare industries |
| 121 | Kanerva L(118) | 2000 | Quantitative descriptive | 1991-1997 | Occupational allergic contact dermatitis | Finland | All |
| 122 | Medeni I(119) | 2024 | Quantitative descriptive | 2018-2022 | Occupational diseases | Turkey | All |
| 123 | Fishwick D(120) | 2023 | Quantitative descriptive | 1998-2018 | Irritant asthma | UK | All |
| 124 | Karabağ I(121) | 2023 | Qualitative | 2021 | Occupational diseases | Turkey | All |
| 125 | Samant Y(122) | 2023 | Quantitative descriptive | 2022-2022 | COVID-19 | Norway | All |
| 126 | Su TY(123) | 2023 | Quantitative descriptive | 2004-2020 | Silicosis | Taiwan | All |
| 127 | Iskandar IYK(124) | 2024 | Quantitative descriptive | 1996-2019 | Occupational Diseases | UK | All |
|  |  |  |  |  |  |  |  |

1. Murphy PL, Volinn E. Is occupational low back pain on the rise? Spine (Phila Pa 1976). 1999;24(7):691-7.

2. Kraut A. Estimates of the extent of morbidity and mortality due to occupational diseases in Canada. Am J Ind Med. 1994;25(2):267-78.

3. Nordman H KA, Kesinen H. Incidence of Occupational Asthma: A Comparison by Reporting Systems. American Journal of Industrial Medicine Supplement. 1999;1:130-3.

4. Pelclova D FZ, Urban P. Asbestos Exposure, Legislation and Diseases in the Czech Republic. Cent Eur J Public Health. 2007;15(3):99-102.

5. Ross DJ KH, McDonald JC. SWORD '97: Surveillance of work-related and occupational respiratory disease in the UK. OccupMed. 1998;48(8):481-5.

6. McDonald JC, Beck MH, Chen Y, Cherry NM. Incidence by occupation and industry of work-related skin diseases in the United Kingdom, 1996–2001. Occupational Medicine. 2006;56(6):398-405.

7. Chen JKC, Zorigt D. Managing occupational health and safety in the mining industry. Journal of Business Research. 2013;66(11):2321-31.

8. Torén K. Self reported rate of occupational asthma in Sweden 1990-2. Occupational and Environmental Medicine. 1996;53(11):757.

9. McDonald JC CY, Zekveld C, Cherry NM. Incidence by occupation and industry of acute work related respiratory diseases in the UK, 1992-2001. Occup Environ Med. 2005;62:836-42.

10. Sirajuddin H, Roslinah A, Rampal KG, Kuppusamy I, Rohna R, Aziz M, et al. Notification of occupational and work-related diseases and poisonings in Malaysia, 1997-1998. Med J Malaysia. 2001;56(1):25-31.

11. Moldovan HR, Voidazan ST, John SM, Weinert P, Moldovan G, Vlasiu MA, et al. The Eastern European experience on occupational skin diseases. Make underreporting an issue? J Eur Acad Dermatol Venereol. 2017;31 Suppl 4:5-11.

12. Baur X, Latza U. Non-malignant occupational respiratory diseases in Germany in comparison with those of other countries. Int Arch Occup Environ Health. 2005;78(7):593-602.

13. Ding Q SL, Hansson SO. Occupational diseases in the People’s Republic of China between 2000 and 2010. American Journal of Industrial Medicine. 2013;56(12):1423-32.

14. Machovcová A, Fenclová Z, Pelclová D. Occupational skin diseases in Czech healthcare workers from 1997 to 2009. Int Arch Occup Environ Health. 2013;86(3):289-94.

15. Turner S, Lines S, Chen Y, Hussey L, Agius R. Work-related infectious disease reported to the Occupational Disease Intelligence Network and The Health and Occupation Reporting network in the UK (2000-2003). Occup Med (Lond). 2005;55(4):275-81.

16. Walsh L, Turner S, Lines S, Hussey L, Chen Y, Agius R. The incidence of work-related illness in the UK health and social work sector: The Health and Occupation Reporting network 2002-2003. Occup Med (Lond). 2005;55(4):262-7.

17. Pal TM, de Wilde NS, van Beurden MM, Coenraads PJ, Bruynzeel DP. Notification of occupational skin diseases by dermatologists in The Netherlands. Occup Med (Lond). 2009;59(1):38-43.

18. Samant Y, Aas O, Ekle R, Gravseth HM, Strømholm T. Physician Notified Work-Related Diseases Among Farmers in Norway: Data from 2007 - 2016. J Agromedicine. 2020;25(2):201-9.

19. Oh SS, Kim KS. Occupational asthma in Korea. J Korean Med Sci. 2010;25(Suppl):S20-5.

20. van der Molen HF, Kuijer PP, Smits PB, Schop A, Moeijes F, Spreeuwers D, et al. Annual incidence of occupational diseases in economic sectors in The Netherlands. Occup Environ Med. 2012;69(7):519-21.

21. Burnett CA, Lushniak BD, McCarthy W, Kaufman J. Occupational dermatitis causing days away from work in U.S. private industry, 1993. Am J Ind Med. 1998;34(6):568-73.

22. Aalto-Korte K, Koskela K, Pesonen M. 12-year data on skin diseases in the Finnish Register of Occupational Diseases II: Risk occupations with special reference to allergic contact dermatitis. Contact Dermatitis. 2020;82(6):343-9.

23. Spiewak R. Occupational dermatoses among Polish private farmers, 1991-1999. Am J Ind Med. 2003;43(6):647-55.

24. Karjalainen A, Pukkala E, Mattson K, Tammilehto L, Vainio H. Trends in mesothelioma incidence and occupational mesotheliomas in Finland in 1960-1995. Scand J Work Environ Health. 1997;23(4):266-70.

25. Ahn YS, Lim HS. Occupational infectious diseases among Korean health care workers compensated with Industrial Accident Compensation Insurance from 1998 to 2004. Ind Health. 2008;46(5):448-54.

26. Cherry NM, Chen Y, McDonald JC. Reported incidence and precipitating factors of work-related stress and mental ill-health in the United Kingdom (1996-2001). Occup Med (Lond). 2006;56(6):414-21.

27. Meyer JD, Chen Y, McDonald JC, Cherry NM. Surveillance for work-related hearing loss in the UK: OSSA and OPRA 1997-2000. Occup Med (Lond). 2002;52(2):75-9.

28. Suuronen K, Aalto-Korte K, Piipari R, Tuomi T, Jolanki R. Occupational dermatitis and allergic respiratory diseases in Finnish metalworking machinists. Occup Med (Lond). 2007;57(4):277-83.

29. Leigh J, Davidson P, Hendrie L, Berry D. Malignant mesothelioma in Australia, 1945-2000. Am J Ind Med. 2002;41(3):188-201.

30. Vainauskas S, Venckienė R, Krisiulevičienė D, Chomentauskas A, Januškevičius V, Vasilavičius P. Trends in the incidence of occupational diseases in Lithuania between 1999 and 2008. Int J Occup Med Environ Health. 2010;23(4):317-22.

31. Turner S, Carder M, van Tongeren M, McNamee R, Lines S, Hussey L, et al. The incidence of occupational skin disease as reported to The Health and Occupation Reporting (THOR) network between 2002 and 2005. Br J Dermatol. 2007;157(4):713-22.

32. Szeszenia-Dąbrowska N, Wilczyńska U. Occupational diseases in Poland--an overview of current trends. Int J Occup Med Environ Health. 2013;26(3):457-70.

33. Szeszenia-Dabrowska N, Wilczyńska U. Occupational diseases in the period of socioeconomic transition in Poland. Int J Occup Med Environ Health. 2006;19(2):99-106.

34. Gobba F, Modenese A, John SM. Skin cancer in outdoor workers exposed to solar radiation: a largely underreported occupational disease in Italy. J Eur Acad Dermatol Venereol. 2019;33(11):2068-74.

35. Skov T, Mikkelsen S, Svane O, Lynge E. Reporting of occupational cancer in Denmark. Scand J Work Environ Health. 1990;16(6):401-5.

36. Paris C, Ngatchou-Wandji J, Luc A, McNamee R, Bensefa-Colas L, Larabi L, et al. Work-related asthma in France: recent trends for the period 2001-2009. Occup Environ Med. 2012;69(6):391-7.

37. Barber CM, Fishwick D, Carder M, van Tongeren M. Epidemiology of silicosis: reports from the SWORD scheme in the UK from 1996 to 2017. Occup Environ Med. 2019;76(1):17-21.

38. Hnizdo E, Esterhuizen TM, Rees D, Lalloo UG. Occupational asthma as identified by the Surveillance of Work-related and Occupational Respiratory Diseases programme in South Africa. Clin Exp Allergy. 2001;31(1):32-9.

39. Schmitt J, Diepgen TL. Occupational skin cancer due to UV-irradiation--Analyses of notified cases as "virtually-certain" occupational disease in Germany between 2005 and 2011. J Dtsch Dermatol Ges. 2014;12(6):491-7.

40. Danø H, Skov T, Lynge E. Underreporting of occupational cancers in Denmark. Scand J Work Environ Health. 1996;22(1):55-7.

41. Ameille J, Pauli G, Calastreng-Crinquand A, Vervloët D, Iwatsubo Y, Popin E, et al. Reported incidence of occupational asthma in France, 1996-99: the ONAP programme. Occup Environ Med. 2003;60(2):136-41.

42. Scarselli A, Massari S, Binazzi A, Di Marzio D, Scano P, Marinaccio A, et al. Italian National Register of Occupational Cancers: data system and findings. J Occup Environ Med. 2010;52(3):346-53.

43. Nowak-Pasternak J, Lipińska-Ojrzanowska A, Świątkowska B. Epidemiology of silicosis reported to the central register of occupational diseases over last 20 years in Poland. Int J Occup Med Environ Health. 2022;35(5):561-70.

44. Carøe TK, Ebbehøj NE, Wulf HC, Agner T. Occupational skin cancer may be underreported. Dan Med J. 2013;60(5):A4624.

45. van der Molen HF, Marsili C, Vitali A, Colosio C. Trends in occupational diseases in the Italian agricultural sector, 2004-2017. Occup Environ Med. 2020;77(5):340-3.

46. Urban M, Pelclová D, Urban P, Vít M, Urban P, Fenclová Z. Asbestos danger in central Europe is not yet over - the situation in the Czech Republic. Cent Eur J Public Health. 2022;30(2):67-73.

47. Li X, Wang D, Liu A, Hu W, Sun X. Epidemiological Characteristics of Occupational Cancers Reported - China, 2006-2020. China CDC Wkly. 2022;4(17):370-3.

48. Cherry NM, Meyer JD, Chen Y, Holt DL, McDonald JC. The reported incidence of work-related musculoskeletal disease in the UK: MOSS 1997-2000. Occup Med (Lond). 2001;51(7):450-5.

49. Swiatkowska B, Szeszenia-Dabrowska N. Long-term epidemiological observation of asbestos-related diseases in Poland, 1970-2015. Occup Med (Lond). 2017;67(3):182-7.

50. Kee D. Characteristics of Work-Related Musculoskeletal Disorders in Korea. Int J Environ Res Public Health. 2023;20(2).

51. Leigh J, Corvalán CF, Grimwood A, Berry G, Ferguson DA, Thompson R. The incidence of malignant mesothelioma in Australia 1982-1988. Am J Ind Med. 1991;20(5):643-55.

52. van Kampen V, Merget R, Butz M, Taeger D, Brüning T. Trends in suspected and recognized occupational respiratory diseases in Germany between 1970 and 2005. Am J Ind Med. 2008;51(7):492-502.

53. Fenclová Z, Pelclová D, Urban P, Navrátil T, Klusácková P, Lebedová J. Occupational hypersensitivity pneumonitis reported to the Czech National Registry Of Occupational Diseases in the period 1992-2005. Ind Health. 2009;47(4):443-8.

54. Jung SH, Kim HR, Koh SB, Yong SJ, Chung MJ, Lee CH, et al. A decade of malignant mesothelioma surveillance in Korea. Am J Ind Med. 2012;55(10):869-75.

55. Szeszenia-Dąbrowska N, Świątkowska B, Wilczyńska U. Occupational diseases among farmers in Poland. Med Pr. 2016;67(2):163-71.

56. Cherry N, Meyer JD, Adisesh A, Brooke R, Owen-Smith V, Swales C, et al. Surveillance of occupational skin disease: EPIDERM and OPRA. Br J Dermatol. 2000;142(6):1128-34.

57. Vandenplas O, Lantin AC, D'Alpaos V, Larbanois A, Hoet P, Vandeweerdt M, et al. Time trends in occupational asthma in Belgium. Respir Med. 2011;105(9):1364-72.

58. Fagan KM, Hodgson MJ. Under-recording of work-related injuries and illnesses: An OSHA priority. J Safety Res. 2017;60:79-83.

59. Kwon SC, Song J, Kim YK, Calvert GM. Work-Related Asthma in Korea - Findings from the Korea Work-Related Asthma Surveillance (KOWAS) program, 2004-2009. Allergy Asthma Immunol Res. 2015;7(1):51-9.

60. Parhar A, Lemiere C, Beach JR. Barriers to the recognition and reporting of occupational asthma by Canadian pulmonologists. Can Respir J. 2011;18(2):90-6.

61. Meyer JD, Holt DL, Chen Y, Cherry NM, McDonald JC. SWORD '99: surveillance of work-related and occupational respiratory disease in the UK. Occup Med (Lond). 2001;51(3):204-8.

62. Cherry NM, Meyer JD, Holt DL, Chen Y, McDonald JC. Surveillance of work-related diseases by occupational physicians in the UK: OPRA 1996-1999. Occup Med (Lond). 2000;50(7):496-503.

63. Kim KH, Kim KS, Kim DS, Jang SJ, Hong KH, Yoo SW. Characteristics of work-related musculoskeletal disorders in Korea and their work-relatedness evaluation. J Korean Med Sci. 2010;25(Suppl):S77-86.

64. Money A, Carder M, Turner S, Hussey L, Agius R. Surveillance for work-related audiological disease in the UK: 1998–2006. Occupational Medicine. 2011;61(4):226-33.

65. Karjalainen A, Kurppa K, Virtanen S, Keskinen H, Nordman H. Incidence of occupational asthma by occupation and industry in Finland. Am J Ind Med. 2000;37(5):451-8.

66. Dulon M, Peters C, Wendeler D, Nienhaus A. Trends in occupational airway diseases in German hairdressers: frequency and causes. Am J Ind Med. 2011;54(6):486-93.

67. Alfonso JH, Løvseth EK, Samant Y, Holm J. Work-related skin diseases in Norway may be underreported: data from 2000 to 2013. Contact Dermatitis. 2015;72(6):409-12.

68. Oksa P, Sauni R, Talola N, Virtanen S, Nevalainen J, Saalo A, et al. Trends in occupational diseases in Finland, 1975-2013: a register study. BMJ Open. 2019;9(4):e024040.

69. Carder M, Turner S, McNamee R, Agius R. Work-related mental ill-health and 'stress' in the UK (2002-05). Occup Med (Lond). 2009;59(8):539-44.

70. Meyer JD, Chen Y, Holt DL, Beck MH, Cherry NM. Occupational contact dermatitis in the UK: a surveillance report from EPIDERM and OPRA. Occup Med (Lond). 2000;50(4):265-73.

71. Latza U, Baur X. Occupational obstructive airway diseases in Germany: Frequency and causes in an international comparison. Am J Ind Med. 2005;48(2):144-52.

72. Hussey L, Carder M, Money A, Turner S, Agius R. Comparison of work-related ill-health data from different GB sources. Occup Med (Lond). 2013;63(1):30-7.

73. Zhou AY, Carder M, Gittins M, Agius R. Work-related ill health in doctors working in Great Britain: incidence rates and trends. Br J Psychiatry. 2017;211(5):310-5.

74. Arnaud S, Cabut S, Viau A, Souville M, Verger P. Different reporting patterns for occupational diseases among physicians: a study of French general practitioners, pulmonologists and rheumatologists. Int Arch Occup Environ Health. 2010;83(3):251-8.

75. Shum KW, Meyer JD, Chen Y, Cherry N, Gawkrodger DJ. Occupational contact dermatitis to nickel: experience of the British dermatologists (EPIDERM) and occupational physicians (OPRA) surveillance schemes. Occup Environ Med. 2003;60(12):954-7.

76. Halioua B, Bensefa-Colas L, Bouquiaux B, Crépy MN, Assier H, Billon S, et al. Occupational contact dermatitis in 10,582 French patients reported between 2004 and 2007: a descriptive study. Dermatology. 2012;225(4):354-63.

77. Carder M, McNamee R, Turner S, Hodgson JT, Holland F, Agius RM. Time trends in the incidence of work-related mental ill-health and musculoskeletal disorders in the UK. Occup Environ Med. 2013;70(5):317-24.

78. Shin S, Yoon WS, Byeon SH. Trends in Occupational Infectious Diseases in South Korea and Classification of Industries According to the Risk of Biological Hazards Using K-Means Clustering. Int J Environ Res Public Health. 2022;19(19).

79. Cheng Y, Huang YL, Lee LJ. Explaining the Invisibility of Asbestos-Related Diseases in the Taiwan Workers' Compensation System. New Solut. 2022;32(2):106-18.

80. Hussey L, Turner S, Thorley K, McNamee R, Agius R. Work-related ill health in general practice, as reported to a UK-wide surveillance scheme. Br J Gen Pract. 2008;58(554):637-40.

81. Shih P, Chu PC, Huang CC, Guo YL, Chen PC, Su TC. Hospital Occupational Health Service Network and Reporting Systems in Taiwan From 2008 to 2021. J Occup Environ Med. 2023;65(2):e43-e50.

82. Malsam R, Nienhaus A. Occupational Infections among Dental Health Workers in Germany-14-Year Time Trends. Int J Environ Res Public Health. 2021;18(19).

83. Downs JW, Wills BK, Cumpston KL, Rose SR. Descriptive epidemiology of clinically significant occupational poisonings, United States, 2008-2018. Clin Toxicol (Phila). 2021;59(12):1259-63.

84. Carder M, Darnton A, Gittins M, Stocks SJ, Ross D, Barber CM, et al. Chest physician-reported, work-related, long-latency respiratory disease in Great Britain. Eur Respir J. 2017;50(6).

85. Cha EW, Jung SM, Lee IH, Kim DH, Choi EH, Kim IA, et al. Approval status and characteristics of work-related musculoskeletal disorders among Korean workers in 2020. Ann Occup Environ Med. 2022;34:e31.

86. Carder M, Money A, Turner S, Agius R. Workforce coverage by GB occupational physicians and disease incidence rates. Occup Med (Lond). 2014;64(4):271-8.

87. Carder M, Seed MJ, Money A, Agius RM, van Tongeren M. Occupational and work-related respiratory disease attributed to cleaning products. Occup Environ Med. 2019;76(8):530-6.

88. Hussey L, Turner S, Thorley K, McNamee R, Agius R. Comparison of work-related ill health reporting by occupational physicians and general practitioners. Occup Med (Lond). 2010;60(4):294-300.

89. Kourouklis GN. Disability from occupational diseases in Greece. Occup Med (Lond). 2009;59(7):515-7.

90. Chen Y, Agius R, McNamee R, Turner S, Taylor S, Fulluck L, et al. Physicians' beliefs in the assessment of work attribution when reporting musculoskeletal disorders. Occup Med (Lond). 2005;55(4):298-307.

91. Miedema HS, van der Molen HF, Kuijer PP, Koes BW, Burdorf A. Incidence of low back pain related occupational diseases in the Netherlands. Eur J Pain. 2014;18(6):873-82.

92. Chen Y, Turner S, Hussey L, Agius R. A study of work-related musculoskeletal case reports to The Health and Occupation Reporting network (THOR) from 2002 to 2003. Occup Med (Lond). 2005;55(4):268-74.

93. Stocks SJ, McNamee R, van der Molen HF, Paris C, Urban P, Campo G, et al. Trends in incidence of occupational asthma, contact dermatitis, noise-induced hearing loss, carpal tunnel syndrome and upper limb musculoskeletal disorders in European countries from 2000 to 2012. Occup Environ Med. 2015;72(4):294-303.

94. Moreno-Torres LA, Ventura-Alfaro CE. Underreporting trends of occupational illnesses in Mexico. J Occup Health. 2018;60(1):85-8.

95. Alaguney ME YA, Demir AU, Ergor OA. Physicians’ opinions about the causes of underreporting of occupational diseases. Archives of Environmental & Occupational Health. 2020;75(3):165-76.

96. Scarselli A, Scano P, Marinaccio A, Iavicoli S. Occupational cancer in Italy: Evaluating the extent of compensated cases in the period 1994–2006. American Journal of Industrial Medicine. 2009;52(11):859-67.

97. Grignoux J, Durand-Moreau Q, Vongmany N, Brunel S, Dewitte JD. Work-related laryngeal cancer: Trends in France from 2001 to 2016. Eur Ann Otorhinolaryngol Head Neck Dis. 2019;136(1):7-12.

98. Garnett J, Jones D, Chin G, Spiegel JM, Yassi A, Naicker N. Occupational Tuberculosis Among Laboratory Workers in South Africa: Applying a Surveillance System to Strengthen Prevention and Control. Int J Environ Res Public Health. 2020;17(5).

99. Kuijer PP, van der Molen HF, Schop A, Moeijes F, Frings-Dresen MH, Hulshof CT. Annual incidence of non-specific low back pain as an occupational disease attributed to whole-body vibration according to the National Dutch Register 2005-2012. Ergonomics. 2015;58(7):1232-8.

100. Stocks SJ, Turner S, McNamee R, Carder M, Hussey L, Agius RM. Occupation and work-related ill-health in UK construction workers. Occup Med (Lond). 2011;61(6):407-15.

101. Kim EA. Standardized Incidence Ratio and Standardized Mortality Ratio of Malignant Mesothelioma in a Worker Cohort Using Employment Insurance Database in Korea. Int J Environ Res Public Health. 2021;18(20).

102. Money A, Carder M, Noone P, Bourke J, Hayes J, Turner S, et al. Work-related ill-health: Republic of Ireland, Northern Ireland, Great Britain 2005-2012. Occup Med (Lond). 2015;65(1):15-21.

103. Luckhaupt SE, Calvert GM. Work-relatedness of selected chronic medical conditions and workers' compensation utilization: National health interview survey occupational health supplement data. Am J Ind Med. 2010;53(12):1252-63.

104. Chen Y, Turner S, McNamee R, Ramsay CN, Agius RM. The reported incidence of work-related ill-health in Scotland (2002-2003). Occup Med (Lond). 2005;55(4):252-61.

105. Turner S, Forman SD, McNamee R, Wilkinson SM, Agius R. Investigating work-related neoplasia associated with solar radiation. Occup Med (Lond). 2015;65(1):22-8.

106. Karttunen JP, Rautiainen RH. Distribution and characteristics of occupational injuries and diseases among farmers: a retrospective analysis of workers' compensation claims. Am J Ind Med. 2013;56(8):856-69.

107. Plombom GY, Oliveira MS, Tabushi FL, Kassem AJ, Purim KS, Nisihara RM. Epidemiological analysis of occupational dermatitis notified in Brazil in the period 2007 to 2012. An Bras Dermatol. 2016;91(6):732-6.

108. Morken T, Mehlum IS, Moen BE. Work-related musculoskeletal disorders in Norway's offshore petroleum industry. Occup Med (Lond). 2007;57(2):112-7.

109. Stocks SJ, Turner S, Carder M, Hussey L, McNamee R, Agius RM. Medically reported work-related ill-health in the UK agricultural sector. Occup Med (Lond). 2010;60(5):340-7.

110. Bensefa-Colas L, Telle-Lamberton M, Faye S, Bourrain JL, Crépy MN, Lasfargues G, et al. Occupational contact urticaria: lessons from the French National Network for Occupational Disease Vigilance and Prevention (RNV3P). Br J Dermatol. 2015;173(6):1453-61.

111. Kersten JF, Nienhaus A, Schneider S, Schablon A. Tuberculosis among Health Workers-A Secondary Data Analysis of German Social Accident Insurance Data from 2002-2017. Int J Environ Res Public Health. 2020;17(5).

112. Binazzi A, Di Marzio D, Verardo M, Migliore E, Benfatto L, Malacarne D, et al. Asbestos Exposure and Malignant Mesothelioma in Construction Workers-Epidemiological Remarks by the Italian National Mesothelioma Registry (ReNaM). Int J Environ Res Public Health. 2021;19(1).

113. Zhou AY, Carder M, Hussey L, Gittins M, Agius R. Differential reporting of work-related mental ill-health in doctors. Occup Med (Lond). 2017;67(7):522-7.

114. Bensefa-Colas L, Telle-Lamberton M, Paris C, Faye S, Stocks SJ, Luc A, et al. Occupational allergic contact dermatitis and major allergens in France: temporal trends for the period 2001-2010. Br J Dermatol. 2014;171(6):1375-85.

115. Lysdal SH, Søsted H, Johansen JD. Do hairdressers in Denmark have their hand eczema reported as an occupational disease? Results from a register-based questionnaire study. Contact Dermatitis. 2012;66(2):72-8.

116. McNamee R, Carder M, Chen Y, Agius R. Measurement of trends in incidence of work-related skin and respiratory diseases, UK 1996-2005. Occup Environ Med. 2008;65(12):808-14.

117. Nienhaus A, Kesavachandran C, Wendeler D, Haamann F, Dulon M. Infectious diseases in healthcare workers - an analysis of the standardised data set of a German compensation board. J Occup Med Toxicol. 2012;7(1):8.

118. Kanerva L, Jolanki R, Estlander T, Alanko K, Savela A. Incidence rates of occupational allergic contact dermatitis caused by metals. Am J Contact Dermat. 2000;11(3):155-60.

119. Medeni İ, Alagüney ME, Medeni V. Medical and legal diagnoses comparison of the occupational diseases: A nationwide study in Turkey. J Eval Clin Pract. 2024;30(7):1449-56.

120. Fishwick D, Carder M, Iskandar I, Fishwick BC, van Tongeren M. Irritant asthma and work: cases from the UK SWORD reporting scheme from 1999 to 2018. Occup Environ Med. 2023;80(10):553-7.

121. Karabağ İ, Alagüney ME, Şahan C, Yıldız AN. How difficult is it to diagnose and report an occupational disease in a developing country? A modified delphi study. Acta Medica. 2023;54(4):347-56.

122. Samant Y, Støver M, Haarberg IS, Lohmann-Lafrenz S, Strømholm T. Underreporting of Work-Related COVID-19 Cases in Norway. New Solut. 2023;33(2-3):149-53.

123. Su TY, Lee LJ, Chen JM, Chung SH, Wu WT. Analyzing the incidence of silicosis across various industries in Taiwan: a study of occupational disease surveillance by linking national-based workers' and medicoadministrative databases. Public Health. 2023;225:110-9.

124. Iskandar IYK, Gawkrodger DJ, Byrne L, Gittins M, Carder M, Fishwick D, et al. Trends in work-related respiratory diseases attributed to nickel, chromium and cobalt in the UK: descriptive findings from The Health and Occupation Research (THOR) network 1996-2019. Occup Environ Med. 2024;81(4):220-4.
