# Supplementary material for: Global reporting and underreporting of occupational diseases: A systematic review
Source: PLoS One. 2026 Mar 26;21(3):e0345318. doi: 10.1371/journal.pone.0345318 (PMC13020801; doi:10.1371/journal.pone.0345318)
Supplement: S5 Fig — (DOCX) [file pone.0345318.s005.docx]

Category 1: Objective

Category 2: occupational diseases

(all type versus specific)

Category 3: industrial sector

(general versus specific)

Note: Category 1: studies were classified based on the study objectives, i.e., objective 1) to identify the reporting pattern of occupational diseases; objective 2) to understand the factors affecting underreporting of occupational diseases. Category 2: studies were classified based on the type of occupational diseases, 1) studies reported all occupational diseases, 2) studies reported only specific occupational diseases. OD = occupational disease. Category 3: studies were classified based on the industrial sector, 1) all industrial sectors, 2) specific industrial sector.

**Fig 2. Number of studies in each category based on study objectives, type of occupational diseases and industrial sector**
