# Supplementary material for: Global reporting and underreporting of occupational diseases: A systematic review
Source: PLoS One. 2026 Mar 26;21(3):e0345318. doi: 10.1371/journal.pone.0345318 (PMC13020801; doi:10.1371/journal.pone.0345318)
Supplement: S6 Table — (DOCX) [file pone.0345318.s006.docx]

**Table 3. Number of cases and incidence rate of occupational diseases**

| Author, year | Reported period (year) | Country | Annual average n of cases (min-max) | IR per 100,000 employees |
| --- | --- | --- | --- | --- |
| *All type of occupational diseases in all sectors* | | | | |
| *Data type: physician’s report* | | | | |
| Cherry, 2000(1) | 1996-1999 | UK | 10,941 | 42 |
| Chen, 2005(a)(2) | 2002-2003 | Scotland | 2,022 | 86 |
| Chen, 2005(b)(3) | 2002-2003 | UK, except Scotland | 21,120 | 84 |
| Hussey, 2008(4) | 2006-2007 | UK | 1,436 | NA |
| Hussey, 2010(a)(5) | 2006-2007 | UK | 1,436 | NA |
| Hussey, 2010(b)(5) | 2006-2007 | UK | 1,680 | NA |
| van der Molen, 2012(6) | 2009-2013 | Netherlands | 1,782 | 346 (330-362) |
| Hussey, 2013(a)(7) | 2006-2009 | UK | 1,171 | 1,387 |
| Hussey, 2013(b)(7) | 2006-2009 | UK | 7,759 | 131 |
| Carder, 2014(8) | 2005-2007 | GB | 8,349 | 301 |
|  | 2008-2010 | GB | 5,566 | 336 |
| Money, 2015(9) | 2007-2012 | GB | 4,901 | NA |
|  |  | NI | 557 | NA |
|  |  | ROI | 161 | NA |
| Medeni, 2024(10) | 2018-2022 | Turkey | 4,506 (3,231-5,952) | 15.78 (11.14-21.20) |
| *Data type: National Registry* | | | | |
| Shih, 2023(11) | 2008-2021 | Taiwan | 2,038 (1,233-2,791) | NA |
| Szeszenia-Dąbrowska, 2006(12) | 2005 | Poland | 3,249 | 35 |
| Vainauskas, 2010(13) | 1999-2008 | Lithuania | 928 (570-1,447) | 64 (41-97) |
| Szeszenia-Dąbrowska, 2013(14) | 1998-2011 | Poland | 5,035 (2,562-12,017) | 50 (25-117) |
| Oksa, 2019(15) | 1975, 2005, 2013 | Finland | NA | 253 (200-310)* |
| *Data type: workers’ compensation claims* | | | | |
| Kraut, 1994(16) | 1989 | Canada | 37,927 | 28.2 |
| Shih, 2023(11) | 2008-2021 | Taiwan | 752 (426-1,149) | NA |
| Kourouklis, 2009(17) | 2003-2007 | Greece | 34 | 1.71 |
| Medeni, 2024(10) | 2018-2022 | Turkey | 1,042 (955-1,209) | 3.64 (3.10-4.20) |
| *All type of occupational diseases in a specific sector* | | | | |
| *Agriculture sector* | | | | |
| Stocks, 2010(18) | 2002-2008 | UK | 33 (19-46) | NA |
| Karttunen, 2013(19) | 1982-2008 | Finland | 352 | NA |
| Szeszenia-Dąbrowska, 2016(20) | 2000-2014 | Poland | 229 (141-340) | 12.8 (5-14.6) |
| van der Molen, 2020(21) | 2004-2017 | Italy | 6431 | 1295 |
| Samant, 2020(22) | 2007-2016 | Norway | 47 (26-66) | 114 (68-169) |
| *Health sector* | | | | |
| Walsh, 2005(23) | 2002-2003 | UK | 5508 | NA |
| Zhou, 2017(24) | 2001-2014 | GB | 146 | 515 |
| *Construction sector* | | | | |
| Stocks, 2011(25) | 2002-2008 | UK | 264 | NA |

Note: IR= incidence rate; *Incidence rate in the study was measured in average incidence rate per 10,000 employees, this number has been adjusted to incidence per 100,000 employees. UK= United Kingdom, GB= Great Britain; NI = Northern Ireland; RoI = Republic of Ireland; NA= not available. Hussey, 2010(a)= cases reported by occupational physicians; Hussey, 2010(b)= cases reported by general practitioners. Hussey, 2013(a)= cases reported by general practitioners; Hussey, 2013(b)= cases reported by clinical specialists.

1. Cherry N, Meyer JD, Adisesh A, Brooke R, Owen-Smith V, Swales C, et al. Surveillance of occupational skin disease: EPIDERM and OPRA. Br J Dermatol. 2000;142(6):1128-34.

2. Chen Y, Turner S, McNamee R, Ramsay CN, Agius RM. The reported incidence of work-related ill-health in Scotland (2002-2003). Occup Med (Lond). 2005;55(4):252-61.

3. Chen Y, Turner S, Hussey L, Agius R. A study of work-related musculoskeletal case reports to The Health and Occupation Reporting network (THOR) from 2002 to 2003. Occup Med (Lond). 2005;55(4):268-74.

4. Hussey L, Turner S, Thorley K, McNamee R, Agius R. Work-related ill health in general practice, as reported to a UK-wide surveillance scheme. Br J Gen Pract. 2008;58(554):637-40.

5. Hussey L, Turner S, Thorley K, McNamee R, Agius R. Comparison of work-related ill health reporting by occupational physicians and general practitioners. Occup Med (Lond). 2010;60(4):294-300.

6. van der Molen HF, Kuijer PP, Smits PB, Schop A, Moeijes F, Spreeuwers D, et al. Annual incidence of occupational diseases in economic sectors in The Netherlands. Occup Environ Med. 2012;69(7):519-21.

7. Hussey L, Carder M, Money A, Turner S, Agius R. Comparison of work-related ill-health data from different GB sources. Occup Med (Lond). 2013;63(1):30-7.

8. Carder M, Money A, Turner S, Agius R. Workforce coverage by GB occupational physicians and disease incidence rates. Occup Med (Lond). 2014;64(4):271-8.

9. Money A, Carder M, Noone P, Bourke J, Hayes J, Turner S, et al. Work-related ill-health: Republic of Ireland, Northern Ireland, Great Britain 2005-2012. Occup Med (Lond). 2015;65(1):15-21.

10. Medeni İ, Alagüney ME, Medeni V. Medical and legal diagnoses comparison of the occupational diseases: A nationwide study in Turkey. J Eval Clin Pract. 2024;30(7):1449-56.

11. Shih P, Chu PC, Huang CC, Guo YL, Chen PC, Su TC. Hospital Occupational Health Service Network and Reporting Systems in Taiwan From 2008 to 2021. J Occup Environ Med. 2023;65(2):e43-e50.

12. Szeszenia-Dabrowska N, Wilczyńska U. Occupational diseases in the period of socioeconomic transition in Poland. Int J Occup Med Environ Health. 2006;19(2):99-106.

13. Vainauskas S, Venckienė R, Krisiulevičienė D, Chomentauskas A, Januškevičius V, Vasilavičius P. Trends in the incidence of occupational diseases in Lithuania between 1999 and 2008. Int J Occup Med Environ Health. 2010;23(4):317-22.

14. Szeszenia-Dąbrowska N, Wilczyńska U. Occupational diseases in Poland--an overview of current trends. Int J Occup Med Environ Health. 2013;26(3):457-70.

15. Oksa P, Sauni R, Talola N, Virtanen S, Nevalainen J, Saalo A, et al. Trends in occupational diseases in Finland, 1975-2013: a register study. BMJ Open. 2019;9(4):e024040.

16. Kraut A. Estimates of the extent of morbidity and mortality due to occupational diseases in Canada. Am J Ind Med. 1994;25(2):267-78.

17. Kourouklis GN. Disability from occupational diseases in Greece. Occup Med (Lond). 2009;59(7):515-7.

18. Stocks SJ, Turner S, Carder M, Hussey L, McNamee R, Agius RM. Medically reported work-related ill-health in the UK agricultural sector. Occup Med (Lond). 2010;60(5):340-7.

19. Karttunen JP, Rautiainen RH. Distribution and characteristics of occupational injuries and diseases among farmers: a retrospective analysis of workers' compensation claims. Am J Ind Med. 2013;56(8):856-69.

20. Szeszenia-Dąbrowska N, Świątkowska B, Wilczyńska U. Occupational diseases among farmers in Poland. Med Pr. 2016;67(2):163-71.

21. van der Molen HF, Marsili C, Vitali A, Colosio C. Trends in occupational diseases in the Italian agricultural sector, 2004-2017. Occup Environ Med. 2020;77(5):340-3.

22. Samant Y, Aas O, Ekle R, Gravseth HM, Strømholm T. Physician Notified Work-Related Diseases Among Farmers in Norway: Data from 2007 - 2016. J Agromedicine. 2020;25(2):201-9.

23. Walsh L, Turner S, Lines S, Hussey L, Chen Y, Agius R. The incidence of work-related illness in the UK health and social work sector: The Health and Occupation Reporting network 2002-2003. Occup Med (Lond). 2005;55(4):262-7.

24. Zhou AY, Carder M, Gittins M, Agius R. Work-related ill health in doctors working in Great Britain: incidence rates and trends. Br J Psychiatry. 2017;211(5):310-5.

25. Stocks SJ, Turner S, McNamee R, Carder M, Hussey L, Agius RM. Occupation and work-related ill-health in UK construction workers. Occup Med (Lond). 2011;61(6):407-15.
