# Supplementary material for: Global reporting and underreporting of occupational diseases: A systematic review
Source: PLoS One. 2026 Mar 26;21(3):e0345318. doi: 10.1371/journal.pone.0345318 (PMC13020801; doi:10.1371/journal.pone.0345318)
Supplement: S7 Table — (DOCX) [file pone.0345318.s007.docx]

**Table 4. Most studied occupational diseases by region**

| Region | Type of occupational disease | n of studies | n of years covered | Average n of annual cases (min-max) |
| --- | --- | --- | --- | --- |
| Europe | Skin diseases | 16 | 6.7 (1-14) | 1,268 (290 – 2,095) |
|  | Respiratory diseases | 12 | 7.7 (1-23) | 1,384 (598 – 3,217) |
|  | Cancer | 11 | 14.2 (3-36) | 160 (4 - 754) |
|  | Musculoskeletal disorders | 5 | 5.6 (2-12) | 1,653 (124 - 4,686) |
|  | Asthma | 6 | 8.8 (3-20) | 288 (53 – 545) |
|  | Infectious diseases | 5 | 3.5 (3-4) | 737 (72-1,402) |
|  | Mental illnesses | 4 | 6.2 (3-14) | 2,788 (526 - 4,709) |
|  | Hearing damage/loss | 2 | 6 (3-9) | 350 (244 – 540) |
|  | Asbestoses | 4 | 23 (20-30) | 58 (6 – 103) |
| Asia | Musculoskeletal disorders | 3 | 13.3 (1-25) | 4,537 (534-9,925) |
|  | Cancer | 3 | 16 (10-23) | 47 (28 - 75) |
|  | Infectious diseases | 1 | 20 | 145 |
|  | Asthma | 2 | 9.7 (6-15) | 30 (14-39) |
|  | Poisonings | 2 | 6.3 (1.6-11) | 645 (60-1,194) |
|  | Asbestoses | 2 | 13.5 (10-17) | 3,911 (86-7,736) |
|  | Skin diseases | 1 | 1.6 | 68 |
| Northern America | Skin diseases | 1 | 1 | 4,289 |
|  | Poisonings | 1 | 11 | 4,932 |
| Latin America | Skin diseases | 1 | 6 | 505 |
| Africa | Asthma | 1 | 2 | 113 |
|  | Respiratory diseases | 1 | 2 | 1,530 |
|  | Tuberculosis | 1 | 9 | 10 |
| Australia | Cancer | 2 | 14 (7-20) | 182 (81-284) |

Note: n of studies defined as number of studies reported that particular occupational disease in the region; n of years covered defined as the average number of years of data collection from all studies; average n of annual cases defined as the average number of cases reported per year, calculated by averaging the total number of cases divided by the number of years of each study. One study might investigate more than one occupational disease and appear more than once in this table. Studies that reported other outcomes, e.g., incident rate, change of incident rate, are not included in the table.
