# Supplementary material for: Global reporting and underreporting of occupational diseases: A systematic review
Source: PLoS One. 2026 Mar 26;21(3):e0345318. doi: 10.1371/journal.pone.0345318 (PMC13020801; doi:10.1371/journal.pone.0345318)
Supplement: S8 Table — (DOCX) [file pone.0345318.s008.docx]

**Table 5. Most reported occupational diseases by industrial sector**

| Industrial sector | Type of occupational disease | n of studies | n of years covered | Average n of annual cases (min-max) |
| --- | --- | --- | --- | --- |
| Health | Infectious disease | 3 | 8.7 (5-14) | 80 (8 – 187) |
|  | Tuberculosis | 4 | 9 (5-16) | 99 (12-291) |
|  | Mental illnesses | 1 | 4 | 239 (136-375)* |
|  | Skin disease | 1 | 13 | 42 |
| Agriculture | Skin disease | 1 | 8 | 13 |
|  | Cancer | 1 | 6 | 34 |
| Construction | Skin disease | 1 | 10 | 28 |
|  | Asthma | 1 | 1 | 1,031 |
|  | Respiratory disease | 2 | 4 (1-10) | 245 (4-414) |
|  | Cancer | 2 | 20 (13-26) | 571 (480-661) |
| Mining and quarrying | Musculoskeletal disorder | 1 | 12 | 123 |
|  | Respiratory disease | 1 | 10 | 6,183 |
|  | Poisoning | 1 | 10 | 295 |

Note: In this table, we included studies which focused on a specific industrial sector and reported the number of occupational disease cases. Some studies investigated more than one type of occupational disease. Average number of cases per year is estimated by the total number of cases per year divided by total number of studies. *The cases were reported by general practitioners, psychiatrists, and occupational physicians.
