# Supplementary material for: Global reporting and underreporting of occupational diseases: A systematic review
Source: PLoS One. 2026 Mar 26;21(3):e0345318. doi: 10.1371/journal.pone.0345318 (PMC13020801; doi:10.1371/journal.pone.0345318)
Supplement: S9 Table — (DOCX) [file pone.0345318.s009.docx]

**Table 6. Underreporting of occupational diseases**

| Author, year | Reported period (year) | Country | Type of disease | n cases reported | n of cases not reported | Underreporting rate (%) |
| --- | --- | --- | --- | --- | --- | --- |
| Skov, 1990(1) | 1983-1987 | Denmark | Cancer | 78 | 178 | 50 |
| Moreno-Torres, 2018(2) | 2000-2015 | Mexico | All | NA | NA | 89 (82-95) |

1. Skov T, Mikkelsen S, Svane O, Lynge E. Reporting of occupational cancer in Denmark. Scand J Work Environ Health. 1990;16(6):401-5.

2. Moreno-Torres LA, Ventura-Alfaro CE. Underreporting trends of occupational illnesses in Mexico. J Occup Health. 2018;60(1):85-8.
