# Supplementary material for: Global reporting and underreporting of occupational diseases: A systematic review
Source: PLoS One. 2026 Mar 26;21(3):e0345318. doi: 10.1371/journal.pone.0345318 (PMC13020801; doi:10.1371/journal.pone.0345318)
Supplement: S10 Table — (DOCX) [file pone.0345318.s010.docx]

**Table 7. Factors contributing to the underreporting of occupational diseases**

| Author, year | Country | Type of OD | Method | Type of respondents | Contributing factors |
| --- | --- | --- | --- | --- | --- |
| Arnaud, 2010(1) | France | All | Telephone study | Physicians | - Difficulties in diagnosing ODs - Lack of knowledge of the reporting system - Perceived difficulties in the process of making a claim - Concerns about employee job security |
| Parhar, 2011(2) | Canada | Asthma | Postal survey | Pulmonologists | - Lack of knowledge of the reporting system - Concerns about employee job security |
| Lysdal, 2011(3) | Denmark | Hand eczema | Postal survey | Hairdressers | - Unaware of workplace risks and disease causation - Perceived difficulties in the claiming process - Lack of knowledge of the reporting system |
| Moldovan, 2017(4) | 16 Eastern European countries | All | Online survey | Official national representatives | - Employer’s perceived as loss of revenue - Concerns about employee’s job security - Improper monitoring by the authorities |
| Fagan, 2017(5) | US | All | Inspections | Employers | - Lack of supervision and training of the onsite medical unit - No clear structure and policies governing the onsite medical unit |
| Alaguney, 2020(6) | Turkey | All | Online survey | Physicians | - Employer’s perceived as loss of revenue - Concerns about employee’s job security, in particular, those working as subcontractor and those without a legal contract |
| Cheng, 2022(7) | Taiwan | Asbestos-related diseases | In-depth interviews | Employees | - Unaware of workplace risks and disease causation - Perceived difficulties in the claiming process - Lack of knowledge of the reporting system - Concerns about employee’s job security |
| Karabağ, 2023(8) | Turkey |  | Modified delphi study | Physicians | - Compensation-oriented system - Fear stigmatization and loss of income of the employee - Lack of occupational disease surveillance - Insufficient knowledge and experience of physicians - Inability to diagnose occupational diseases and insufficient training during medical education - Lack of an institutional strategy of Ministry of Health regarding the employment of occupational medicine specialists - Lack of recognition of the occupational medicine specialists - Low number of occupational medicine specialists - Lack of knowledge of the reporting system |

1. Arnaud S, Cabut S, Viau A, Souville M, Verger P. Different reporting patterns for occupational diseases among physicians: a study of French general practitioners, pulmonologists and rheumatologists. Int Arch Occup Environ Health. 2010;83(3):251-8.

2. Parhar A, Lemiere C, Beach JR. Barriers to the recognition and reporting of occupational asthma by Canadian pulmonologists. Can Respir J. 2011;18(2):90-6.

3. Lysdal SH, Søsted H, Johansen JD. Do hairdressers in Denmark have their hand eczema reported as an occupational disease? Results from a register-based questionnaire study. Contact Dermatitis. 2012;66(2):72-8.

4. Moldovan HR, Voidazan ST, John SM, Weinert P, Moldovan G, Vlasiu MA, et al. The Eastern European experience on occupational skin diseases. Make underreporting an issue? J Eur Acad Dermatol Venereol. 2017;31 Suppl 4:5-11.

5. Fagan KM, Hodgson MJ. Under-recording of work-related injuries and illnesses: An OSHA priority. J Safety Res. 2017;60:79-83.

6. Alaguney ME, Yildiz AN, Demir AU, Ergor OA. Physicians' opinions about the causes of underreporting of occupational diseases. Arch Environ Occup Health. 2020;75(3):165-76.

7. Cheng Y, Huang YL, Lee LJ. Explaining the Invisibility of Asbestos-Related Diseases in the Taiwan Workers' Compensation System. New Solut. 2022;32(2):106-18.

8. Karabağ İ, Alagüney ME, Şahan C, Yıldız AN. How difficult is it to diagnose and report an occupational disease in a developing country? A modified delphi study. Acta Medica. 2023;54(4):347-56.
