# Supplementary material for: Global reporting and underreporting of occupational diseases: A systematic review
Source: PLoS One. 2026 Mar 26;21(3):e0345318. doi: 10.1371/journal.pone.0345318 (PMC13020801; doi:10.1371/journal.pone.0345318)
Supplement: S11 File — (DOCX) [file pone.0345318.s011.docx]

**List of excluded articles**

| No | Author | Published Year | Exclusion reason |
| --- | --- | --- | --- |
| 1 | Samant(1) | 2015 | Ineligible study type |
| 2 | Reynolds(2) | 2019 | Ineligible study type |
| 3 | Hernández Martín(3) | 2019 | Ineligible setting |
| 4 | Landrigan(4) | 1989 | Ineligible study type |
| 5 | De Bono(5) | 2021 | Ineligible setting |
| 6 | Loewenson(6) | 1999 | Ineligible study type |
| 7 | Chu(7) | 2013 | Full text unavailable |
| 8 | Leigh(8) | 2014 | Ineligible study outcome |
| 9 | Reilly(9) | 2020 | Ineligible setting |
| 10 | Giang(10) | 2006 | Full text unavailable |
| 11 | Wu(11) | 1996 | Ineligible study type |
| 12 | Aekplakorn(12) | 2002 | Ineligible study outcome |
| 13 | Sallie(13) | 1994 | Ineligible study type |
| 14 | Joe(14) | 2014 | Ineligible setting |
| 15 | Silverstein(15) | 1997 | Ineligible setting |
| 16 | Azaroff(16) | 2002 | Ineligible study type |
| 17 | Rosenman(17) | 2006 | Ineligible setting |
| 18 | Curti(18) | 2016 | Ineligible indication |
| 19 | Choi(19) | 1996 | Ineligible study type |
| 20 | Fouquet(20) | 2015 | Ineligible setting |
| 21 | Marinaccio(21) | 2011 | Ineligible study outcome |
| 22 | Walls(22) | 1997 | Full text unavailable |
| 23 | Tonozzi(23) | 2016 | Ineligible study population |
| 24 | Wuellner(24) | 2017 | Ineligible setting |
| 25 | Hall(25) | 2019 | Ineligible indication |
| 26 | Seaton(26) | 1991 | Ineligible study type |
| 27 | Bhandari(27) | 2016 | Ineligible study population |
| 28 | Samant(28) | 2008 | Full text unavailable |
| 29 | García Gómez(29) | 2013 | Ineligible setting |
| 30 | Marinaccio(30) | 2012 | Ineligible indication |
| 31 | Henneberger(31) | 1999 | Ineligible setting |
| 32 | Abas(32) | 2008 | Full text unavailable |
| 33 | Morse(33) | 2005 | Ineligible setting |
| 34 | Leigh(34) | 1998 | Full text unavailable |
| 35 | Melius(35) | 1989 | Ineligible study outcome |
| 36 | Rosenman(36) | 2003 | Ineligible setting |
| 37 | Corriols(37) | 2008 | Ineligible study population |
| 38 | CDC(38) | 1989 | Full text unavailable |
| 39 | Szeszenia-Dabrowska(39) | 2004 | Full text unavailable |
| 40 | Blum(40) | 1995 | Article not in English |
| 41 | Walls(41) | 2000 | Ineligible study type |
| 42 | Wu(42) | 1996 | Ineligible study type |
| 43 | Sabroe(43) | 1993 | Article not in English |
| 44 | Jones(44) | 1997 | Ineligible setting |
| 45 | To(45) | 2011 | Ineligible setting |
| 46 | CDC(46) | 2006 | Ineligible study type |
| 47 | Spreeuwers(47) | 2008 | Ineligible study outcome |
| 48 | Amar(48) | 2012 | Ineligible study population |
| 49 | Troke(49) | 2021 | Ineligible setting |
| 50 | Stanbury(50) | 2003 | Ineligible setting |
| 51 | Marinaccio(51) | 2020 | Full text unavailable |
| 52 | Sritharan(52) | 2019 | Ineligible setting |
| 53 | Hansen(53) | 2007 | Article not in English |
| 54 | Slabber(54) | 1994 | Full text unavailable |
| 55 | Spreeuwers(55) | 2010 | Ineligible study outcome |
| 56 | Samant(56) | 2014 | Article not in English |
| 57 | Simpson(57) | 2002 | Ineligible study type |
| 58 | Schulte(58) | 2005 | Ineligible study type |
| 59 | Scott(59) | 2004 | Ineligible study type |
| 60 | Heptonstall(60) | 1993 | Full text unavailable |
| 61 | Morse(61) | 1999 | Ineligible setting |
| 62 | Behrens(62) | 1994 | Ineligible study type |
| 63 | Hytönen(63) | 1997 | Ineligible study outcome |
| 64 | Goe(64) | 2004 | Ineligible setting |
| 65 | Reilly(65) | 2018 | Ineligible setting |
| 66 | Morse(66) | 2001 | Ineligible setting |
| 67 | Morse(67) | 2005 | Ineligible setting |
| 68 | Teschke(68) | 1992 | Ineligible setting |
| 69 | Jajosky(69) | 1999 | Ineligible setting |
| 70 | Boden(70) | 2008 | Ineligible setting |
| 71 | Malo(71) | 2000 | Ineligible indication |
| 72 | Fabiánová(72) | 1999 | Ineligible study type |
| 73 | Morse(73) | 2000 | Ineligible setting |
| 74 | Mirer(74) | 1989 | Ineligible study type |
| 75 | Marinaccio(75) | 2006 | Full text unavailable |
| 76 | Campo(76) | 2015 | Ineligible setting |
| 77 | Leigh(77) | 2001 | Ineligible setting |
| 78 | Idrovo(78) | 2003 | Full text unavailable |
| 79 | Chan-Yeung(79) | 2007 | Ineligible study type |
| 80 | Meredith(80) | 1995 | Full text unavailable |
| 81 | Lysdal | 2012 | Ineligible study type |
| 82 | Suruda(81) | 1988 | Ineligible study outcome |
| 83 | Kyung (82) | 2023 | Ineligible study outcome |
| 84 | Wilczyńska(83) | 2008 | Article not in English |
| 85 | Leigh(84) | 1997 | Full text unavailable |
| 86 | Hannaford-Turner(85) | 2010 | Ineligible setting |
| 87 | Mejia(86) | 2015 | Article not in English |
| 88 | Park(87) | 2021 | Ineligible setting |
| 89 | Kor(88) | 2001 | Full text unavailable |
| 90 | Urban(89) | 2000 | Full text unavailable |
| 91 | Lekei(90) | 2016 | Ineligible setting |
| 92 | Greife(91) | 2004 | Ineligible study outcome |
| 93 | Lee(92) | 1991 | Ineligible study type |
| 94 | Pransky(93) | 1999 | Ineligible study type |
| 95 | Saretto(94) | 2001 | Article not in English |
| 96 | Myers(95) | 2001 | Ineligible study type |
| 97 | García Gómez(96) | 2012 | Full text unavailable |
| 98 | Prado(97) | 2017 | Ineligible study type |
| 99 | Ross(98) | 1998 | Full text unavailable |
| 100 | McClure(99) | 2021 | Ineligible setting |
| 101 | Rosenman(100) | 1997 | Ineligible setting |
| 102 | Vandenplas(101) | 2005 | Full text unavailable |
| 103 | Bignon(102) | 1979 | Ineligible study population |
| 104 | Andersson(103) | 1995 | Ineligible setting |
| 105 | Liu(104) | 2023 | Article not in English |
| 106 | Koh (105) | 1998 | Ineligible study type |
| 107 | Carnide(106) | 2023 | Ineligible setting |
| 108 | Zellner(107) | 2022 | Article not in English |
| 109 | MacKinnon(108) | 2023 | Ineligible setting |
| 110 | Reilly(109) | 2019 | Ineligible setting |
| 111 | Coggon(110) | 2022 | Ineligible study type |
| 112 | Chong(111) | 2022 | Ineligible study type |
| 113 | Chen(112) | 2022 | Ineligible study type |
| 114 | Samant(113) | 2022 | Ineligible study type |
| 115 | Chang Leon(114) | 2022 | Ineligible study type |
| 116 | Zeng(115) | 2021 | Ineligible setting |
| 117 | De Bono(116) | 2021 | Ineligible setting |
| 118 | Ziembicki(117) | 2021 | Ineligible setting |
| 119 | Tustin(118) | 2021 | Ineligible study type |
| 120 | Brook(119) | 2021 | Ineligible setting |
| 121 | Kwon(120) | 2016 | Full text unavailable |
| 122 | Logar-Henderson(121) | 2019 | Ineligible setting |
| 123 | Chen(122) | 2019 | Ineligible study type |
| 124 | Benavides(123) | 2019 | Ineligible setting |
| 125 | Lasfargues(124) | 2019 | Ineligible study type |
| 126 | Cannavo(125) | 2018 | Full text unavailable |
| 127 | Chang(126) | 2018 | Ineligible study type |
| 128 | Won(127) | 2018 | Full text unavailable |
| 129 | Chen(122) | 2018 | Ineligible study type |
| 130 | Modenese(128) | 2018 | Ineligible study type |
| 131 | Guo(129) | 2018 | Ineligible study type |
| 132 | Curti(130) | 2018 | Ineligible study outcome |
| 133 | Mansyur(131) | 2018 | Ineligible study type |
| 134 | Hui Chen(132) | 2018 | Full text unavailable |
| 135 | Flynn(133) | 2018 | Full text unavailable |
| 136 | Arrandale(134) | 2018 | Ineligible setting |
| 137 | Drossard(135) | 2016 | Ineligible study type |
| 138 | Rappin(136) | 2016 | Ineligible setting |
| 139 | Wuellner(137) | 2016 | Ineligible setting |
| 140 | Barber(138) | 2015 | Ineligible study type |
| 141 | Mancha(139) | 2015 | Full text unavailable |
| 142 | Valenty(140) | 2015 | Ineligible study outcome |
| 143 | Bhattacharya(141) | 2014 | Ineligible study outcome |
| 144 | Thepaksorn(142) | 2014 | Ineligible study outcome |
| 145 | Davis(143) | 2014 | Ineligible setting |
| 146 | Barrick(144) | 2013 | Ineligible study type |
| 147 | Grattan(145) | 2013 | Ineligible setting |
| 148 | Myong(146) | 2013 | Ineligible study outcome |
| 149 | Pelclova(147) | 2011 | Full text unavailable |
| 150 | Riviere(148) | 2011 | Ineligible study type |
| 151 | Gisquet(149) | 2011 | Article not in English |
| 152 | Choi(150) | 2010 | Ineligible study outcome |
| 153 | Garcia Gomez(151) | 2009 | Article not in English |
| 154 | Karadzinska-Bislimovska(152) | 2010 | Full text unavailable |
| 155 | Bresciani(153) | 2007 | Full text unavailable |
| 156 | Arnaud(134) | 2008 | Full text unavailable |
| 157 | Verger(154) | 2008 | Full text unavailable |
| 158 | Mammone(155) | 2007 | Ineligible study type |
| 159 | Emmert(156) | 2006 | Full text unavailable |
| 160 | Chen(157) | 2005 | Article not in English |
| 161 | Axelopoulos(158) | 2005 | Ineligible study type |
| 162 | Morken(159) | 2005 | Article not in English |
| 163 | Lezaun(160) | 2003 | Full text unavailable |
| 164 | Diepgen(161) | 2002 | Article not in English |
| 165 | Pelclova(162) | 2001 | Full text unavailable |
| 166 | Pelclova(163) | 2000 | Full text unavailable |
| 167 | Wu(11) | 1996 | Ineligible study type |
| 168 | Gou(164) | 1999 | Ineligible study type |
| 169 | Koton(165) | 1998 | Full text unavailable |
| 170 | Davidson(166) | 1996 | Full text unavailable |
| 171 | Webster(167) | 1994 | Ineligible setting |
| 172 | Gaffuri(168) | 1991 | Full text unavailable |
| 173 | Nielsen(169) | 1979 | Full text unavailable |
| 174 | Kuhl(170) | 1974 | Article not in English |
| 175 | Girard(171) | 1972 | Article not in English |
| 176 | Ilgenfritz(172) | 1966 | Full text unavailable |
| 177 | Lele(173) | 2018 | Ineligible study type |
| 178 | Jajosky(69) | 1999 | Ineligible setting |
| 179 | Rautiainen(174) | 2005 | Full text unavailable |
| 180 | Islam(175) | 2000 | Ineligible setting |
| 181 | Friedman-Jimenez(176) | 1994 | Full text unavailable |
| 182 | Leman(177) | 2010 | Full text unavailable |
| 183 | Siang(178) | 2010 | Ineligible study type |
| 184 | Goldberg(179) | 2006 | Ineligible study outcome |
| 185 | Koehoorn(180) | 2006 | Ineligible setting |
| 186 | Salim(181) | 2014 | Ineligible study type |
| 187 | Kim(182) | 2010 | Full text unavailable |
| 188 | Malo(183) | 2000 | Ineligible study type |
| 189 | Kawakami(184) | 2010 | Full text unavailable |
| 190 | Hnizdo(185) | 1999 | Full text unavailable |
| 191 | Peyrethon | 2022 | Full text unavailable |
| 192 | Barlet(186) | 2022 | Full text unavailable |
| 193 | Chen(187) | 2018 | Article not in English |
| 194 | Ruser(188) | 2008 | Ineligible study type |
| 195 | Staniszewska(189) | 2006 | Article not in English |
| 196 | Morse(190) | 2000 | Ineligible setting |
| 197 | Freund(191) | 1990 | Full text unavailable |
| 198 | CDC(192) | 1990 | Ineligible setting |
| 199 | Muldoon(193) | 1987 | Ineligible setting |
| 200 | Melius(35) | 1989 | Ineligible setting |
| 201 | Zhou(194) | 2020 | Ineligible study type |
| 202 | Ahn(195) | 2010 | Ineligible study type |
| 203 | Mazurek(196) | 2008 | Ineligible setting |
| 204 | Teschke(197) | 1992 | Full text unavailable |
| 205 | Peyrethon(198) | 2022 | Full text unavailable |
| 206 | Brunier(199) | 2022 | Full text unavailable |
| 207 | Rachiotis(200) | 2012 | Full text unavailable |
| 208 | Dulon | 2020 | Full text unavailable |
| 209 | Ndiaye(201) | 2014 | Full text unavailable |
| 210 | Dickel(202) | 2002 | Ineligible setting |
| 211 | Hagemeyer(203) | 2005 | Full text unavailable |
| 212 | Nilson(204) | 1992 | Ineligible study outcome |
| 213 | Stolovas(205) | 2021 | Article not in English |
| 214 | Stephenson(206) | 2005 | Full text unavailable |
| 215 | Cavalli(207) | 2020 | Ineligible study type |
| 216 | Fan(208) | 2016 | Ineligible study type |
| 217 | La See(209) | 2019 | Ineligible setting |
| 218 | Spreeuwers(210) | 2009 | Ineligible study outcome |
| 219 | Lenderink(211) | 2009 | Ineligible indication |
| 220 | Menzel(212) | 2008 | Ineligible setting |
| 221 | Chaiklieng(213) | 2023 | Ineligible setting |
| 222 | Wang(214) | 2022 | Ineligible study outcome |
| 223 | Baur(215) | 1998 | Ineligible study type |
| 224 | Kathare(216) | 2022 | Ineligible study outcome |
| 225 | Yildiz(217) | 2018 | Ineligible study outcome |
| 226 | Davoodi(218) | 2017 | Ineligible study type |
| 227 | Keegel(219) | 2009 | Ineligible study outcome |
| 228 | Anderson(220) | 2011 | Ineligible setting |
| 229 | Murphy(221) | 1996 | Ineligible study outcome |
| 230 | Piedrahita(222) | 2006 | Ineligible indication |
| 231 | Hämäläinen(223) | 2011 | Full text unavailable |
| 232 | Kirkham(224) | 2010 | Ineligible setting |
| 233 | Oyunbileg(225) | 2011 | Full text unavailable |
| 234 | Filon(226) | 2021 | Ineligible study outcome |
| 235 | Cohen(227) | 2023 | Ineligible study outcome |
| 236 | Tanaka(228) | 1995 | Ineligible study type |
| 237 | Anderson(229) | 2010 | Ineligible setting |
| 238 | Waclawski(230) | 2013 | Ineligible study type |
| 239 | Muula(231) | 2010 | Ineligible study type |
| 240 | Shi(232) | 2021 | Ineligible study outcome |
| 241 | Zhang(233) | 2010 | Ineligible indication |
| 242 | Gerr(234) | 2008 | Full text unavailable |
| 243 | Zhou(235) | 2022 | Ineligible setting |
| 244 | Largo(236) | 2013 | Ineligible setting |
| 245 | Liss(237) | 2002 | Ineligible study type |
| 246 | Thuerauf(238) | 1996 | Ineligible study outcome |
| 247 | DelBianco(239) | 2013 | Ineligible study outcome |
| 248 | Masterson(240) | 2015 | Ineligible study outcome |
| 249 | Volinn(241) | 2005 | Ineligible setting |
| 250 | Hung(242) | 2016 | Ineligible indication |
| 251 | Nienhaus(243) | 2020 | Ineligible study outcome |
| 252 | Marinaccio(244) | 2018 | Ineligible study population |
| 253 | Almberg(245) | 2018 | Ineligible study population |
| 254 | García-Gómez(246) | 2014 | Full text unavailable |
| 255 | Binazzi(247) | 2017 | Ineligible study population |
| 256 | Lehnert(248) | 2016 | Ineligible study population |
| 257 | Pickard(249) | 2022 | Ineligible setting |
| 258 | Szeszenia-Dabrowska(250) | 2013 | Full text unavailable |
| 259 | Smith(251) | 2016 | Ineligible study outcome |
| 260 | Lee(252) | 2018 | Ineligible study outcome |
| 261 | da Costa(253) | 2015 | Ineligible study type |
| 262 | Marinaccio(254) | 2011 | Ineligible study outcome |
| 263 | Fishwick(255) | 2022 | Ineligible study outcome |
| 264 | Lytras(256) | 2016 | Ineligible study population |
| 265 | Frazier(257) | 1994 | Ineligible setting |
| 266 | Takala(258) | 2014 | Ineligible study type |
| 267 | Salo(259) | 2017 | Ineligible study population |
| 268 | Shi(260) | 2020 | Ineligible study type |
| 269 | Kurth(261) | 2023 | Ineligible study population |
| 270 | Rosenman(262) | 2012 | Ineligible study type |
| 271 | Tarlo (263) | 2015 | Ineligible study type |
| 272 | Kauppinen(264) | 2012 | Ineligible study outcome |
| 273 | Karjalainen(265) | 2003 | Ineligible study outcome |
| 274 | Kang(266) | 2018 | Ineligible study type |
| 275 | Malo(267) | 2007 | Ineligible study type |
| 276 | von Hirschberg(268) | 2009 | Ineligible study outcome |
| 277 | Rosenman(269) | 1997 | Ineligible study type |
| 278 | Draper(270) | 2003 | Ineligible study type |
| 279 | Siddharthan(271) | 2006 | Ineligible study outcome |
| 280 | Loeppke(272) | 2017 | Ineligible study type |
| 281 | Spence(273) | 2001 | Ineligible study type |
| 282 | Govaerts(274) | 2021 | Ineligible study type |
| 283 | Vargas-Prada(275) | 2014 | Full text unavailable |
| 284 | Mengistu(276) | 2021 | Ineligible study outcome |
| 285 | Nanyan(277) | 2019 | Ineligible study outcome |
| 286 | van der Molen(278) | 2020 | Ineligible study type |
| 287 | Azaroff(279) | 2002 | Ineligible study type |
| 288 | O'Connell(280) | 2020 | Ineligible study type |
| 289 | Siddharthan(281) | 2006 | Ineligible study outcome |
| 290 | Min(282) | 2024 | Ineligible study outcome |
| 291 | Xu (283) | 2023 | Ineligible study type |
| 292 | Myers(284) | 2023 | Ineligible study type |
| 293 | Mongeau(285) | 2023 | Ineligible setting |
| 294 | Liu(104) | 2023 | Article not in English |
| 295 | Ping Hui Chen(286) | 2024 | Ineligible study type |
| 296 | Brans(287) | 2023 | Ineligible study type |
| 297 | Strømholm(288) | 2024 | Ineligible study type |
| 298 | Cheng | 2022 | Ineligible study type |

1. Samant Y, Wannag A, Urban P, Mattioli S. Sentinel surveillance and occupational disease. Occup Med (Lond). 2015;65(8):611-4.

2. Reynolds CJ, Blanc P. Into ploughshares: forging effective surveillance for work-related lung disease. Occup Environ Med. 2019;76(11):783-4.

3. Hernández Martín MM, Romero-Saldaña M, Pacheco Del Cerro JL, Alonso-Safont T, Molina-Recio G, Meneses Monroy A. Occupational and work-related disease underestimated and linked to temporary disability through Primary Health Care Services. J Nurs Manag. 2019;27(6):1140-7.

4. Landrigan PJ. Improving the surveillance of occupational disease. Am J Public Health. 1989;79(12):1601-2.

5. DeBono NL, Warden H, Logar-Henderson C, Shakik S, Dakouo M, MacLeod J, et al. Incidence of mesothelioma and asbestosis by occupation in a diverse workforce. Am J Ind Med. 2021;64(6):476-87.

6. Loewenson R. Assessment of the Health Impact of Occupational Risk in Africa: Current Situation and Methodological Issues. Epidemiology. 1999;10(5):632-9.

7. Chu PC, Fuh HR, Luo JC, Du CL, Chuang HY, Guo HR, et al. The impact of occupational health service network and reporting system in Taiwan. Int J Occup Environ Health. 2013;19(4):352-62.

8. Leigh JP, Du J, McCurdy SA. An estimate of the U.S. government's undercount of nonfatal occupational injuries and illnesses in agriculture. Ann Epidemiol. 2014;24(4):254-9.

9. Reilly MJ, Wang L, Rosenman KD. The Burden of Work-related Asthma in Michigan, 1988-2018. Ann Am Thorac Soc. 2020;17(3):284-92.

10. Giang GM. Epidemiology of work-related upper extremity disorders: understanding prevalence and outcomes to impact provider performances using a practice management reporting tool. Clin Occup Environ Med. 2006;5(2):267-83, vi.

11. Wu TN, Liou SH, Wang JD, Shen CY, Ko KN, Yang GY, et al. Establishment of a work-related diseases surveillance system in Taiwan, Republic of China. Prev Med. 1996;25(6):725-9.

12. Aekplakorn W, Suriyawongpaisal P, Methawikul T. The diagnosis and reporting of occupational diseases: the performance of physicians in Thailand. Southeast Asian J Trop Med Public Health. 2002;33(1):188-92.

13. Sallie BA, Ross DJ, Meredith SK, McDonald JC. SWORD '93. Surveillance of work-related and occupational respiratory disease in the UK. Occup Med (Lond). 1994;44(4):177-82.

14. Joe L, Roisman R, Beckman S, Jones M, Beckman J, Frederick M, et al. Using multiple data sets for public health tracking of work-related injuries and illnesses in California. Am J Ind Med. 2014;57(10):1110-9.

15. Silverstein BA, Stetson DS, Keyserling WM, Fine LJ. Work-related musculoskeletal disorders: comparison of data sources for surveillance. Am J Ind Med. 1997;31(5):600-8.

16. Azaroff LS, Levenstein C, Wegman DH. Occupational injury and illness surveillance: conceptual filters explain underreporting. Am J Public Health. 2002;92(9):1421-9.

17. Rosenman KD, Kalush A, Reilly MJ, Gardiner JC, Reeves M, Luo Z. How much work-related injury and illness is missed by the current national surveillance system? J Occup Environ Med. 2006;48(4):357-65.

18. Curti S, Sauni R, Spreeuwers D, De Schryver A, Valenty M, Rivière S, et al. Interventions to increase the reporting of occupational diseases by physicians: a Cochrane systematic review. Occup Environ Med. 2016;73(5):353-4.

19. Choi BC. Recording, notification, compilation, and classification of statistics of occupational accidents and diseases: the Thai experience. J Occup Environ Med. 1996;38(11):1151-60.

20. Fouquet N, Bodin J, Descatha A, Petit A, Ramond A, Ha C, et al. Prevalence of thoracic spine pain in a surveillance network. Occup Med (Lond). 2015;65(2):122-5.

21. Marinaccio A, Binazzi A, Marzio DD, Scarselli A, Verardo M, Mirabelli D, et al. Pleural malignant mesothelioma epidemic: Incidence, modalities of asbestos exposure and occupations involved from the Italian National Register. International Journal of Cancer. 2012;130(9):2146-54.

22. Walls C, Crane J, Gillies J, Wilsher M, Wong C. Occupational asthma and other nonasbestos occupational respiratory diseases notified between 1993 and 1996. N Z Med J. 1997;110(1047):246-9.

23. Tonozzi TR, Marsh SM, Reichard AA, Bhandari R. Reported work-related injuries and illnesses among Hispanic workers: Results from an emergency department surveillance system follow-back survey. Am J Ind Med. 2016;59(8):621-9.

24. Wuellner SE, Adams DA, Bonauto DK. Workers' compensation claims not reported in the Survey of Occupational Injuries and Illnesses: Injury and claim characteristics. Am J Ind Med. 2017;60(3):264-75.

25. Hall NB, Blackley DJ, Halldin CN, Laney AS. Continued increase in prevalence of r-type opacities among underground coal miners in the USA. Occup Environ Med. 2019;76(7):479-81.

26. Seaton A. Surveillance of work related and occupational respiratory disease--SWORD. Thorax. 1991;46(8):548.

27. Bhandari R, Marsh SM, Reichard AA, Tonozzi TR. Characterizing emergency department patients who reported work-related injuries and illnesses. Am J Ind Med. 2016;59(8):610-20.

28. Samant Y, Parker D, Wergeland E, Wannag A. The Norwegian Labour Inspectorate's Registry for Work-Related Diseases: data from 2006. Int J Occup Environ Health. 2008;14(4):272-9.

29. García Gómez M, Urbaneja Arrúe F, Markowitz S, Castañeda López R, Menduiña PL. Occupational diseases compensated in the Basque Country (Spain) from 1990 to 2008. Am J Ind Med. 2013;56(3):326-34.

30. Marinaccio A, Scarselli A, Merler E, Iavicoli S. Mesothelioma incidence surveillance systems and claims for workers’ compensation. Epidemiological evidence and prospects for an integrated framework. BMC Public Health. 2012;12(1):314.

31. Henneberger PK, Kreiss K, Rosenman KD, Reilly MJ, Chang YF, Geidenberger CA. An evaluation of the incidence of work-related asthma in the United States. Int J Occup Environ Health. 1999;5(1):1-8.

32. Abas AB, Said AR, Mohammed MA, Sathiakumar N. Occupational disease among non-governmental employees in Malaysia: 2002-2006. Int J Occup Environ Health. 2008;14(4):263-71.

33. Morse T, Dillon C, Kenta-Bibi E, Weber J, Diva U, Warren N, et al. Trends in work-related musculoskeletal disorder reports by year, type, and industrial sector: a capture-recapture analysis. Am J Ind Med. 2005;48(1):40-9.

34. Leigh JP, Miller TR. Occupational illnesses within two national data sets. Int J Occup Environ Health. 1998;4(2):99-113.

35. Melius JM, Sestito JP, Seligman PJ. Occupational disease surveillance with existing data sources. Am J Public Health. 1989;79 Suppl(Suppl):46-52.

36. Rosenman KD, Reilly MJ, Henneberger PK. Estimating the total number of newly-recognized silicosis cases in the United States. Am J Ind Med. 2003;44(2):141-7.

37. Corriols M, Marín J, Berroteran J, Lozano LM, Lundberg I, Thörn A. The Nicaraguan Pesticide Poisoning Register: constant underreporting. Int J Health Serv. 2008;38(4):773-87.

38. Occupational disease surveillance: carpal tunnel syndrome. MMWR Morb Mortal Wkly Rep. 1989;38(28):485-9.

39. Szeszenia-Dabrowska N, Wilczyńska U, Szymczak W, Pepłońska B. [Occupational diseases in Poland, 2003]. Med Pr. 2004;55(4):299-306.

40. Blum P. [Reporting occupational disease. A system ready for revision]. Tidsskr Nor Laegeforen. 1995;115(4):490-4.

41. Walls C, Crane J, Gillies J, Wilsher M, Wong C. Occupational asthma cases notified to OSH from 1996 to 1999. N Z Med J. 2000;113(1122):491-2.

42. Wu T-N, Liou S-H, Shen C-Y, Hsu C-C, Chao S-L, Chang P-Y. Occupational disease surveillance in Taiwan. The Lancet. 1996;348(9030):827.

43. S S. Were all occupationally conditioned complaints reported to the authority of occupational protection? Ugeskr Laeger. 1993;155(6):365.

44. SA JBR. Reporting occupational illnesses and injuries in North Carolina. An update for physicians. N C Med J. 1997;58(5):350-3.

45. To T, Tarlo SM, McLimont S, Haines T, Holness DL, Lougheed MD, et al. Feasibility of a provincial voluntary reporting system for work-related asthma in Ontario. Can Respir J. 2011;18(5):275-7.

46. Nonfatal occupational injuries and illnesses among workers treated in hospital emergency departments--United States, 2003. MMWR Morb Mortal Wkly Rep. 2006;55(16):449-52.

47. Spreeuwers D, de Boer AG, Verbeek JH, van Beurden MM, van Dijk FJ. Diagnosing and reporting of occupational diseases: a quality improvement study. Occup Med (Lond). 2008;58(2):115-21.

48. Amar RK, Jick SS, Rosenberg D, Maher TM, Meier CR. Incidence of the pneumoconioses in the United Kingdom general population between 1997 and 2008. Respiration. 2012;84(3):200-6.

49. Troke N, Logar-Henderson C, DeBono N, Dakouo M, Hussain S, MacLeod JS, et al. Incidence of acute myocardial infarction in the workforce: Findings from the Occupational Disease Surveillance System. Am J Ind Med. 2021;64(5):338-57.

50. Stanbury M, Reilly MJ, Rosenman KD. Work-related amputations in Michigan, 1997. Am J Ind Med. 2003;44(4):359-67.

51. Marinaccio A, Corfiati M, Binazzi A, Di Marzio D, Bonafede M, Verardo M, et al. The epidemiological surveillance of malignant mesothelioma in Italy (1993-2015): methods, findings, and research perspectives. Epidemiol Prev. 2020;44(1):23-30.

52. Sritharan J, MacLeod JS, Dakouo M, Qadri M, McLeod CB, Peter A, et al. Breast cancer risk by occupation and industry in women and men: Results from the Occupational Disease Surveillance System (ODSS). Am J Ind Med. 2019;62(3):205-11.

53. Hansen J, Rasmussen TR, Omland Ø, Olsen JH. [Registration of selected cases of occupational cancer (1994-2002) with the Danish National Board of Industrial Injuries]. Ugeskr Laeger. 2007;169(18):1674-8.

54. JC SCD. Reporting occupational disease. S Afr Med J. 1994;84(3):127-8.

55. Spreeuwers D, de Boer AG, Verbeek JH, van Dijk FJ. Evaluation of occupational disease surveillance in six EU countries. Occup Med (Lond). 2010;60(7):509-16.

56. Samant Y, Lysberg K, Landrø M, Eriksen T, Wergeland E. Doctors' reports of work-related hearing loss. Tidsskr Nor Laegeforen. 2014;134(20):1950-5.

57. Simpson WM, Jr. The incidence of green tobacco sickness among Latino farmworkers. J Occup Environ Med. 2002;44(3):225-6.

58. Schulte PA. Characterizing the burden of occupational injury and disease. J Occup Environ Med. 2005;47(6):607-22.

59. Scott DF, Grayson RL, Metz EA. Disease and illness in U.S. mining, 1983-2001. J Occup Environ Med. 2004;46(12):1272-7.

60. Heptonstall J; Gill ON PK, Black MB, Gilbart VL. Health care workers and HIV: surveillance of occupationally acquired infection in the United Kingdom. Communicable disease report CDR review. 1993;3(11):R147-53.

61. Morse T, Storey E. Fatalities from occupational diseases in Connecticut. Conn Med. 1999;63(8):463-6.

62. Behrens V, Seligman P, Cameron L, Mathias CG, Fine L. The prevalence of back pain, hand discomfort, and dermatitis in the US working population. Am J Public Health. 1994;84(11):1780-5.

63. Hytönen M, Kanerva L, Malmberg H, Martikainen R, Mutanen P, Toikkanen J. The risk of occupational rhinitis. Int Arch Occup Environ Health. 1997;69(6):487-90.

64. Goe SK, Henneberger PK, Reilly MJ, Rosenman KD, Schill DP, Valiante D, et al. A descriptive study of work aggravated asthma. Occup Environ Med. 2004;61(6):512-7.

65. Reilly MJ, Timmer SJ, Rosenman KD. The Burden of Silicosis in Michigan: 1988-2016. Ann Am Thorac Soc. 2018;15(12):1404-10.

66. Morse T, Dillon C, Warren N, Hall C, Hovey D. Capture-recapture estimation of unreported work-related musculoskeletal disorders in Connecticut. Am J Ind Med. 2001;39(6):636-42.

67. Morse T, Meyer JD, St Louis T, Storey E. Occupational disease in Connecticut: 2002. Conn Med. 2005;69(6):329-34.

68. Teschke K, Barroetavena MC. Occupational cancer in Canada: what do we know? Cmaj. 1992;147(10):1501-7.

69. Jajosky RA, Harrison R, Reinisch F, Flattery J, Chan J, Tumpowsky C, et al. Surveillance of work-related asthma in selected U.S. states using surveillance guidelines for state health departments--California, Massachusetts, Michigan, and New Jersey, 1993-1995. MMWR CDC Surveill Summ. 1999;48(3):1-20.

70. Boden LI, Ozonoff A. Capture-recapture estimates of nonfatal workplace injuries and illnesses. Ann Epidemiol. 2008;18(6):500-6.

71. Malo JL. How much adult asthma can be attributed to occupational factors (revisited)? Chest. 2000;118(5):1232-4.

72. Fabiánová E, Szeszenia-Dabrowska N, Kjaerheim K, Boffetta P. Occupational cancer in central European countries. Environ Health Perspect. 1999;107 Suppl 2(Suppl 2):279-82.

73. Morse T, Grey M, Storey E, Kenta-Bibi E. Occupational disease in Connecticut, 2000. Conn Med. 2002;66(12):723-30.

74. MA MFPRS. Epidemiologic and toxicologic evidence of occupational cancer in metalworking and transportation equipment industries: Undercounting occupational disease. Ann NY Acad Sci 1989;572:10-6.

75. Marinaccio A, Branchi C, Massari S, Scarselli A. National epidemiologic surveillance systems of asbestos-related disease and the exposed workers register. Med Lav. 2006;97(3):482-7.

76. Campo G, Papale A, Baldasseroni A, Di Leone G, Magna B, Martini B, et al. The surveillance of occupational diseases in Italy: the MALPROF system. Occup Med (Lond). 2015;65(8):632-7.

77. Leigh JP, Cone JE, Harrison R. Costs of occupational injuries and illnesses in California. Prev Med. 2001;32(5):393-406.

78. Idrovo AJ. [Estimate of the incidence of occupational diseases in Colombia, 1985-2000]. Rev Salud Publica (Bogota). 2003;5(3):263-71.

79. Chan-Yeung M, Becklake M. Occupational lung disease: Under-recognised, underestimated and poorly managed, even today. The international journal of tuberculosis and lung disease : the official journal of the International Union against Tuberculosis and Lung Disease. 2007;11:119.

80. Meredith S, McDonald C. Surveillance systems for occupational disease. The Annals of Occupational Hygiene. 1995;39(2):257-60.

81. Suruda A, Emmett EA. Counting recognized occupational deaths in the United States. J Occup Med. 1988;30(11):868-72.

82. Kyung M, Lee S-J, Dancu C, Hong O. Underreporting of workers’ injuries or illnesses and contributing factors: a systematic review. BMC Public Health. 2023;23(1):558.

83. Wilczyńska U, Szeszenia-Dabrowska N. [Occupational diseases caused by ionizing radiation in Poland, 1971-2006]. Med Pr. 2008;59(1):1-8.

84. Leigh JP, Markowitz SB, Fahs M, Shin C, Landrigan PJ. Occupational injury and illness in the United States. Estimates of costs, morbidity, and mortality. Arch Intern Med. 1997;157(14):1557-68.

85. Hannaford-Turner K, Elder D, Sim MR, Abramson MJ, Johnson AR, Yates DH. Surveillance of Australian workplace Based Respiratory Events (SABRE) in New South Wales. Occup Med (Lond). 2010;60(5):376-82.

86. Mejia CR, Cárdenas MM, Gomero-Cuadra R. [Notification of accidents and occupational diseases to the ministry of labor. Peru 2010-2014]. Rev Peru Med Exp Salud Publica. 2015;32(3):526-31.

87. Park JT, Yoon J. Why Workers Hesitate to Report Their Work-Related Musculoskeletal Symptoms: A Survey at a Korean Semiconductor Company. Int J Environ Res Public Health. 2021;18(21).

88. Kor AC, Lee HS, Chee CB, Wang YT. Occupational asthma in Singapore. Singapore Med J. 2001;42(8):373-7.

89. Urban P, Cikrt M, Hejlek A, Lukás E, Pelclová D. The Czech National Registry of Occupational Diseases. Ten years of existence. Cent Eur J Public Health. 2000;8(4):210-2.

90. Lekei EE, Ngowi AV, London L. Undereporting of acute pesticide poisoning in Tanzania: modelling results from two cross-sectional studies. Environ Health. 2016;15(1):118.

91. Greife A. Occupational health and safety surveillance. Work-RISQS--a national occupational injury and illness surveillance system. J Occup Environ Hyg. 2004;1(11):D119-20.

92. Lee HS, Phoon WH, Wang YT, Poh SC, Cheong TH, Yap JC, et al. Occupational asthma in Singapore--a review of cases from 1983 to 1990. Singapore Med J. 1991;32(6):398-402.

93. Pransky G, Snyder T, Dembe A, Himmelstein J. Under-reporting of work-related disorders in the workplace: a case study and review of the literature. Ergonomics. 1999;42(1):171-82.

94. Saretto G, Gianoli E, Tedesco R, Ferrari G, Catenacci G. [Analysis of occupational allergies reported to the Occupational Medicine and Hygiene Service of the Pavia ASL and to the Hospital Operative Unit of Occupational Medicine in 1993-98]. G Ital Med Lav Ergon. 2001;23(1):43-51.

95. Myers JP, Schettler T. Gaps in pesticide reporting lead to underestimates of risk. Environ Health Perspect. 2001;109(2):A64.

96. García Gómez M, Menéndez-Navarro A, Castañeda López R. [Incidence of asbestosis and other benign lung diseases: Spain, 1962-2010]. Rev Esp Salud Publica. 2012;86(6):613-25.

97. Prado JB, Mulay PR, Kasner EJ, Bojes HK, Calvert GM. Acute Pesticide-Related Illness Among Farmworkers: Barriers to Reporting to Public Health Authorities. J Agromedicine. 2017;22(4):395-405.

98. Ross DJ, Cherry NM, McDonald JC. Occupationally acquired infectious disease in the United Kingdom: 1996 to 1997. Commun Dis Public Health. 1998;1(2):98-102.

99. McClure LA, Koru-Sengul T, Hernandez MN, Caban-Martinez AJ, Kobetz EN, Lee DJ. Comparing cancer risk estimates using occupational record linkage approaches in male Florida firefighters. Am J Ind Med. 2021;64(2):78-83.

100. Rosenman KD, Reilly MJ, Kalinowski DJ. A state-based surveillance system for work-related asthma. J Occup Environ Med. 1997;39(5):415-25.

101. Vandenplas O, Larbanois A, Bugli C, Kempeneers E, Nemery B. [The epidemiology of occupational asthma in Belgium]. Rev Mal Respir. 2005;22(3):421-30.

102. Bignon J, Sebastien P, Di Menza L, Payan H. French mesothelioma register. Ann N Y Acad Sci. 1979;330:455-66.

103. Andersson E, Torén K. Pleural mesotheliomas are underreported as occupational cancer in Sweden. Am J Ind Med. 1995;27(4):577-80.

104. Liu AQ, Wang D, Li XX, Wu JH, Hu WJ. [Investigation and analysis of late reporting and under-reporting of occupational diseases from 2018 to 2020 in China]. Zhonghua Lao Dong Wei Sheng Zhi Ye Bing Za Zhi. 2023;41(5):353-7.

105. Koh D, Jeyaratnam J. Occupational health in Singapore. Int Arch Occup Environ Health. 1998;71(5):295-301.

106. Carnide N, Sritharan J, Song C, Macleod JS, Kooshki F, Furlan AD, et al. O-144 Incidence of opioid-related harms by occupation in Ontario, Canada: findings from the occupational disease surveillance system. Occupational and Environmental Medicine. 2023;80(Suppl 1):A10.

107. Zellner M, Jungmann O, Schöps W. Berufsbedingte Tumoren der ableitenden Harnwege – Häufigkeit, Meldeverhalten und administratives VorgehenOccupational cancer of the urinary tract—incidence, reporting behavior, and administrative procedures. Die Urologie. 2022;61.

108. MacKinnon M, Barrick K, Lévesque LE, Liss G, Tarlo SM, Lougheed MD. Linkage of administrative and compensation databases for work-related asthma surveillance in Ontario: A proof of concept study. Canadian Journal of Respiratory, Critical Care, and Sleep Medicine. 2023;7(1):28-35.

109. Reilly M, Wang L, Rosenman K. The Burden of Work-Related Asthma in Michigan, 1988-2018. Annals of the American Thoracic Society. 2019;17.

110. Coggon D. Estimating population burdens of occupational disease. Scand J Work Environ Health. 2022;48(2):83-5.

111. Chong HT, Collie A. The Characteristics of Accepted Work-related Injuries and Diseases Claims in the Australian Coal Mining Industry. Safety and Health at Work. 2022;13(2):135-40.

112. P.C. CPHC. Medical Accessibility and Under-reporting of Occupational Diseases: Effect of Travel Distance and Travel Time. Safety and Health at Work. 2022;13:S237.

113. Samant Y, Støver M, Stette-Haarberg I, Lohmann-Lafrenz S, Strømholm T. Physician Reported Work-Related COVID-19 cases in Norway 2020 – 2021.

114. D.G. CLAHGAGSKEC. Work-related musculoskeletal disorders - Official data study in the Republic of Ecuador. Safety and Health at Work. 2022;13(S158-159).

115. Zeng X, DeBono NL, Harris AM, Arrandale VH, Demers PA. Neurodegenerative diseases among miners in Ontario, Canada, using a linked cohort. Occup Environ Med. 2020.

116. DeBono NL, Logar-Henderson C, Warden H, Shakik S, Dakouo M, MacLeod J, et al. Cancer surveillance among workers in plastics and rubber manufacturing in Ontario, Canada. Occup Environ Med. 2020;77(12):847-56.

117. Ziembicki S, Arrandale VH, DeBono N, Dakouo M, Kirkham T, Demers P. O-24 Lung and bladder cancer surveillance among construction workers in diesel engine exhaust exposed occupations in Ontario, Canada. Occupational and Environmental Medicine. 2021;78(Suppl 1):A14.

118. M TASDWVH. Heat-related acute kidney injury in indoor and outdoor workers in the U.S. Occupational and Environmental Medicine. 2021;78:A20-A1.

119. Brook RA, Kleinman NL, Beren IA. Disability and workers' compensation trends for employees with mental disorders and SUDs in the United States. Ment Health Clin. 2021;11(5):279-86.

120. Y.-K. KS-CSJK. Estimation of Cases of Work-Related Asthma Using Capture-Recapture Methods. World Allergy Organization Journal. 2016;9:203.

121. Logar-Henderson C, MacLeod JS, Arrandale VH, Holness DL, McLeod CB, Peter A, et al. Adult Asthma among Workers in Ontario. Results from the Occupational Disease Surveillance System. Ann Am Thorac Soc. 2019;16(5):563-71.

122. Chen C-Y, Cheng Y. O3A.1 Assessing the under-estimation of occupational respiratory diseases in taiwan: analyses of disease burdens and healthcare costs. Occupational and Environmental Medicine. 2019;76:A22.1-A.

123. Benavides FG, Ramada JM, Ubalde-López M, Delclos GL, Serra C. A hospital occupational diseases unit: an experience to increase the recognition of occupational disease. Med Lav. 2019;110(4):278-84.

124. Lasfargues G, Jacquetin P, Vongmany N, Chauvet C, Durand-Moreau Q. O5E.1 Data on acknowledgment and costs of work-related mental diseases in france. Occupational and Environmental Medicine. 2019;76(Suppl 1):A49.

125. A. C. Occupational contact dermatitis in South America. Contact Dermatitis. 2018;79:13.

126. Chang H-H, Wu C-L, Wang J-D, Kuo Y-C. 639 Under-reported asbestos-related lung cancer in taiwan. Occupational and Environmental Medicine. 2018;75(Suppl 2):A452.

127. Won JU. 1723 Workers’ compensation and social security system and occupational injuries. Occupational and Environmental Medicine. 2018;75(Suppl 2):A13.

128. Modenese A, Korpinen L, Gobba F. 931 Occupational skin cancer in outdoor workers in italy: expected number vs cases recognised by the italian national compensation authority (inail). Occupational and Environmental Medicine. 2018;75(Suppl 2):A430.

129. Guo HR, Tanaka S, Halperin WE, Cameron LL. Back pain prevalence in US industry and estimates of lost workdays. Am J Public Health. 1999;89(7):1029-35.

130. Curti S, Sauni R, Spreeuwers D, de Schryver A, Valenty M, Rivière S, et al. 1710e Interventions to increase the reporting of occupational diseases by physicians. Occupational and Environmental Medicine. 2018;75(Suppl 2):A128.

131. Mansyur M. 1667f The strengthening of osh regulation in indonesia to improve the occupational diseases management2018. A302.2-A p.

132. P. HCPCC. Avoidance of under-reporting and selection bias in occupational injury and illness surveillance system. Occupational and Environmental Medicine. 2018;75:A143.

133. M.; Gilhooley E.; Boggs J.; Bourke J. FAMAMSRLDENMOOCRMSCLS. A multicentre audit of occupational dermatoses in the Republic of Ireland. British Journal of Dermatology. 2018;179:179.

134. Arnaud S, Viau A, Ferrer S, Iarmarcovai G, Saliba M-L, Souville M, et al. What barriers hinder the reporting of work related sciatic pain by GPs and rheumatologists? Santé publique (Vandoeuvre-lès-Nancy, France). 2008;20 Suppl 3:S39-48.

135. Drossard C, Lotz G. P294 Eu-wide data compilation of isocyanate-related occupational diseases. Occupational and Environmental Medicine. 2016;73(Suppl 1):A220.

136. Rappin CL, Wuellner SE, Bonauto DK. Employer reasons for failing to report eligible workers' compensation claims in the BLS survey of occupational injuries and illnesses. Am J Ind Med. 2016;59(5):343-56.

137. Wuellner SE, Adams DA, Bonauto DK. Unreported workers' compensation claims to the BLS Survey of Occupational Injuries and Illnesses: Establishment factors. Am J Ind Med. 2016;59(4):274-89.

138. Barber CM, Carder M, Agius R. P52 Epidemiology of Occupational Extrinsic Allergic Alveolitis reported to SWORD 1996–2014. Thorax. 2015;70(Suppl 3):A102.

139. M.M.-M. MC-LRSJM-MLJZGMLGEMDLP. Incidence of occupational respiratory diseases in. European Respiratory Journal. 2015;46.

140. Valenty M, Homère J, Lemaitre A, Plaine J, Ruhlman M, Cohidon C, et al. Surveillance programme for uncompensated work-related diseases in France. Occup Med (Lond). 2015;65(8):642-50.

141. Bhattacharya A. Costs of occupational musculoskeletal disorders (MSDs) in the United States. Int J Ind Ergon. 2014;44(3):448-54.

142. Thepaksorn P, Pongpanich S. Occupational injuries and illnesses and associated costs in Thailand. Saf Health Work. 2014;5(2):66-72.

143. Davis LK, Grattan KM, Tak S, Bullock LF, Ozonoff A, Boden LI. Use of multiple data sources for surveillance of work-related amputations in Massachusetts, comparison with official estimates and implications for national surveillance. Am J Ind Med. 2014;57(10):1120-32.

144. Barrick K, Levesque LE, Dostaler S, Pickett W, Liss G, Tarlo SM, et al. Estimating The Burden Of Work-Related Asthma Using Compensation And Administrative Data Linkage. B106 OCCUPATIONAL AND ENVIRONMENTAL DETERMINANTS OF LUNG FUNCTION. American Thoracic Society International Conference Abstracts: American Thoracic Society; 2013. p. A3664-A.

145. L. GKBLRYTSD. Using multiple data sources to enumerate workrelated amputations in massachusetts. American Journal of Epidemiology. 2013;177:S53.

146. Myong JP, Ahn YS, Kim HR, Kim YJ, Park CY, Koo JW. Work-related infectious diseases among Korean workers compensated under the Industrial Accident Compensation Insurance Law, 2006-2011. Int J Occup Environ Health. 2013;19(4):344-51.

147. Pelclova D, Fenclova Z, Urban P. Occupational cancer in the Czech Republic - a tip of the iceberg? European Journal of Oncology. 2011;16:149-61.

148. Rivière S, Chevalier A, Penven E, Cadéac-Birman H, Valenty M. Estimation of under-reporting of work-related musculoskeletal diseases (MSDs) in France: comparison between data from compensated occupational diseases system and data from epidemiological surveillance system of non-compensated work-related diseases (WRD). Occupational and Environmental Medicine. 2011;68(Suppl 1):A109.

149. Gisquet E, Chamming's S, Pairon JC, Gilg Soit Ilg A, Imbernon E, Goldberg M. [The determinants of under-reporting occupational diseases. The case of mesothelioma]. Rev Epidemiol Sante Publique. 2011;59(6):393-400.

150. Choi KS, Kang SK. Occupational psychiatric disorders in Korea. J Korean Med Sci. 2010;25(Suppl):S87-93.

151. García Gómez M, Castañeda López R. Desigualdades interterritoriales en la compensación de las enfermedades profesionales en España de 1990 a 2007. Gaceta Sanitaria. 2009;23(5):373-9.

152. Bislimovska J, Minov J, Mijakoski D, Stoleski S, Sasho T. Brucellosis as an Occupational Disease in the Republic of Macedonia. Macedonian Journal of Medical Sciences. 2010;3.

153. Bresciani M, Riva M, Giorgi M, Ghezzi L, Sidoti C, Mosconi G. [Diagnosis and insurance compensation of occupational diseases in construction industry]. Giornale italiano di medicina del lavoro ed ergonomia. 2007;29:611-3.

154. Verger P, Viau A, Arnaud S, Cabut S, Saliba ML, Iarmarcovai G, et al. Barriers to physician reporting of workers' compensation cases in France. Int J Occup Environ Health. 2008;14(3):198-205.

155. Mammone T, Metruccio F, Vida P, Moretto A. The Italian system of data reporting in agriculture occupational health: a critical appraisal. Journal of Public Health. 2007;15(4):301-13.

156. Emmert B, Hallier E. Ärztliche Meldepflicht bei begründetem Verdacht auf Vorliegen einer Berufskrankheit. Zeitschrift für Allgemeinmedizin. 2006;82(1):21-6.

157. Chen SY, Wang HF, Yin Y. [The reporting system of acute pesticides poisoning and general situation of pesticides poisoning in China]. Zhonghua Lao Dong Wei Sheng Zhi Ye Bing Za Zhi. 2005;23(5):336-9.

158. Axelopoulos CG, Rachiotis G, Valassi M, Drivas S, Behrakis P. Under-registration of occupational diseases: the Greek case. Occupational Medicine. 2005;55(1):64-5.

159. Morken T, Bråtveit M, Moen BE. [Reporting of occupational hearing loss in the Norwegian offshore industry 1992-2003]. Tidsskr Nor Laegeforen. 2005;125(23):3272-4.

160. Lezáun M. Intoxicaciones de origen laboral. Anales del Sistema Sanitario de Navarra. 2003;26:265-73.

161. Diepgen T, Schmidt A. Are the incidence and prevalence of occupational skin diseases underestimated? Arbeitsmedizin Sozialmedizin Umweltmedizin. 2002;37:477-80.

162. Pelclova D, Ameille J, Urban P, Fenclová Z, Lebedová J. Occupational diseases in the Czech Republic. 2001;62:629-33.

163. Pelclová D, Fenclová Z, Lebedová J. Occupational diseases in the Czech Republic in the year 1998. The need for unifying European standards/criteria for all occupational diseases. Cent Eur J Public Health. 2000;8(1):49-52.

164. L.L. GH-RTSHWEC. Back pain prevalence in US industry and estimates of lost workdays. American Journal of Public Health. 1999;89(7):1029-35.

165. Koton S, Ifrah A, Lerman Y, Ribak J, Green MS. Workers' health in Israel. Public Health Rev. 1998;26(2):189-203.

166. M.J.F. D. Reporting of occupational disease. British Medical Journal 1996;313(7065):1136-40.

167. Webster BS, Snook SH. The cost of 1989 workers' compensation low back pain claims. Spine (Phila Pa 1976). 1994;19(10):1111-5; discussion 6.

168. Gaffuri E. Disparity between estimated numbers and reported cases of occupational cancer. Scandinavian Journal of Work, Environment & Health. 1991(3):216-7.

169. P. NHGPT. 142 Cases of occupational poisoning with organic solvents reported to the Industrial Injuries Security Office in 1961-1970. I. Exposure. Ugeskrift for Laeger. 1979;141(38):2635-9.

170. M K. Incidence and significance of dermatologic occupational diseases. Zeitschrift fur Hautkrankheiten. 1974;49(21):923-7.

171. C. GRPGHB. Total number of compensated occupational diseases in France. Archives des maladies professionnelles de medecine du travail et de securite sociale. 1972;33(4):201-2.

172. G. I. Reporting and compensation procedures of occupational diseases and their statistical comprehension in the USA. Zentralblatt fur Arbeitsmedizin und Arbeitsschutz. 1966.

173. Lele DV. OCcupational Health Surveillance. Indian J Occup Environ Med. 2018;22(3):117-20.

174. Rautiainen RH, Ohsfeldt R, Sprince NL, Donham KJ, Burmeister LF, Reynolds SJ, et al. Cost of compensated injuries and occupational diseases in agriculture in Finland. J Agromedicine. 2005;10(3):21-9.

175. Islam SS, Doyle EJ, Velilla A, Martin CJ, Ducatman AM. Epidemiology of compensable work-related ocular injuries and illnesses: incidence and risk factors. J Occup Environ Med. 2000;42(6):575-81.

176. KL F-JGLRMLM. Work-related illness. Lung and skin disorders. Patient Care. 1994;28:48-58.

177. MZ LAOARKY. Reporting of occupational injury and occupational disease: current situation in Malaysia. Asian-Pacific Newsletter on Occupational Health & Safety. 2010;17:39-42.

178. LH S. Singapore’s Framework for Reporting

Occupational Accidents, Injuries and Diseases. Afr Newslett on Occup Health and Safety. 2010;20:57-60.

179. Goldberg M, Imbernon E, Rolland P, Gilg Soit Ilg A, Savès M, de Quillacq A, et al. The French National Mesothelioma Surveillance Program. Occup Environ Med. 2006;63(6):390-5.

180. Koehoorn M, Cole DC, Hertzman C, Lee H. Health care use associated with work-related musculoskeletal disorders among hospital workers. J Occup Rehabil. 2006;16(3):411-24.

181. Salim CAS. 151 Social Security in Brazil: the impact of epidemiological nexus on the benefits related to occupational diseases. Occupational and Environmental Medicine. 2013;70(Suppl 1):A51.

182. SK KEK. Reporting system for occupational injuries and illness in Korea. Asian-Pacific Newsletter on Occupational Health & Safety 2010;17:32-4.

183. M MJC-Y. Asthma in the Workplace: A Canadian Contribution and Perspective. Canadian Respiratory Journal. 2007;14:407-13.

184. T K. Improving reporting systems of occupational accidents and diseases. Asian-Pacific Newsletter on Occupational Health & Safety. 2010;17:27.

185. Hnizdo E, Rees D, Saader N. Surveillance of occupational respiratory diseases in South Africa. S Afr Med J. 1996;86(9):1127-8.

186. Barlet B, Prete G. La sous-déclaration et la sous-reconnaissance des maladies professionnelles en France (1990–2020) : revue de littérature pluridisciplinaire. Archives des Maladies Professionnelles et de l'Environnement. 2022;83(1):27-44.

187. Chen CY, Cheng Y. Occupational injuries and diseases in agricultural sector and worker' compensation for agricultural workers. Taiwan Journal of Public Health. 2018;37:237-43.

188. Ruser J. Examining Evidence on Whether BLS Undercounts Workplace Injuries and Illnesses. Mon Labor Rev. 2007;131.

189. Staniszewska MD, Pałczyński C, Hanke W. [Occupational respiratory diseases surveillance programs]. Med Pr. 2006;57(1):41-5.

190. Morse T, Dillon C, Warren N. Reporting of work-related musculoskeletal disorder (MSD) to workers compensation. New Solut. 2000;10(3):281-92.

191. Freund E, Seligman PJ, Chorba TL, Safford SK, Drachman JG, Hull HF. Mandatory reporting of occupational diseases by clinicians. Jama. 1989;262(21):3041-4.

192. CDC. Occupational Disease Surveillance: Occupational Asthma: Centers for Disease Control and Prevention; 1990 [updated 23 February 1990. Available from: <https://www.cdc.gov/MMWR/preview/mmwrhtml/00001565.htm>.

193. Muldoon JT, Wintermeyer LA, Eure JA, Fuortes L, Merchant JA, Van Lier SF, et al. Occupational disease surveillance data sources, 1985. Am J Public Health. 1987;77(8):1006-8.

194. Zhou AY, Seed M, Carder M, Money A, Van Tongeren M, Agius R. Sentinel approach to detect emerging causes of work-related respiratory diseases. Occup Med (Lond). 2020;70(1):52-9.

195. Ahn YS, Kim MG. Occupational skin diseases in Korea. J Korean Med Sci. 2010;25(Suppl):S46-52.

196. Mazurek JM, Filios M, Willis R, Rosenman KD, Reilly MJ, McGreevy K, et al. Work-related asthma in the educational services industry: California, Massachusetts, Michigan, and New Jersey, 1993-2000. Am J Ind Med. 2008;51(1):47-59.

197. MC TKB. Occupational cancer in Canada: what do we know? CMAJ. 1992;147:1501-7.

198. Peyrethon C. Under-reporting and under-recognition of occupational diseases in France A review. Archives des Maladies Professionnelles et de l'Environnement. 2022;83:45-6.

199. Brunier S, Jouzel J-N, Prete G. Healing the living rather than righting the wrongs: The organization of hospital medicine and the underreporting of occupational hemopathies. Sciences sociales et santé. 2022;Vol. 40(1):5-30.

200. Rachiotis G, Alexopoulos CG, Symvoulakis EK, Hadjichristodoulou C, Drivas S. Features of registered occupational diseases in Greece: a veil of ignorance. Med Lav. 2012;103(3):230-5.

201. Ndiaye M, Niang T, Soumah MM, Dia SA, Fall MCG, Sow ML. Occupational diseases in Senegal: current situation and perspectives. Archives des Maladies Professionnelles et de l’Environnement. 2014;75(6):584–9.

202. Dickel H, Bruckner T, Bernhard-Klimt C, Koch T, Scheidt R, Diepgen TL. Surveillance scheme for occupational skin disease in the Saarland, FRG. First report from BKH-S. Contact Dermatitis. 2002;46(4):197-206.

203. Hagemeyer O, Butz M, Otten H. [Ratio of confirmed versus suspected occupational diseases as a parameter of quality]. Gesundheitswesen. 2005;67(3):189-95.

204. Nilson L, Herloff B, Thiringer G. Mental and psychosomatic work injuries in Sweden. J Occup Med. 1992;34(7):726-30.

205. Stolovas N, Pintos J, Luaces ME, Pérez Lorenzo C, De Ben S, Tomasina F. Mesotelioma y ocupación. Revisión de casos en Uruguay: 2002-2014. Anales de la Facultad de Medicina. 2021;8(2):e202.

206. Stephenson J. Work-Related Illness, Injury. JAMA. 2005;294(15):1890-.

207. Cavalli LS, Marques FB, Watterson A. A critical overview of work-related injury and illness in aquaculture workers from Brazil. Reviews in Aquaculture. 2020;12(2):1157-64.

208. Fan X, Straube S. Reporting on work-related low back pain: data sources, discrepancies and the art of discovering truths. Pain Manag. 2016;6(6):553-9.

209. LaSee CR, Reeb-Whitaker CK. Work-related asthma surveillance in Washington State: time trends, industry rates, and workers' compensation costs, 2002-2016. J Asthma. 2020;57(4):421-30.

210. Spreeuwers D, de Boer AGEM, Verbeek JHAM, van Dijk FJH. Characteristics of national registries for occupational diseases: international development and validation of an audit tool (ODIT). BMC Health Services Research. 2009;9(1):194.

211. Lenderink AF, Spreeuwers D, van der Klink JJ, van Dijk FJ. Information and feedback to improve occupational physicians' reporting of occupational diseases: a randomised controlled trial. Int Arch Occup Environ Health. 2010;83(4):381-8.

212. Menzel NN. Underreporting of musculoskeletal disorders among health care workers: research needs. Aaohn j. 2008;56(12):487-94.

213. Chaiklieng S, Chagkornburee C, Suggaravetsiri P. Situations of work-related diseases and injuries among agriculturists in the upper northeast regions of Thailand. F1000Res. 2022;11:145.

214. Wang CC, Lin GL, Lin YJ, Chen WL, Wu WT. Occupational health surveillance and detection of emerging occupational diseases among Taiwan farmers, through analysis of national-based farmers' and medico-administrative databases. Am J Ind Med. 2023;66(1):85-93.

215. Baur X, Degens P, Weber K. Occupational obstructive airway diseases in Germany. Am J Ind Med. 1998;33(5):454-62.

216. Kathare M, Julander A, Erfani B, Schenk L. An Overview of Cleaning Agents' Health Hazards and Occupational Injuries and Diseases Attributed to Them in Sweden. Ann Work Expo Health. 2022;66(6):741-53.

217. Yildiz AN, Piskin TM, Alaguney ME, Kurt OK, Ozlu A, Basarali MK. Attitudes and behaviors of family physicians regarding occupational diseases. Arch Environ Occup Health. 2019;74(1-2):85-92.

218. Davoodi S, Haghighi KS, Kalhori SRN, Hosseini NS, Mohammadzadeh Z, Safdari R. Occupational Disease Registries-Characteristics and Experiences. Acta Inform Med. 2017;25(2):136-40.

219. Keegel T, Moyle M, Dharmage S, Frowen K, Nixon R. The epidemiology of occupational contact dermatitis (1990-2007): a systematic review. Int J Dermatol. 2009;48(6):571-8.

220. Anderson NJ, Reeb-Whitaker CK, Bonauto DK, Rauser E. Work-related asthma in Washington State. J Asthma. 2011;48(8):773-82.

221. Murphy PL, Sorock GS, Courtney TK, Webster BS, Leamon TB. Injury and illness in the American workplace: a comparison of data sources. Am J Ind Med. 1996;30(2):130-41.

222. Piedrahita H. Costs of work-related musculoskeletal disorders (MSDs) in developing countries: Colombia case. Int J Occup Saf Ergon. 2006;12(4):379-86.

223. Hämäläinen PH, Saarela KL, Takala J. Global estimates of fatal work-related diseases by region and disease group, 2002. Int J Occup Environ Health. 2011;17(1):49-56.

224. Kirkham TL, Koehoorn MW, McLeod CB, Demers PA. Surveillance of mesothelioma and workers&#039; compensation in British Columbia, Canada. Occupational and Environmental Medicine. 2011;68(1):30.

225. Oyunbileg S, Sumberzul N, Oyuntogos L, Javzmaa J, Wang JD. Analysis of incidence rates of occupational diseases in Mongolia, 1986-2006. Int J Occup Environ Health. 2011;17(1):31-7.

226. Larese Filon F, Pesce M, Paulo MS, Loney T, Modenese A, John SM, et al. Incidence of occupational contact dermatitis in healthcare workers: a systematic review. J Eur Acad Dermatol Venereol. 2021;35(6):1285-9.

227. Cohen RA, Go LHT, Rose CS. Global Trends in Occupational Lung Disease. Semin Respir Crit Care Med. 2023;44(3):317-26.

228. Tanaka S, Wild DK, Seligman PJ, Halperin WE, Behrens VJ, Putz-Anderson V. Prevalence and work-relatedness of self-reported carpal tunnel syndrome among U.S. workers: analysis of the Occupational Health Supplement data of 1988 National Health Interview Survey. Am J Ind Med. 1995;27(4):451-70.

229. Anderson NJ, Bonauto DK, Adams D. Work-related amputations in Washington state, 1997-2005. Am J Ind Med. 2010;53(7):693-705.

230. Waclawski E. Disease reporting after the Reporting of Injuries, Diseases, and Dangerous Occurrence Regulations (1995) (RIDDOR) is revised. Occup Med (Lond). 2013;63(3):168-9.

231. Muula AS, Rudatsikira E, Siziya S. Occupational illnesses in the 2009 Zambian labour force survey. BMC Res Notes. 2010;3:272.

232. Shi DS, Weaver VM, Hodgson MJ, Tustin AW. Hospitalised heat-related acute kidney injury in indoor and outdoor workers in the USA. Occup Environ Med. 2022;79(3):184-91.

233. Zhang X, Wang Z, Li T. The current status of occupational health in China. Environ Health Prev Med. 2010;15(5):263-70.

234. Gerr F. Surveillance of work-related musculoskeletal disorders. Occupational and Environmental Medicine. 2008;65(5):298.

235. Zhou L, Wei F, Fang X, Zhang Y, Hu Y, Lou X, et al. Epidemiological characteristics of occupational chemical poisonings in Zhejiang, China from 2006 to 2020: A descriptive analysis. Frontiers in Public Health. 2022;Volume 10 - 2022.

236. Largo TW, Rosenman KD. Michigan work-related amputations, 2008. J Occup Environ Med. 2013;55(3):280-5.

237. Liss GMT, S M. Work related asthma. Occupational and Environmental Medicine. 2002;59:503.

238. Thuerauf JR. Notification of adverse health effects due to chemicals: two different ways in Germany. Int Arch Occup Environ Health. 1996;68(6):436-41.

239. Del Bianco A, Demers PA. Trends in compensation for deaths from occupational cancer in Canada: a descriptive study. CMAJ Open. 2013;1(3):E91-6.

240. Masterson EA, Deddens JA, Themann CL, Bertke S, Calvert GM. Trends in worker hearing loss by industry sector, 1981-2010. Am J Ind Med. 2015;58(4):392-401.

241. Volinn E, Nishikitani M, Volinn W, Nakamura Y, Yano E. Back pain claim rates in Japan and the United States: framing the puzzle. Spine (Phila Pa 1976). 2005;30(6):697-704.

242. Hung CL, Su PL, Ou CY. Prognostic effect of tuberculosis on patients with occupational lung diseases: A 13-year observational study in a nationwide cohort. Medicine (Baltimore). 2016;95(37):e4748.

243. Nienhaus A, Hod R. COVID-19 among Health Workers in Germany and Malaysia. Int J Environ Res Public Health. 2020;17(13).

244. Marinaccio A, Binazzi A, Bonafede M, Altimari A, Boscioni R, Corfiati M, et al. Occupational diseases in Italian national priority contaminated sites. Am J Ind Med. 2018;61(7):582-91.

245. Almberg KS, Halldin CN, Blackley DJ, Laney AS, Storey E, Rose CS, et al. Progressive Massive Fibrosis Resurgence Identified in U.S. Coal Miners Filing for Black Lung Benefits, 1970-2016. Ann Am Thorac Soc. 2018;15(12):1420-6.

246. García-Gómez M, Menéndez-Navarro A, López RC. Asbestos-related occupational cancers compensated under the Spanish National Insurance System, 1978-2011. Int J Occup Environ Health. 2015;21(1):31-9.

247. Binazzi A, Corfiati M, Di Marzio D, Cacciatore AM, Zajacovà J, Mensi C, et al. Sinonasal cancer in the Italian national surveillance system: Epidemiology, occupation, and public health implications. Am J Ind Med. 2018;61(3):239-50.

248. Lehnert M, Kraywinkel K, Heinze E, Wiethege T, Johnen G, Fiebig J, et al. Incidence of malignant mesothelioma in Germany 2009-2013. Cancer Causes Control. 2017;28(2):97-105.

249. Pickard O, Burton P, Yamada H, Schram B, Canetti EFD, Orr R. Musculoskeletal Disorders Associated with Occupational Driving: A Systematic Review Spanning 2006-2021. Int J Environ Res Public Health. 2022;19(11).

250. Szeszenia-Dąbrowska N, Wilczyńska U. Choroby zawodowe wśród pracujących w różnych gałęziach gospodarki narodowej. Medycyna Pracy Workers' Health and Safety. 2013;64(2):161-74.

251. Smith L, Westrick R, Sauers S, Cooper A, Scofield D, Claro P, et al. Underreporting of Musculoskeletal Injuries in the US Army: Findings From an Infantry Brigade Combat Team Survey Study. Sports Health. 2016;8(6):507-13.

252. Lee K, Lee S, Min J, Kim I. Occupational cancer claims in Korea from 2010 to 2016. Ann Occup Environ Med. 2018;30:64.

253. da Costa JT, Baptista JS, Vaz M. Incidence and prevalence of upper-limb work related musculoskeletal disorders: A systematic review. Work. 2015;51(4):635-44.

254. Marinaccio A, Binazzi A, Marzio D, Massari S, Scarselli A, Iavicoli S. [The contribution of surveillance systems of occupational diseases and mesothelioma in environmental health studies]. Epidemiologia e prevenzione. 2011;35:185-8.

255. Fishwick D, Carder M, Iskandar I, Fishwick BC, van Tongeren M. Occupational inhalational accidents: analysis of cases from the UK SWORD reporting scheme from 1999 to 2018. Occup Environ Med. 2022.

256. Lytras T, Danis K, Dounias G. Incidence Patterns and Occupational Risk Factors of Human Brucellosis in Greece, 2004-2015. Int J Occup Environ Med. 2016;7(4):221-6.

257. Frazier LM, Jones B, Darcey D, Langley R, Randolph S. Mandatory reporting of occupational health problems. A new surveillance program in North Carolina. N C Med J. 1994;55(11):526-31.

258. Takala J, Hämäläinen P, Saarela KL, Yun LY, Manickam K, Jin TW, et al. Global estimates of the burden of injury and illness at work in 2012. J Occup Environ Hyg. 2014;11(5):326-37.

259. Salo SAS, Ilonen I, Laaksonen S, Myllärniemi M, Salo JA, Rantanen T. Epidemiology of malignant peritoneal mesothelioma: A population-based study. Cancer Epidemiol. 2017;51:81-6.

260. Shi P, Xing X, Xi S, Jing H, Yuan J, Fu Z, et al. Trends in global, regional and national incidence of pneumoconiosis caused by different aetiologies: an analysis from the Global Burden of Disease Study 2017. Occup Environ Med. 2020;77(6):407-14.

261. Kurth L, Casey ML, Mazurek JM, Blackley DJ. Pneumoconiosis incidence and prevalence among US Medicare beneficiaries, 1999-2019. Am J Ind Med. 2023;66(10):831-41.

262. Rosenman KD, Fussman C. Prevalence of work-related dermatitis in the working population. American Journal of Industrial Medicine. 2014;57(1):125-6.

263. Tarlo SM. Trends in incidence of occupational asthma. Occup Environ Med. 2015;72(10):688-9.

264. Kauppinen T, Uuksulainen S, Saalo A, Mäkinen I. Trends of occupational exposure to chemical agents in Finland in 1950-2020. Ann Occup Hyg. 2013;57(5):593-609.

265. Karjalainen A, Martikainen R, Klaukka T, Saarinen K, Uitti J. Risk of asthma among Finnish patients with occupational rhinitis. Chest. 2003;123(1):283-8.

266. Kang DM, Kim JE, Kim YK, Lee HH, Kim SY. Occupational Burden of Asbestos-Related Diseases in Korea, 1998-2013: Asbestosis, Mesothelioma, Lung Cancer, Laryngeal Cancer, and Ovarian Cancer. J Korean Med Sci. 2018;33(35):e226.

267. Malo JL, Chan-Yeung M. Asthma in the workplace: a Canadian contribution and perspective. Can Respir J. 2007;14(7):407-13.

268. von Hirschberg KR, Kähler B, Nienhaus A. Social care and changes in occupational accidents and diseases - the situation in Eastern Europe in general and for skin diseases in particular. J Occup Med Toxicol. 2009;4:28.

269. Rosenman KD, Reilly MJ, Kalinowski DJ. A State-Based Surveillance System For Work-Related Asthma. Journal of Occupational and Environmental Medicine. 1997;39(5).

270. Draper A, Newman Taylor A, Cullinan P. Estimating the incidence of occupational asthma and rhinitis from laboratory animal allergens in the UK, 1999-2000. Occup Environ Med. 2003;60(8):604-5.

271. Siddharthan K, Hodgson M, Rosenberg D, Haiduven D, Nelson A. Under-reporting of work-related musculoskeletal disorders in the Veterans Administration. Int J Health Care Qual Assur Inc Leadersh Health Serv. 2006;19(6-7):463-76.

272. Loeppke R, Heron R, Bazas T, Beaumont D, Spanjaard H, Konicki DL, et al. Global Trends in Occupational Medicine: Results of the International Occupational Medicine Society Collaborative Survey. J Occup Environ Med. 2017;59(3):e13-e6.

273. Spence A Fau - Hodgson J, Hodgson J Fau - Osman J, Osman J Fau - Cockcroft A, Cockcroft A. Monitoring occupational diseases: response. Occup Environ Med. 2001;58(11):692-3.

274. Govaerts R, Tassignon B, Ghillebert J, Serrien B, De Bock S, Ampe T, et al. Prevalence and incidence of work-related musculoskeletal disorders in secondary industries of 21st century Europe: a systematic review and meta-analysis. BMC Musculoskelet Disord. 2021;22(1):751.

275. Vargas-Prada S. Work-related musculoskeletal disorders: are they truly declining? Occup Environ Med. 2015;72(4):239-40.

276. Mengistu DA, Mulugeta Demmu Y, Alemu A. Occupational Related Upper and Low Back Pain Among the Working Population of Ethiopia: Systematic Review and Meta-Analysis. Environ Health Insights. 2021;15:11786302211067839.

277. Nanyan P, Ben Charrada M. Compensation claims for work-related musculoskeletal disorders among hairdressers in France, 2010-2016. Int J Occup Saf Ergon. 2020;26(4):824-8.

278. van der Molen HF, Kezic S, Visser S, de Groene G, Maas J, de Wind A, et al. Occupational COVID-19: what can be learned from notifications of occupational diseases? Occup Environ Med. 2020;78(6):464.

279. DH ALLCW. Occupational injury and illness surveillance: conceptual filters explain underreporting. Am J Public Health. 2002;92:1421-9.

280. F OC. Evaluation of Underreporting of Work-Related Injuries and Illnesses in the United States

Military: East Carolina University; 2020.

281. A SKHMRDHDN. Under‐reporting of work‐related musculoskeletal disorders in the Veterans Administration. International Journal of Health Care Quality Assurance. 2006;19:463-76.

282. Min J, Kim EM, Kim J, Jang J, Choi Y, Kim I. Data profile: the Korean Workers' Compensation-National Health Insurance Service (KoWorC-NHIS) cohort. Epidemiol Health. 2024;46:e2024071.

283. ZHU TXYMX. Overview of work-related diseases and occupational injury surveillance system in the UK. Journal of Environmental and Occupational Medicine. 2023;12:1170-4.

284. Myers NT; Dodd KE; Hale JM BDHN. Acute Occupational Respiratory Injuries and Fatalities, United States, 2016-2020. Am J Respir Crit Care Med. 2023;207:A4355.

285. Mongeau S, Lightfoot N, Donato E, Eger T. Administrative Burden of the Compensation Claim System - Physicians and Union Compensation Representatives’ Views. Diversity of Research in Health Journal. 2023;6:1-15.

286. Chen PH, Chu PC, Huang CC, Chen CH, Guo YL, Su TC, et al. Medical accessibility and underreporting of occupational diseases: effect of travel distance and travel time. Front Rehabil Sci. 2025;6:1545460.

287. Brans R. Epidemiology and Burden of Occupational Skin Diseases. In: Giménez-Arnau AM, Maibach HI, editors. Handbook of Occupational Dermatoses. Cham: Springer International Publishing; 2023. p. 13-21.

288. Strømholm T, Samant Y, Stette-Haarberg I, Støver M, Lohmann-Lafrenz S. O-169 WORK-RELATED COVID-19 CASES REPORTED TO THE NORWEGIAN LABOUR INSPECTION AUTHORITY. Occupational Medicine. 2024;74(Supplement_1):0-.
