# Supplementary material for: Global reporting and underreporting of occupational diseases: A systematic review
Source: PLoS One. 2026 Mar 26;21(3):e0345318. doi: 10.1371/journal.pone.0345318 (PMC13020801; doi:10.1371/journal.pone.0345318)
Supplement: S13 File — (DOCX) [file pone.0345318.s013.docx]

**S13. Risk of bias assessment of quantitative non-randomized studies using Mixed Method Appraisal Tool (MMAT) version 2018**

| No | Author, year | 1. Are the participants representative of the target population? | 2. Are measurements appropriate regarding both the outcome and intervention (or exposure)? | 3. Are there complete outcome data? | 4. Are the confounders accounted for in the design and analysis? | 5. During the study period, is the intervention administered (or exposure occurred) as intended? | Total score |
| --- | --- | --- | --- | --- | --- | --- | --- |
| 1 | Parhar, 2011(1) | Yes | Can’t tell | Yes | Can’t tell | Yes | 60 |
| 2 | Arnaud, 2010(2) | Yes | Yes | Yes | No | Yes | 80 |

1. Parhar A, Lemiere C, Beach JR. Barriers to the recognition and reporting of occupational asthma by Canadian pulmonologists. Can Respir J. 2011;18(2):90-6.

2. Arnaud S, Cabut S, Viau A, Souville M, Verger P. Different reporting patterns for occupational diseases among physicians: a study of French general practitioners, pulmonologists and rheumatologists. Int Arch Occup Environ Health. 2010;83(3):251-8.
