# Supplementary material for: Global reporting and underreporting of occupational diseases: A systematic review
Source: PLoS One. 2026 Mar 26;21(3):e0345318. doi: 10.1371/journal.pone.0345318 (PMC13020801; doi:10.1371/journal.pone.0345318)
Supplement: S14 File — (DOCX) [file pone.0345318.s014.docx]

**S14. Risk of bias assessment of qualitative studies using Mixed Method Appraisal Tool (MMAT) version 2018**

| No | Author, year | 1. Is the qualitative approach appropriate to answer the research question? | 2. Are the qualitative data collection methods adequate to address the research question? | 3. Are the findings adequately derived from the data? | 4. Is the interpretation of results sufficiently substantiated by data? | 5. Is there coherence between qualitative data sources, collection, analysis and interpretation? | Total score |
| --- | --- | --- | --- | --- | --- | --- | --- |
| 1 | Ding, 2013(1) | Yes | Yes | Yes | Yes | Yes | 100 |
| 2 | Fagan, 2016(2) | Yes | Yes | No | Yes | Yes | 80 |
| 3 | Cheng, 2022(3) | Yes | Yes | Yes | Yes | Yes | 100 |
| 4 | Karabağ, 2023(4) | Yes | Can’t tell | Yes | Yes | Yes | 80 |

1. Ding Q SL, Hansson SO. Occupational diseases in the People’s Republic of China between 2000 and 2010. American Journal of Industrial Medicine. 2013;56(12):1423-32.

2. Fagan KM, Hodgson MJ. Under-recording of work-related injuries and illnesses: An OSHA priority. J Safety Res. 2017;60:79-83.

3. Cheng Y, Huang YL, Lee LJ. Explaining the Invisibility of Asbestos-Related Diseases in the Taiwan Workers' Compensation System. New Solut. 2022;32(2):106-18.

4. Karabağ İ, Alagüney ME, Şahan C, Yıldız AN. How difficult is it to diagnose and report an occupational disease in a developing country? A modified delphi study. Acta Medica. 2023;54(4):347-56.
