# Supplementary material for: Global reporting and underreporting of occupational diseases: A systematic review
Source: PLoS One. 2026 Mar 26;21(3):e0345318. doi: 10.1371/journal.pone.0345318 (PMC13020801; doi:10.1371/journal.pone.0345318)
Supplement: S15 File — (DOCX) [file pone.0345318.s015.docx]

**PRISMA 2020 Checklist**

| **Checklist item** | **Location where item is reported** |
| --- | --- |
| Item 1. TITLE: Identify the report as a systematic review. | The authors identify the report as a systematic review as shown in the title: “Global reporting and underreporting of occupational disease: a systematic review”. |
| Item 2. ABSTRACT: See the PRISMA 2020 for Abstracts checklist. | In the abstract, the authors summarise the objective, databases searched, eligibility criteria, methods for selecting studies, assessing the risk of bias and synthesizing results, along with presenting the results and specify the registration number for the review. “**Background** Disease reporting is often unreliable and faces many challenges, making it difficult to estimate the true burden of occupational diseases, defined as any disease that is caused by work activities or environment. This study aimed to assess the global reporting and underreporting rate of occupational diseases, and to identify the factors affecting the underreporting of occupational diseases. **Methods** Following the Preferred Reporting Items for Systematic Reviews and Meta-Analysis (PRISMA) guidelines, this study searched Medline (PubMed), CINAHL, EMBASE, Scopus, Web of Science, WHO Institutional Repository for Information Sharing (IRIS) database, Dimensions, and Google Scholar in September 2024. Search terms related to reporting and underreporting of occupational diseases or illnesses were used. The selected records were screened, and data extracted using the Covidence software tool. Screening and quality assessment were conducted by two independent researchers and finalized by a third researcher. The quality of the evidence was assessed with the Mixed Methods Appraisal Tool. This study is registered on PROSPERO, number CRD42023417814. **Results** A total of 127 studies from 29 countries were identified, all coming from high-income and upper-middle-income countries. The incidence rate of occupational disease varied widely, ranging between 1.71 to 1,387 per 100,000 employees yearly. The highest number of annual cases was reported in the agricultural sector (ranging from 33 to 6,431), followed by the health sector (146 to 5,508), and then the construction sector (264). Two studies evaluated rates of underreporting, which varied from 50% to 95%. The main factor contributing to underreporting was employee concerns about job security. **Conclusions** The results reveal a significant gap in the reporting of occupational diseases among high-income and low-middle-income countries. Variations in reporting mechanisms across countries were also identified. Our findings highlight the need to establish a national system for reporting occupational diseases that engages employers, employees, and healthcare providers.” |
| Item 3. RATIONALE: Describe the rationale for the review in the context of existing knowledge. | The authors the knowledge gap of the existing evidence base and report no previous review addressing our research questions exists.  “While studies have documented the burden of occupational diseases, there has been limited effort to explore the global disease pattern and identify its reporting mechanisms. Underreporting of occupational diseases limits our understanding of the true burden of these diseases, leading to misallocation of resources and potentially ineffective prevention efforts. To our knowledge, no systematic review exists on the global reporting of occupational diseases. This systematic review aims to fill that gap by bringing together evidence on the reporting of occupational diseases in all countries.” |
| Item 4. OBJECTIVES: Provide an explicit statement of the objective(s) or question(s) the review addresses. | The authors report the primary and secondary objectives of this review.  “Our goal is to systematically review the reporting and underreporting pattern of occupational diseases based on countries’ income status, industrial development, and types of occupational diseases. We also aim to identify the factors affecting the underreporting of occupational diseases.” |
| Item 5. ELIGIBILITY CRITERIA: Specify the inclusion and exclusion criteria for the review and how studies were grouped for the syntheses. | The authors report the inclusion and exclusion criteria for type of studies, outcomes, date of publication, and language of publication.  “Studies were eligible for inclusion if they examined the reporting or underreporting of occupational diseases at the country level. Eligible studies included cohort studies, cross-sectional studies or reports from national registries, national surveillance systems, workers’ compensation schemes, or national voluntary reporting schemes. As this review focused on the reporting system at the national level, studies at the district or province level were excluded. We included studies that provide real world data and excluded purely modeling studies. All types of occupational diseases were included; however, occupational injuries or accidents were excluded. Review articles, editorials, guidelines, case reports, and case series were also excluded, as our focus was on empirical studies. Qualitative and mixed-methods studies were included to identify the factors underlying problems of underreporting. There were no limitations on the date of publications and language. For non-English language articles, Google Translate was used to screen titles and abstracts. These articles were kept in a separate folder but not included in the analysis, to avoid language bias when appraising the articles.” |
| Item 6. INFORMATION SOURCES: Specify all databases, registers, websites, organisations, reference lists and other sources searched or consulted to identify studies. Specify the date when each source was last searched or consulted. | The authors report the electronic bibliographic databases and other sources used. The authors also indicated the date when each was searched along with searching strategies in the S1. Appendix.  “Searches were conducted for the following electronic databases: Medline (PubMed), CINAHL, EMBASE, Scopus, and Web of Science. Searches for eligible grey literature were carried out in the WHO Institutional Repository for Information Sharing (IRIS) database, Dimensions, and Google. Additionally, the reference lists of relevant articles were screened for titles and abstracts that include key terms.” |
| Item 7. SEARCH STRATEGY: Present the full search strategies for all databases, registers and websites, including any filters and limits used. | The authors report the search strategies for each database in S1.File.  “We explored different possible terms related to reporting and underreporting of occupational diseases/illnesses including “report*”, “underreport*”, “misreport*”, “surveillance”, and “capture-recapture”, and combined them with terms for occupational diseases (“occupational disease*”, “occupational illness*”, “work-related disease”, “work-related illness”). The search strategy included a combination of Medical Subject Heading (MESH) terms and free text terms. These terms were combined with ‘OR’ and ‘AND’ Boolean operators. The full search strategy is provided in S1 File.” |
| Item 8. SELECTION PROCESS: Specify the methods used to decide whether a study met the inclusion criteria of the review, including how many reviewers screened each record and each report retrieved, whether they worked independently, and if applicable, details of automation tools used in the process. | The authors report the screening process, consensus methods, and the use of Covidence in the study selection process.  “Studies obtained from different data sources were combined and duplicate records were removed using the Covidence systematic review software package. All records identified in the search were initially screened based on titles and abstracts. Then, we assessed the full text of selected studies according to the eligibility criteria. Study selection was performed by two independent investigators (LK and SR or LK and MI). Any disagreements were resolved through discussion with a third reviewer (SR or MI). The third reviewer was a person not involved in the study selection process.” |
| Item 9. DATA COLLECTION PROCESS: Specify the methods used to collect data from reports, including how many reviewers collected data from each report, whether they worked independently, any processes for obtaining or confirming data from study investigators, and if applicable, details of automation tools used in the process. | The authors report using a data extraction form and indicate that independent reviewer extracted data, which was checked by another reviewer.  “A standardized form was developed for data extraction based on the review questions. Extracted information included basic study details such as study design, country of origin, study setting, year or timeframe for data collection, participant employment characteristics (industrial sector, job type), and the outcome data (e.g., number of cases reported, reporting rate). Additionally, the countries where the studies were conducted were stratified by income status and region according to the World Bank classification. One reviewer (LK) extracted the data, while a second reviewer (SR or MI) checked the data for accuracy and completeness. Any discrepancies between reviewers were resolved through consensus, or by involving a third reviewer (SR or MI) to settle the disagreement in the data extraction process.” |
| Item 10a. DATA ITEMS: List and define all outcomes for which data were sought. Specify whether all results that were compatible with each outcome domain in each study were sought (e.g. for all measures, time points, analyses), and if not, the methods used to decide which results to collect. | The authors list and define all possible outcomes for which data were sought.  “Outcome measures include the number of reported occupational disease cases, the rate of reporting occupational diseases, the rate of underreporting or misreporting, the number of occupational disease claims, the number of cases reported by physicians, employees or employers. The underreporting rate is defined as the ratio between the number of non-reported cases and the total number of cases (reported and not reported). The rate of misreporting refers to the ratio between the number of falsely reported cases and the total number of cases. Reported cases were classified according to the countries’ income status, industrial development, and types of occupational diseases.” |
| Item 10b. DATA ITEMS: List and define all other variables for which data were sought (e.g. participant and intervention characteristics, funding sources). Describe any assumptions made about any missing or unclear information. | The authors list other variables that included in the extracted information.  “Extracted information included basic study details such as study design, country of origin, study setting, year or timeframe for data collection, participant employment characteristics (industrial sector, job type), and the outcome data (e.g., number of cases reported, reporting rate). Additionally, the countries where the studies were conducted were stratified by income status and region according to the World Bank classification.” |
| Item 11. STUDY RISK OF BIAS ASSESSMENT: Specify the methods used to assess risk of bias in the included studies, including details of the tool(s) used, how many reviewers assessed each study and whether they worked independently, and if applicable, details of automation tools used in the process. | The authors specify the tool used to assess the quality of evidence, how each study was rated, and how an overall judgment was reached.  “At least two reviewers (LK and SR or LK and MI) assessed and appraised the methodological quality of the studies independently using Mixed Methods Appraisal Tool (MMAT) (26). The types of studies assessed using MMAT include qualitative studies, quantitative randomized controlled trials, quantitative non-randomized studies, quantitative descriptive studies, and mixed-method studies. Five criteria were used to assess the overall risk of bias for each study type. Each criterion is given a rating of ‘yes’, ‘no’, or ‘can’t tell’. For every ‘yes’ answer, the study was given a score of between 20 and 100, with 20 being the lowest and 100 the highest. If different results were obtained, the assigned investigators discussed until an agreement was reached. If no agreement was reached, a third investigator (SR or MI) was consulted to obtain an agreement.” |
| Item 12. EFFECT MEASURES: Specify for each outcome the effect measure(s) (e.g. risk ratio, mean difference) used in the synthesis or presentation of results. | The authors report the measures and how to calculate the annual case for each type of occupational disease.  “A summary table showing the number of cases per year and incidence of occupational diseases per 100,000 employees was presented. Where the incidence was originally reported using a different denominator (e.g., per one million or one thousand employees), the number was adjusted to a denominator of per 100,000 employees. The results were also classified based on the type of occupational diseases in each region, and the type of occupational diseases in each industrial sector. In every study, we calculated the number of annual cases for each type of occupational disease by adding up the total cases and dividing by the total number of years covered by the study to obtain the average number of cases per year. The average number of cases per year in each study were summed up and then divided by the total number of studies. For studies discussing the factors contributing to the underreporting of occupational diseases, these articles were separated into a separate folder and analysed thematically.” |
| Item 13a. SYNTHESIS METHODS: Describe the processes used to decide which studies were eligible for each synthesis (e.g. tabulating the study intervention characteristics and comparing against the planned groups for each synthesis (item #5)). | The authors report categorizing the studies based on their content, e.g., number of reported cases, incidence rate of occupational diseases.  “The articles were categorized based on their content. Articles that included the number of reported cases or incidence rate of occupational diseases were grouped into one folder and analysed to address the primary objective, i.e., to estimate the global reporting and underreporting rate of occupational diseases. Descriptive data from each reviewed study were presented as narrative text or in tables.” |
| Item 13b. SYNTHESIS METHODS: Describe any methods required to prepare the data for presentation or synthesis, such as handling of missing summary statistics, or data conversions. | The authors report conducting meta-analysis was not possible, thus, a narrative synthesis was preferred.  “Due to the diversity in the characteristics of the studies, conducting a meta-analysis was not possible. Thus, a narrative synthesis was performed for this systematic review.” |
| Item 13c. SYNTHESIS METHODS: Describe any methods used to tabulate or visually display results of individual studies and syntheses. | The authors report presenting the result based on the study objectives, type of occupational disease, and type of industrial sector. The categorization along with the number of studies in each category is presented in Figure 2.  “The remaining results are presented according to the study objectives, type of occupational disease, and type of industrial sector. We separated those articles to be analysed for the primary objective, i.e., to estimate the global reporting and underreporting rate of occupational diseases, and the secondary objective of identifying the factors contributing to the underreporting of occupational diseases. Eight articles were assessed for the secondary objective, and the rest were analysed for the primary objective. One article was assessed for both objectives. Of the 119 articles assessed for the primary objective, only two examined underreporting patterns in occupational diseases. Ninety-three (93) of the 119 studies examined only a specific occupational disease without considering the type of industrial sector. Meanwhile, there were 15 articles which discussed all types of occupational diseases among the general working population.” |
| Item 13d. SYNTHESIS METHODS: Describe any methods used to synthesize results and provide a rationale for the choice(s). If meta-analysis was performed, describe the model(s), method(s) to identify the presence and extent of statistical heterogeneity, and software package(s) used. | The authors report conducting meta-analysis was not possible. The calculation of number of annual cases by type of occupational disease was explained in the method section.  “In every study, we calculated the number of annual cases for each type of occupational disease by adding up the total cases and dividing by the total number of years covered by the study to obtain the average number of cases per year. The average number of cases per year in each study were summed up and then divided by the total number of studies.” |
| Item 13e. SYNTHESIS METHODS: Describe any methods used to explore possible causes of heterogeneity among study results (e.g. subgroup analysis, meta-regression). | The authors report conducting meta-analysis was not possible, thus, a narrative synthesis was preferred.  “Due to the diversity in the characteristics of the studies, conducting a meta-analysis was not possible. Thus, a narrative synthesis was performed for this systematic review.” |
| Item 13f. SYNTHESIS METHODS: Describe any sensitivity analyses conducted to assess robustness of the synthesized results. | The authors report conducting meta-analysis was not possible, thus, a narrative synthesis was preferred.  “Due to the diversity in the characteristics of the studies, conducting a meta-analysis was not possible. Thus, a narrative synthesis was performed for this systematic review.” |
| Item 14. REPORTING BIAS ASSESSMENT: Describe any methods used to assess risk of bias due to missing results in a synthesis (arising from reporting biases). | Assessing reporting bias using funnel plot was not performed in this review. Nevertheless, the authors report the characteristics of all included studies to describe the available evidence.  “These 128 studies covered 29 countries spread across all six geographical regions of the world (Africa, Asia, Australia and Oceania, Europe, Northern America, and Latin America). The first study was published in 1990 and the most recent in 2024. While only 15 studies were published in the first 10 years between 1990 and 2000; the number of published articles remained stagnant each year, at around 6 studies annually.” |
| Item 15. CERTAINTY ASSESSMENT: Describe any methods used to assess certainty (or confidence) in the body of evidence for an outcome. | The authors report the quality assessment of evidence using Mixed Methods Appraisal Tool (MMAT).  “At least two reviewers (LK and SR or LK and MI) assessed and appraised the methodological quality of the studies independently using Mixed Methods Appraisal Tool (MMAT). The types of studies assessed using MMAT include qualitative studies, quantitative randomized controlled trials, quantitative non-randomized studies, quantitative descriptive studies, and mixed-method studies. Five criteria were used to assess the overall risk of bias for each study type. Each criterion is given a rating of ‘yes’, ‘no’, or ‘can’t tell’. For every ‘yes’ answer, the study was given a score of between 20 and 100, with 20 being the lowest and 100 the highest. Any inconsistencies were resolved through discussion and included a third reviewer (SR or MI), if necessary.” |
| Item 16a. STUDY SELECTION: Describe the results of the search and selection process, from the number of records identified in the search to the number of studies included in the review, ideally using a flow diagram. | The authors report results of the search and selection process in text and in a flow diagram (Figure 1).  “From the searched databases, we identified 12,889 records. Of these, 4,009 duplicates were removed. After title and abstract screening, the remaining 426 full-text articles were assessed for eligibility. A total of 298 studies were excluded from the 409 because they did not meet the inclusion and exclusion criteria. That left a total of 128 articles to be considered for final review, including 124 quantitative studies and 4 qualitative studies. The study selection process is shown in Figure 1.” |
| Item 16b. STUDY SELECTION: Cite studies that might appear to meet the inclusion criteria, but which were excluded, and explain why they were excluded. | The authors present the number of excluded studies along with the reasons for their exclusion in Figure 1.  “A total of 295 studies were excluded from the 416 because they did not meet the inclusion and exclusion criteria. That left a total of 127 articles to be considered for final review, including 123 quantitative studies and 4 qualitative studies. The study selection process is shown in Figure 1.” |
| Item 17. STUDY CHARACTERISTICS: Cite each included study and present its characteristics. | The authors include a table presenting for each included study the citation, study design, year published, period of data collection, type of illness, country, and industrial sector in Table 2. |
| Item 18. RISK OF BIAS IN STUDIES: Present assessments of risk of bias for each included study. | The authors present the assessment of risk of bias in the narrative and in details in a table indicating the overall assessment for each study result in the S12, S13, and S14 File.  “The majority of the 127 studies (n=121) used a quantitative descriptive research design, followed by qualitative design (n=4), and quantitative non-randomized design (n=2). The quality of the studies was appraised using MMAT with the highest score of 100. Studies that obtained high scores in terms of quality were qualitative studies, with 50.0% (n = 2) scored 100 and 50.0% (n=2) scored 80. For these qualitative studies, a perfect score (100) indicates that a paper has met all the quality criteria.” |
| Item 19. RESULTS OF INDIVIDUAL STUDIES: For all outcomes, present, for each study: (a) summary statistics for each group (where appropriate) and (b) an effect estimate and its precision (e.g. confidence/credible interval), ideally using structured tables or plots. | The authors present the results in a table for each category based on the data type, type of occupational disease, region, and industrial sector, as shown in Table 1, Table 2, Table 4, and Table 5. |
| Item 20a. RESULTS OF SYNTHESES: For each synthesis, briefly summarise the characteristics and risk of bias among contributing studies. | The authors present the results in a table for each category based on the data type, type of occupational disease, region, and industrial sector, as shown in Table 1, Table 2, Table 4, and Table 5. |
| Item 20b. RESULTS OF SYNTHESES: Present results of all statistical syntheses conducted. If metaanalysis was done, present for each the summary estimate and its precision (e.g. confidence/credible interval) and measures of statistical heterogeneity. If comparing groups, describe the direction of the effect. | The authors present the results in a table for each category based on the data type, type of occupational disease, region, and industrial sector, as shown in Table 1, Table 2, Table 4, and Table 5. Meta-analysis could not be performed in this review. |
| Item 20c. RESULTS OF SYNTHESES: Present results of all investigations of possible causes of heterogeneity among study results. | The authors report conducting meta-analysis was not possible, thus, a narrative synthesis was preferred.  “Due to the diversity in the characteristics of the studies, conducting a meta-analysis was not possible. Thus, a narrative synthesis was performed for this systematic review.” |
| Item 20d. RESULTS OF SYNTHESES: Present results of all sensitivity analyses conducted to assess the robustness of the synthesized results. | The authors report conducting meta-analysis was not possible, thus, a narrative synthesis was preferred.  “Due to the diversity in the characteristics of the studies, conducting a meta-analysis was not possible. Thus, a narrative synthesis was performed for this systematic review.” |
| Item 21. REPORTING BIASES: Present assessments of risk of bias due to missing results (arising from reporting biases) for each synthesis assessed. | Assessing reporting bias using funnel plot was not performed in this review. Nevertheless, the authors report the characteristics of all included studies to describe the available evidence.  “These 127 studies covered 29 countries spread across all six geographical regions of the world (Africa, Asia, Australia and Oceania, Europe, Northern America, and Latin America). The first study was published in 1990 and the most recent in 2024. While only 15 studies were published in the first 10 years between 1990 and 2000; the number of published articles remained stagnant each year, at around 6 studies annually.” |
| Item 22. CERTAINTY OF EVIDENCE: Present assessments of certainty (or confidence) in the body of evidence for each outcome assessed. | The authors report conducting meta-analysis was not possible, thus, assessment of certainty of evidence was not performed. |
| Item 23a. DISCUSSION: Provide a general interpretation of the results in the context of other evidence. | There is no previous evidence regarding the reporting of occupational diseases at the time the manuscript was written. The authors discussed the interpretation of the result in each category. |
| Item 23b. DISCUSSION: Discuss any limitations of the evidence included in the review. | The authors described the limitations of the evidence.  “Data sources were varied and provided little information on the coverage of the working population. We included grey literatures, but non-English publications were not included in the analysis. Excluding non-English publications might have little impact on the conclusions.” |
| Item 23c. DISCUSSION: Discuss any limitations of the review processes used. | The authors report the limitation of the review process used.  “Data sources varied substantially across the studies, providing limited information on the extent to which the working population was covered and demonstrating considerable methodological heterogeneity (e.g., differences in reporting systems, types of occupational diseases, and characteristics of the populations studied). This variation restricted our ability to synthesize the data.” |
| Item 23d. DISCUSSION: Discuss implications of the results for practice, policy, and future research. | In the conclusion, the authors mentioned the implications of the results for policy:  “Our findings encourage policymakers, particularly in LMICs, to establish health information infrastructures for occupational disease reporting that enable data sharing and interoperability between stakeholders, including employers, employees, and physicians.” |
| Item 24a. REGISTRATION AND PROTOCOL: Provide registration information for the review, including register name and registration number, or state that the review was not registered. | The authors report that the review was registered, specifying the register name (PROSPERO) and registration number:  “This study is registered on PROSPERO, number CRD42023417814.” |
| Item 24c. REGISTRATION AND PROTOCOL: Describe and explain any amendments to information provided at registration or in the protocol. | There are differences between protocol and review, as follow:   - Title: The original title of the protocol was “A systematic review of global reporting trends of occupational illnesses”. The title was changed to “Global trends in occupational disease reporting: a systematic review”, as the term “occupational disease” is more familiar than “occupational illness”, and the term “global trends” describes the information better than “global reporting trends” since we also provide information on the number of occupational disease cases, not only the reporting rate. - Objectives: The initial objectives were to answer the following research questions: (1) what is the global reporting and underreporting rate of occupational illnesses according to the country's income level, industrial sector, and type of occupational diseases? and (2) how are occupational illnesses being reported in all countries? There were no changes in the main objective, that is to review the reporting and underreporting of occupational diseases. Nevertheless, in the secondary objective, we changed the objective to identify the factors affecting the underreporting of occupational diseases. We expected that the reporting mechanisms will be included in the main objective. - The review team members: Initially, the review team members were LK, LW, AA. Nevertheless, LW was pulled out and unable to participate before the searching begun. SR and MI joined as the review team members. AA supervised the whole data selection process. |
| Item 25. SUPPORT: Describe sources of financial or nonfinancial support for the review, and the role of the funders or sponsors in the review. | The first author reports receiving funding to conduct her PhD study. This review is part of her PhD study.  Levina C Khoe received a PhD scholarship from the Indonesia Endowment Fund for Education, Ministry of Finance, Government of Indonesia. The research grant is part of her PhD scholarship. All other authors received no financial support for the research, authorship and/or publication of this article. |
| Item 26. COMPETING INTERESTS: Declare any competing interests of review authors. | The authors declare having no competing interests. |
| Item 27. AVAILABILITY OF DATA, CODE AND OTHER MATERIALS: Report which of the following are publicly available and where they can be found: template data collection forms; data extracted from included studies; data used for all analyses; analytic code; any other materials used in the review. | The authors confirm that the data supporting the findings of this study are available within the article and its supplementary materials. |
