# Supplementary material for: Global reporting and underreporting of occupational diseases: A systematic review
Source: PLoS One. 2026 Mar 26;21(3):e0345318. doi: 10.1371/journal.pone.0345318 (PMC13020801; doi:10.1371/journal.pone.0345318)
Supplement: Attachment — Submitted filename: PONE-D-24-50153.docx [file pone.0345318.s016.docx]

1. Abstract
   1.2. Keywords should be modified according to the title and topic of the research.
   2.2. Use the passive voice in writing the abstract and the entire article.
2. Introduction
   2.1 The introduction should provide a more comprehensive overview of the significance of reporting and underreporting of occupational disease, outlining its historical development and current relevance.
       2.2. Include more recent references from the last two years to ensure the literature review is up-to-date.
       2.3. It is best to explain the difference between occupational and non-occupational diseases, as well as the difficulties in distinguishing between the two. In addition, you are expected to give examples and explanations of common occupational diseases.
       2.4. The studies conducted in the study focused mostly on World Health Organization statistics, and it seems that reputable research and articles have not been examined much.
3. Methods

3.1. Include more visual aids, such as charts, graphs, and tables, to illustrate key points and data trends.
3.2. Provide a critical analysis of the reviewed studies, highlighting any methodological weaknesses or biases.

4. Results

4.1. Only two studies on underreporting are mentioned in the study. These two studies alone cannot provide a proper analysis of the current situation and identify the factors influencing this.
4.2. No studies have been reported in the oil, gas, and petrochemical industries, while occupational diseases are common in these industries. What do you think could be the reason for this?

5. Discussion

5.1. Elaborate on the future research directions and gaps identified in the current literature.
5.2. Regarding the classification of occupational hazards, noise is one of the physical hazards that is written separately. It is better to create examples of occupational hazards and their links with occupational diseases. Also, in this section, it is necessary to avoid generalizations and not rely solely on the data of others, and to make sure that the data obtained from this study is interpreted more accurately and clearly.
5.3. What achievements and knowledge have the results of this study added to the studies?.


5.4. At the beginning of the study, two objectives for conducting the study were stated, but appropriate answers for the two objectives were not stated in the continuation. The results regarding the two objectives mentioned need to be carefully examined and interpreted, and compared with other studies.
5.5. Provide a detailed analysis of the limitations and challenges faced in the practical implementation of occupational disease.
5.5. Add a section on the integration new technologies, such as IoT and AI in the field of occupational disease.

6. Conclusion

5.6. Summarize the key takeaways and practical recommendations for policymakers, researchers, and practitioners in the conclusion.
