# Supplementary material for: Global reporting and underreporting of occupational diseases: A systematic review
Source: PLoS One. 2026 Mar 26;21(3):e0345318. doi: 10.1371/journal.pone.0345318 (PMC13020801; doi:10.1371/journal.pone.0345318)
Supplement: Attachment — Submitted filename: Response to Reviewers PONE-D-24-50153_300425.docx [file pone.0345318.s018.docx]

**Response to Reviewers**

Dear Editor,

Thank you for giving us the opportunity to revise the manuscript entitled “Global Trends in Occupational Disease Reporting: A Systematic Review” (PONE-D-24-50153) for publication in the PLOS ONE journal. We appreciate the time and effort that you and the reviewers dedicated to providing feedback on our manuscript. We have carefully reviewed the comments and revised the manuscript accordingly. Our responses are given in a point-by-point manner below. Any changes are highlighted within the manuscript. All page numbers refer to the revised manuscript file. We hope the revised version is now suitable for publication and look forward to hearing from you in due course.

Yours sincerely,

On behalf of all authors,

Levina Chandra Khoe

**Point-by-point response**

**Reviewer #1**

The figures are called out in the main text but listed as S3 appendices--please either put them in the main document or refer to them in the appendix.

Response: Thank you, we have included the figures in the main text.

In your conclusions (abstract) you call for the need for a national system of reporting occupational diseases, do you mean international, so we have a better global count? Or are you meaning each country should establish a national system?

Response: Our study included articles that assessed the burden of occupational diseases at country level (line 141-142). We found no studies from low- and lower-middle-income countries, indicate the need for reporting systems in those countries. Therefore, in the conclusion (abstract), we encourage that every country should establish a national reporting system, which will contribute to the global statistics of occupational diseases.

Background, line 78: Suggest changing wording to say "exposure typically is sourced" from employers. Other times, likelihood of exposure is sourced from a 3rd party (IH for example) especially when its occurring in a court of law

Response: Thanks for your suggestion. We have changed the wording as suggested in the Background, line 89.

Background, line 112: you mention the review will bring together evidence on LMICs, but my understanding is no studies in low income countries were included

Response: Yes, originally our aim was to provide evidence on occupational disease reporting from countries based on their income levels. However, our study found that no studies have been conducted in low income countries. Thus, we changed the wording from “both high-income countries and LMICs” to “all countries” (Background, line 125).

For eligibility criteria, did you include modeling studies?

Response: We focused on the descriptive or explanatory studies that provide real world data at the national level, but not for predicting or forecasting outcomes. Thus, we excluded the modelling studies (added in Eligibility Criteria line 146).

Quality Assessment: more details are needed on the scoring between 20-100. Is there firm criteria or is it semi-quantitative? Given at least two reviewers applied the tool, do you have information on the agreement between the reviewers? Were scores averaged or was the rubric they applied so strict that they had to agree exactly?

Response: Five criteria were used to assess the quality of each study. Studies were scored as follows:

- 0% of quality criteria met if there is no “yes” answer to any of the five criteria;
- 20% of quality criteria met if there is one “yes” answer;
- 40% of quality criteria met if there are two “yes” answers;
- 60% for three “yes” answers;
- 80% for four “yes” answers”; and
- 100% for “yes” answers to all questions.

At the beginning of the appraisal process, two investigators (LK or SR / LK or MI) independently assessed the papers. If different results were obtained, the assigned investigators discussed until an agreement was reached. If no agreement was reached, a third investigator (SR or MI) was consulted to obtain an agreement. The statement about how to resolve any disagreement has been added in the Methods section line 212-214.

Results: Characteristics of included studies may be able to be displayed in a table that number of studies by year, country/region, income, disease, industry sector, etc. This would not be the detail in S2, just a summary as presented in the text.

Response: Thank you for your suggestion. We have incorporated table 1 in the manuscript to depict the characteristics of included studies.

Results line 308: "Occupational-related skin disease was the most reported condition in Europe"--was it the most reported or was it the most studied? Or the condition which had the most included studies? Things like skin disease are easier to study for many reasons than cancers but doesn't mean they are the most common work-related condition. In general, I'd just be careful with this language to make it clear these were the most common studies in the review not necessarily the most common in the population

Response: Thanks for your suggestion. We agree with your suggestion and have changed the wording to “the most studied condition” (line 335).

line 368: Make it clear these are only 2 studies in the review that looked at underreporting, as many other studies have investigated underreporting of occ diseases

Response: In this study, we included only studies that assess the reporting or underreporting of occupational diseases at the country level (line 141-142). Thus, we excluded studies on reporting or underreporting of occupational diseases conducted at the facility or company level. We excluded these studies because we aims to have a summary of global trend of occupational diseases and make a comparison between countries.

I like the inclusion of the exploration of factors affecting underreporting of occupational diseases and am pleased to see that in the paper!

Response: Thank you!

Line 395: is it 128 studies or 127?

Response: Apologies for the mistake, it is 127 studies, as referred to in Figure 1.

Line 411: Was this really the most striking thing?

Response: As the world’s top manufacturer with the largest population, we assumed that China might have a significant burden of occupational diseases, which could be reflected in a higher number of publications on the topic. However, contrary to our expectations, there were only three publications from China, all in Chinese, and none in English. The limited number of publications does not necessarily mean that China has a low burden of occupational diseases; rather, it might indicate that limited research had been done in this area.

Line 456: Is TB, HepB and HepC the most common health concerns, or the most frequently studied? I would think MSDs and stress/mental health would also be extremely common health concerns although less frequently studied. The studies you cite look at the prevalence of these concerns, but this doesn't mean they are the most common, it just means they were studied and you found them in your review.

Response: Thank you for this important comment. We agreed that TB, HepB, and HepC are the most frequently studied and not necessarily the most common health concerns. We changed the sentence accordingly in line 503-504. Nevertheless, previous studies have indicated that healthcare workers are frequently exposed to biological agents, and therefore have a higher prevalence of TB and hepatitis than the general population. We have added additional references to support this argument (Tarantola et al 2006 & Reis et al 2019).

I think it's important to acknowledge your systematic review is far from complete and is looking at diseases that people decided to look at--making claims like "communicable are more likely in Africa" v. "Non-communicable is more likely in Europe" Is a function of what the literature has looked at--the entire scope of possible diseases has not been considered. Using language like, "in our review" or "in the papers included in this review" could help to temper some of the findings

Response: We changed the wording in line 508-516 to avoid misinterpretation of the findings, and used the terms “in our review”, and “frequently studied”.

Nowhere in the discussion is the risk of bias talked about and how this could impact your findings.

Response: We have added a statement in the discussion section (line 515-516) to caution readers to be careful when interpreting the results of this review.

There are lots of places that occupational disease are reported other than in the literature. For example, through ministries/departments of labor. Overall, I think conflating what is in the literature as what is reported is in error (example: line 482).

Response: We agreed that occupational diseases can be reported not only from literatures, but also from government or institution reports. Thus, we included WHO Institutional Repository for Information Sharing (IRIS), Dimensions, and Google Scholar in our searching databases, as described in the Methods section. The result showed that many papers were published from high-and upper-middle-income countries, and none from low- and lower-middle-income countries, indicating a significant gap in the occupational health research.

Reviewer #2:

Hello
This is a good article and a good topic has been chosen.
In my opinion, the conclusion section should be more complete. Currently, only the conclusion has been drawn that the real conditions do not match the existing reports, which we could have predicted almost without conducting this study.
It would be better to provide better solutions based on the results of this study, especially suggestions for conducting future studies.
Good luck.

Response: Thank you for your suggestion. We have added a recommendation to follow the practical guide for establishing a national system for recording and notification of occupational diseases, which was developed by the International Labour Organization (line 569-571).

Reviewer #3:

I carefully reviewed the manuscript. The aim of the manuscript is to give an overall profile of the world occupational diseases' trends via a systematic review. I found this manuscript useful as an acceptable report on the situation of the workforce health all around the world. We know that reporting systems even in High Income Countries are not working good enough and many people lost their health as a result of poor occupational health services in their countries. From this point of view, this manuscript has a great value for sensitize the researchers and policymakers to pay more attention to the subject.

Response: Thank you, we appreciate your thorough review.

Most of my concerns about the rigor of the study are cover and answered in Limitation section. However, authors can strengthen the Conclusion by means of the mapping of the findings on the elements of a good occupational health service including screening, registry, primary health care services, secondary, and third health care services to cover the workforce health. They also can better describe the reasons underlying factors for underreporting the OD cases.

Response: We added some statements regarding the reasons for underreporting and the importance of strengthening occupational health services (line 569-573).

As minor a minor comment, I saw the figure, 127, for the number of the studies considered in Discussion, Line 395. while in the other section, this number is reported 128!

Response: Thanks for your comments. This was a typographical error; the correct number of studies is 127. We have revised this accordingly.

Reviewer #4: Abstract:
In the abstract method section, mention the literature search period to increase transparency.

Response: We added the literature search period in the abstract.

Introduction:
The opening sentence could be stronger by highlighting the direct impact of underreporting on health policies and worker protection.

Response: Thank you for your suggestion. In the Introduction section, we have added line 68-70 to highlight more strongly the impact of occupational diseases on workers’ health. We have also added line 120-122 to highlight the consequences of underreporting, especially its negative impact on the effectiveness of prevention efforts.

Methods:
"This study is registered on PROSPERO, number CRD42023417814."
This sentence appears twice (lines 125 and 122). You can delete one of them to avoid repetitiveness.

Response: We have removed the repeated sentence (line 125 in the original manuscript).

Discussion:
Some sections discuss LMICs in depth, but comparisons with HICs are not always clear. Emphasis could be added on why LMICs lag behind in reporting and how HICs can be a model for improvement.

Response: Thank you for your comments. In the discussion section, we provided arguments regarding the challenges of reporting occupation-related diseases in LMICs (line 418-424). We also added a statement about occupational disease surveillance in the UK and mentioned that this approach could be a good example for LMICs to follow (line 488-491).

Reviewer #5:

All comments upload as an attachment.

1. Abstract
   1.2. Keywords should be modified according to the title and topic of the research.

Response: The following keywords are used in this paper: occupational disease; underreporting; mandatory reporting; workers' compensation. We have added “global trends” to the list. We believe these keywords are relevant to the topic of the research, i.e., underreporting of occupational diseases.

2.2. Use the passive voice in writing the abstract and the entire article.

Response: Thank you for your suggestions. We have revised several sentences in the manuscript using the passive voice, where appropriate. However, we believe that both passive and active voices are important but would be open to further advice from the editors on where further changes should be made.

References:

1. Kojima T, Popiel HA. Proper Scholarly Writing for Non-Native English-Speaking Authors: Choosing Active and Passive Voice, Rewording, and Refining Texts. J Korean Med Sci. 2022 Nov 14;37(44):e312. doi: 10.3346/jkms.2022.37.e312. PMID: 36377291; PMCID: PMC9667014.
2. Millar N, Budgell BS. The passive voice and comprehensibility of biomedical texts: An experimental study with 2 cohorts of chiropractic students. J Chiropr Educ. 2019 Mar;33(1):16-20. doi: 10.7899/JCE-17-22. Epub 2018 Aug 2. PMID: 30070902; PMCID: PMC6417867.

2. Introduction
   2.1 The introduction should provide a more comprehensive overview of the significance of reporting and underreporting of occupational disease, outlining its historical development and current relevance.

2.2. Include more recent references from the last two years to ensure the literature review is up-to-date.

2.3. It is best to explain the difference between occupational and non-occupational diseases, as well as the difficulties in distinguishing between the two. In addition, you are expected to give examples and explanations of common occupational diseases.

2.4. The studies conducted in the study focused mostly on World Health Organization statistics, and it seems that reputable research and articles have not been examined much.

Response: Thank you for your suggestions. We added references from recent publications to provide greater global context on the burden of occupational diseases in the Introduction section (line 60-61). Additionally, we have explained the difference between occupational and non-occupational diseases in the Introduction section (line 60-69). Our review included five electronic databases and three other sources (i.e., WHO IRIS, Dimensions, and Google Scholar) to identify grey literatures. World Health Organization statistics are presented in the Introduction section. We also included studies that used real world data from various countries, as described in the eligibility criteria. The search was updated in September 2024 to ensure all relevant studies are captured.

3. Methods

3.1. Include more visual aids, such as charts, graphs, and tables, to illustrate key points and data trends.

Response: The study selection process is illustrated in Figure 1, depicting the study flow and number of studies in PRISMA flowchart.

3.2. Provide a critical analysis of the reviewed studies, highlighting any methodological weaknesses or biases.

Response: All included studies were critically appraised using the Mixed Methods Appraisal Tool (Quality Assessment subsection in Methods, line 200-209).

4. Results

4.1. Only two studies on underreporting are mentioned in the study. These two studies alone cannot provide a proper analysis of the current situation and identify the factors influencing this.

Response: In our opinion, reporting and underreporting of occupational diseases are two sides of the same coin. The more we know about reporting, the better we understand the degree of underreporting. The two specific studies on underreporting mentioned in this review may not, on their own, provide sufficient insight into the current state of underreporting of occupational diseases and the factors that influence it. However, together with the reporting studies, they offer a more comprehensive insight into the problem of underreporting, which could serve as a basis for discussion and further work in this area. Lastly, the fact that only two studies reported on underreporting of occupational diseases is itself a testament to the need for more work in this area.

4.2. No studies have been reported in the oil, gas, and petrochemical industries, while occupational diseases are common in these industries. What do you think could be the reason for this?

Response: In Table 3, we presented the most reported occupational diseases by industrial sector. We identified musculoskeletal disorders, respiratory disease, and poisoning as occupational diseases reported in the mining and quarrying industries. As we have explained, this systematic review focuses on studies on occupational disease reporting and underreporting at the national level, excluding those conducted at the company or district level. This could partly explain why no studies have been reported in the oil, gas, and petrochemical industries.

5. Discussion

5.1. Elaborate on the future research directions and gaps identified in the current literature.

Response: Our findings indicate that most studies on occupational disease reporting were conducted in high-income countries, and none in low-income countries (line 430-433). We also found that different data sources are used for reporting occupational diseases, as shown in Table 2. In high-income countries, a multisource approach combining data from workers’ compensation, physicians’ report, and labour force surveys is often used. By contrast, in middle-income countries, workers’ compensation databases are heavily relied on. In addition, we found limited studies that assessed all type of occupational diseases at the national level. Many studies focused on certain industrial sectors, particular diseases, or the sub-national level.

Based on these findings, we have recommended that every country develop a national reporting system for occupational diseases to ensure comprehensive and reliable national data. This would facilitate cross-country comparisons and inform occupational disease prevention measures (Conclusion section). Further research testing the implementation of physician reporting on occupational diseases in low- and middle-income countries would be useful to explore factors affecting reporting behaviours. We added this statement in the Discussion section line 491-492. We also recommend that future studies assess the national burden of occupational diseases by triangulating different data sources to improve data accuracy. This statement is added in the Discussion section line 546-547.

5.2. Regarding the classification of occupational hazards, noise is one of the physical hazards that is written separately. It is better to create examples of occupational hazards and their links with occupational diseases.

Response: Thank you for your suggestions. We have revised the statement on occupational hazards and provided examples of these hazards (line 495-499).

Also, in this section, it is necessary to avoid generalizations and not rely solely on the data of others, and to make sure that the data obtained from this study is interpreted more accurately and clearly.

Response: It is necessary to interpret the findings from this review cautiously. We are aware that the our review included articles searched from selected databases, hence, government reports or paper published in local languages are not included. Therefore, we added a statement in the Discussion section to caution readers when interpreting the study findings (line 546-548).

5.3. What achievements and knowledge have the results of this study added to the studies?

Response: This study has contributed to the current global mapping of occupational diseases. It has provided valuable insights into the type of occupational diseases commonly studied in different geographic regions and industrial sectors. In addition, this study, has improved our understanding of the multiple data sources used for reporting occupational diseases in high and middle-income countries. Through this study, we have gained greater understanding of the factors that influence the reporting of occupational diseases.

5.4. At the beginning of the study, two objectives for conducting the study were stated, but appropriate answers for the two objectives were not stated in the continuation. The results regarding the two objectives mentioned need to be carefully examined and interpreted, and compared with other studies.

Response: Our study aimed to assess the global reporting and underreporting rate of occupational diseases and identify the factors affecting the underreporting of occupational diseases. We have presented the results on the number of occupational diseases reported across countries in the Result section (line 315) and the incidence rate of occupational disease (line 316). Findings relating to the underreporting were presented in line 395-396. Together, these findings respond to the first aim of the study (assess the global reporting and underreporting rate of occupational diseases). We have made this clearer in the Discussion section (lines 420-425).

For the second objectives, i.e., to identify the factors affecting the underreporting of occupational diseases, we identified several reasons for underreporting as presented in Table 5 and summarized in the Discussion section (lines 426-427).

5.5. Provide a detailed analysis of the limitations and challenges faced in the practical implementation of occupational disease.

Response: Thank you for your suggestion. Challenges to reporting occupational diseases include limited knowledge about occupational diseases and a lack of awareness of occupational hazards. These challenges have been identified and discussed (see Discussion section line 474-476). Other challenges, such as difficulties in diagnosing and reporting occupational diseases, have been mentioned in lines 481-484. Lastly, the complex reporting procedure and how it hampers the reporting process has been acknowledged in lines 517-526.

5.5. Add a section on the integration new technologies, such as IoT and AI in the field of occupational disease.

Response: Thank you for your suggestion. We have incorporated this in the Discussion section (line 528-535).

6. Conclusion

5.6. Summarize the key takeaways and practical recommendations for policymakers, researchers, and practitioners in the conclusion.

Response: The key takeaways and recommendations are included in the conclusion section. First, we recommend that policymakers establish a conducive infrastructure for reporting occupational diseases that enhances collaboration among stakeholders. This should be complemented by the creation of a national database for occupational diseases (line 561-566). Second, we propose strengthening of occupational health services to encompass health promotion, screening, diagnosing, surveillance, and reporting of occupational diseases (line 575-578). Lastly, we recommend targeted occupational health training for employers and employees to increase their awareness of occupational diseases to achieve better reporting rates (line 578-580).
