# Supplementary material for: Global reporting and underreporting of occupational diseases: A systematic review
Source: PLoS One. 2026 Mar 26;21(3):e0345318. doi: 10.1371/journal.pone.0345318 (PMC13020801; doi:10.1371/journal.pone.0345318)
Supplement: Attachment — Submitted filename: Response_SR paper_PLOS One_260126.docx [file pone.0345318.s020.docx]

Dear Editor,

We appreciate the editor’s detailed comments and feedback regarding our manuscript entitled “Global Trends in Occupational Disease Reporting: A Systematic Review” (PONE-D-24-50153R2). We have carefully considered all suggestions and have made the necessary revisions to strengthen the manuscript. Our responses to each of the editor’s comments are presented in a point-by-point format in the revised submission. We hope the changes made meet your expectations and bring the manuscript in line with the requirements for publication in *PLOS ONE*.

We look forward to hearing from you in due course.

Yours sincerely,

On behalf of all authors,

Levina Chandra Khoe

**Comments from editor:**

Thank you for your latest revisions. We still feel that some of the points raised in the last decision require clarification. Specifically:

Line 198: "the study was given a score of between 20 and 100". Can you please clarify in detail how these scores were assigned?

There are five criteria in each study design category. A “yes” response indicates that the paper provides sufficient information for that criterion. A “no” response indicates that the paper does not report any information relevant to the criterion, while a “can’t tell” response indicates that the available information is unclear or insufficient to determine whether the criterion is met.

Each “yes” answer is assigned a score of 20. A score of 0 is assigned for every “no” or “can’t tell” response.

Did every "yes" answer equate to a score of 20, with a maximum score of 100 for five "yes" answers?

Yes, every “yes” answer equates to a score of 20, with a maximum score of 100 for five “yes” answers.

Please also clarify what those scores represent in terms of assessed quality, and how they were then used in the systematic review. For example, were any studies excluded based on the quality assessment (such as the study that received a score of 0/100)?

A maximum score of 100 indicates that the paper reported all the necessary information for each criterion. Conversely, a score of zero means the paper did not contain any information relevant to any of the criteria. Studies with lower scores are therefore considered to have greater methodological limitations and a higher risk of bias. No studies were excluded from this review based on the quality assessment, as all had already met the inclusion criteria during the screening stage. Thus, the primary purpose of the quality assessment was to determine the trustworthiness and scientific soundness of the included studies. This exercise enabled us to judge the extent to which the findings of each study might have been influenced by poor design or methodological flaws. A significant proportion of studies in this systematic review (over 80%) achieved high quality scores. The figure below displays the **frequency of studies** categorized by their **MMAT quality assessment scores**.

n total = 127 studies

n quantitative descriptive = 121 studies

n quantitative non-randomized = 2 studies

score 80 = 1

score 60 = 1

n qualitative = 4 studies

score 100 = 2

score 80 = 2

2. For the discussion of risk of bias, we mean this in the sense of the risk of bias assessment (also known as quality assessment) that was performed using the MMAT tool. Please discuss how this could impact your findings.

The risk of bias assessment indicates a high level of methodological quality across the included studies, with the vast majority demonstrating rigorous design and implementation. Fewer than 20% of studies scored 60 or below, suggesting only a small proportion with notable methodological limitations. Overall, the potential bias was minimal, supporting the reliability and validity of our findings. To enhance transparency, we have incorporated additional sentences into the Result and Discussion sections to strengthen the reporting of these outcomes.

Result section, page 23 line 7-15

“For these qualitative studies, a perfect score (100) indicates that a paper has met all the quality criteria. Such excellence is reflected in a study design that is fully aligned with the research questions and supported by data collection methods that address those questions comprehensively. These papers also demonstrate strong analytical integrity, ensuring that findings arise directly from the evidence and that all interpretations are substantiated by the data. Moreover, they maintain a coherent and logical connection between the data sources, data collection processes, analytical procedures, and final interpretations. Only two studies were rated as having moderate quality (score = 80), as they provided insufficient information to fully support their reported findings.”

Result section, page 23 line 17-23

“Meanwhile, two quantitative non-randomized studies received a score of 60. Scores in this range generally reflect limited clarity and inadequate explanation of core methodological components. In particular, the absence of detailed information on measurement methods, sampling strategies, and population coverage made it impossible to fully assess the rigor of these studies. These omissions may also limit the generalizability of their findings.”

Discussion section, page 39 line 271-277

“Data sources varied substantially across the studies, providing limited information on the extent to which the working population was covered and demonstrating considerable methodological heterogeneity (e.g., differences in reporting systems, types of occupational diseases, and characteristics of the populations studied). This variation restricted our ability to synthesize the data. Nonetheless, given that most of the studies were of high quality (85.9% scored above 80 out of 100), the findings of this review are considered robust and can be interpreted with a high degree of confidence.”

3. Lastly, please change the title of your manuscript, as the word "trends" does not correspond to the content, as you did not assess trends over time. An appropriate title could be: "Global reporting and under-reporting of occupational diseases: a systematic review".

Response:

We have changed the title accordingly.
