# Supplementary material for: Global reporting and underreporting of occupational diseases: A systematic review
Source: PLoS One. 2026 Mar 26;21(3):e0345318. doi: 10.1371/journal.pone.0345318 (PMC13020801; doi:10.1371/journal.pone.0345318)
Supplement: Attachment — Submitted filename: Response to Decision Letter 081124.docx [file pone.0345318.s017.docx]

**PONE-D-24-40545
Global trends in occupational disease reporting: a systematic review PLOS ONE**

Dear Dr. Khoe,

Thank you for submitting your manuscript for review by PLOS ONE. After careful consideration, we have decided that your manuscript does not meet our standards for systematic and scoping reviews. We are therefore rejecting your manuscript.

PLOS ONE considers systematic reviews and meta-analyses for publication; however, these submissions must address a clear and defined research question; conduct a systematic and comprehensive literature review; and use clearly reported, reproducible, and systematic methods to identify, select, and critically appraise relevant research. Our author guidelines for systematic reviews are at https://journals.plos.org/plosone/s/submission-guidelines#loc-systematic-reviews-and-meta-analyses.

In the case of your submission, we are concerned that the literature review was completed in May 2023. Systematic and scoping reviews should provide an up-to-date overview of the research topic covered. Since the literature search was completed over a year ago, we are concerned that this study does not meet this requirement.

As a result of these concerns, we cannot consider the manuscript for publication at PLOS ONE at this time. However, if you are able to address the concerns discussed above, we may be able to consider a revised version of the manuscript. This would however require a substantial amount of work and thus, would be considered as a new submission. If you are able to revise the manuscript to respond to the concerns noted above, please refer to the original submission in the covering letter and accompany the submission by a rebuttal letter that responds to the comments made on the original manuscript.

I am sorry that we cannot be more positive on this occasion, but I hope you understand the reasons for this decision.

Yours sincerely,

Deepavali A Dhond, Ph.D. Editorial Office
PLOS ONE

**Response to Decision Letter**

Dear Dr Deepavali,

We thank you for the opportunity to review our manuscript entitled “Global trends in occupational disease reporting: a systematic review”, for publication in PLOS One Journal.

We are aware of your concern that the literature review was completed in May 2023. Therefore, we have updated the searching in October 2024, and added recent articles published between 2023 and 2024 to be included in our analysis. As we went through the searching and screening process again, we found there were duplications in data entry into the Covidence. Thus, we revised the number of articles in the searching result and updated in the PRISMA and manuscript. We also added the list of excluded articles in the supporting information. In this submission, we attached the following documents along with this letter:

1. Cover letter (File name: Cover letter – Khoe LC – Plos One – 041124)
2. New manuscript (File name: Manuscript SR OD Report_updated_301024)
3. Supporting information (File name: Update Supporting Information- Khoe LC – PLOS One – 301024)
4. Decision letter (File name: Decision Letter PLOS One 250924)
5. Highlighted manuscript (File name: Manuscript SR OD Report_ 301024_highlighted)
6. Highlighted supporting information (File name: Update Supporting - Khoe LC – PLOS One – 301024_highlighted)
7. PRISMA checklist (File name: PRISMA 2020 Checklist – PLOS One – Khoe LC – 081124)
8. PROSPERO registration (File name: PROSPERO systematic review protocol – occupational illnesses)
9. Preprint version (File name: Preprint medrxiv khoe SR)

We hope the revised version is now suitable for publication and look forward to hearing from you in due course.

Yours sincerely,

On behalf of all authors,

Levina Chandra Khoe
